# Experimental and Computational Study of Novel Pyrazole Azo Dyes as Colored Materials for Light Color Paints

**DOI:** 10.3390/ma15165507

**Published:** 2022-08-11

**Authors:** Sabina Nitu, Marius Silviu Milea, Sorina Boran, Giannin Mosoarca, Alina D. Zamfir, Simona Popa, Simona Funar-Timofei

**Affiliations:** 1 Faculty of Industrial Chemistry and Environmental Engineering, Politehnica University of Timisoara, Bd. V. Parvan, No. 6, 300223 Timisoara, Romania; 2 National Institute for Research and Development in Electrochemistry and Condensed Matter, 300224 Timisoara, Romania; 3 “Coriolan Dragulescu” Institute of Chemistry, Romanian Academy, 300223 Timisoara, Romania

**Keywords:** pyrazole azo dyes, IR spectroscopy, UV-VIS spectroscopy, density functional theory, color investigation

## Abstract

This paper presents the synthesis of eight new pyrazole azo dyes using ethyl 5-amino-3-methyl-1*H*-pyrazole-4-carboxylate as the diazotization component and various active methylene derivatives as coupling components. These new azo dyes were characterized by spectroscopic (FT-IR, UV-VIS), and spectrometric (^1^H NMR, ^13^C NMR, MS) analyses. The dye structures were modeled by the MMFF94s force field and quantum chemical density functional theory (DFT) calculations using the B3LYP functional and the 6-311G(d,p) basis set, in the gas phase. Weak electrostatic hydrogen bonds for the azo and hydrazo dye tautomers were found in the ground state. The CIS, TD (using the B3LYP and M06-2X functionals), and ZINDO methods were used to estimate the dye UV-VIS spectra in ethanol, which were compared with the experimental ones. The *anti*-configuration arrangement of the π-bonds and the presence of the prevalent hydrazo dye tautomer were supported by the computed ^1^H NMR and ^13^C NMR spectra. A good accordance between the experimental and predicted absorption maxima and chemical shifts was observed. Color investigations using the CIEL*a*b* space were conducted for all dyes in powder and for their mixtures in water-based acrylic resins. The results confirm the newly synthesized dyes’ color properties and that they might be used for light color paints in the varnishes industry.

## 1. Introduction

The pyrazole ring is a structural element that can be found in many pharmaceuticals. This is due to an important biological activity and relatively simple synthetic methods. The pyrazole derivatives have proven anxiolytic [1], anti-inflammatory, antimicrobial [2], and tumor growth inhibitory properties [3].

Heterocyclic diazonium salts are generally a class of compounds with significant synthetic potential conferred by their reactivity as electrophilic agents. By coupling reactions with various classes of compounds, they lead to azo compounds. They have practical applications as dyes for synthetic and natural fibers and are important precursors for obtaining a variety of polycyclic compounds, such as pyrazolotriazoles or pyrazolotriazines. Many of these compounds have biological activity and many applications in the field of medicine and pharmacy [4,5].

The color of organic dyes may be linked to their structure and the way the conjugation develops upon the molecules [6,7,8,9,10,11]. The polarization of the molecule also influences dye color [12]. Their coloring properties depend on the dye concentration [13,14]. Some azo reactive dyes containing pyrazole moieties were used in the dyeing of cotton fiber [15]. Color studies could determine the possibility of using such new synthesized pyrazole azo dyes in the film industry for light color paints.

The aim of this paper was the synthesis of eight new pyrazole azo dyes using ethyl 5-amino-3-methyl-1*H*-pyrazole-4-carboxylate as a diazotization component and several active methylene derivatives as coupling components and their characterization using several analytical methods [16]. Another purpose was to confirm the new dyes’ structure via quantum chemical calculations and color studies, as well as to find a possible use for them in the paints and varnishes industry. In addition, we also performed calculation regarding the possible toxicity of these dyes.

Theoretical structural and UV-VIS, ^13^C NMR, and ^1^H NMR spectra results were obtained and compared to the experimental data. The structures of these dyes were modeled using molecular mechanics and quantum mechanics calculations (by the density functional theory approach). The calculated minimum energy azo and hydrazo tautomers of the ground state were used to simulate the UV-VIS, ^1^H NMR, and ^13^C NMR spectra, which were compared with the experimental ones. The CIEL*a*b* color space was used in all color analyses of the newly synthesized dyes. They were performed for the dyes in powder and for their usage as color paints in a water-based acrylic resin. To the best of our knowledge, the CIEL*a*b* color study of such pyrazole azo dyes and the correlation among the substituents with methylene-active groups have not been proposed before.

## 2. Materials and Methods

### 2.1. General Procedure for the Synthesis of the Pyrazole Azo Dyes

The employed reagents were commercial products (Merck, Fluka) used as such.

The preparation of 4-(ethoxycarbonyl)-3-methyl-1*H*-pyrazole-5-diazonium chloride was performed according to the following procedure [17,18].

A mixture of 0.85 g (5 mmol) ethyl 5-amino-3-methyl-1*H*-pyrazole-4-carboxylate (**1**), 1.1 mL water, and 1.5 mL conc. HCl was heated to approximately 40 °C. The mixture was then filtered on active charcoal and the obtained solution was cooled to 0–5 °C. A solution containing 0.36 g (6 mmol) NaNO_2_ in 1.4 mL water was added dropwise under stirring for 15 min. The solution of diazonium salt obtained was treated with active charcoal and cold filtered. Then, it was directly used in the coupling reaction with several components that have active methylene groups.

The coupling reactions were performed as follows: A solution of 5 mmol of active methylene compound dissolved in 10 mL of ethanol was mixed with a solution of 0.03 moles (2.46 g) CH_3_COONa in 5 mL of H_2_O. The obtained solution was cooled in an ice bath and then the freshly prepared diazonium salt solution was added under stirring. As the diazonium salt is added, the coupling product precipitates. After slowly adding the entire amount of diazonium salt solution, the reaction mass was kept under stirring for another 15 min to finalize the coupling reaction. The obtained precipitate was filtered off, washed on the filter, and dried at room temperature.

**Scheme 1:**
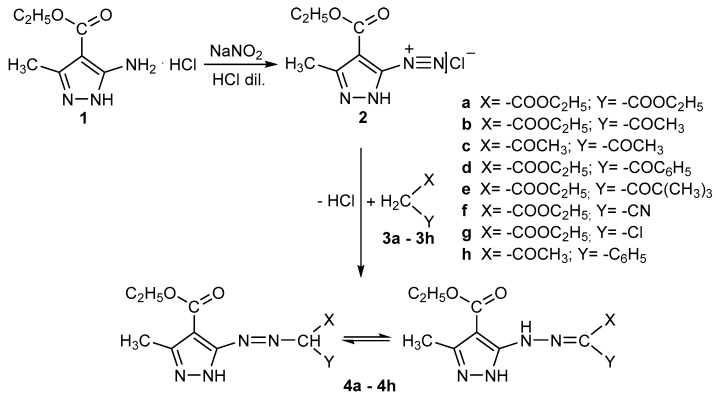
The preparation of 4-(ethoxycarbonyl)-3-methyl-1H-pyrazole-5-diazonium chloride and the coupling reaction with compounds containing active methylene groups.

This reaction may lead to azo derivatives, or corresponding hydrazo tautomers (Scheme 1).

### 2.2. Experimental

*Melting points* were determined on the Böetius PHMK apparatus (Veb Analytik Dresden).

*Thin-layer chromatography* was performed on 60F254 Merck silica gel plates using benzene/methanol = 7:3 (vol) as eluant.

*The ESI-MS mass spectra* of compounds **4a**–**4d**, **4f**, and **4g** were recorded on the Varian 320-MS TQ Mass Spectrometer, using water/acetonitrile/acetic acid = 10/90/0.1 as mobile phase, while for the compounds 4f, 4g, and 4h, the GC-MS mass spectra were recorded on the Agilent G1701DA instrument using methanol as a carrier.

*The FT-IR spectra* were recorded in KBr pellets on the Jascow FT-IR-410 spectrophotometer.

The *UV-VIS* spectra were recorded in ethanolic solution on a Jasco V-530 UV-VIS spectrophotometer.

*^1^H NMR and ^13^C NMR spectra* were recorded on the Bruker Avance AC200 spectrometer at 25 °C in CDCl_3_ or DMSO-*d*_6_ solutions (99.8% atom. %D), using TMS (0.05%, v/v) as a reference, the chemical shifts being expressed in ppm (δ scale) and the coupling constants in Hz (Notations: s—singlet, d—doublet, t—triplet, q—quartet, m—multiplet).

*Color study*: The chemicals used in the color study were the newly synthesized dyes **4a** to **4h** and a water-based acrylic resin that contained 65% titanium dioxide, provided by AZUR S.A., Romania. Each dye, at different concentrations (2%, 5%, 8%, and 15%), was mixed with the acrylic resin and then applied on a cellulosic support (natural wood).

The CIEL*a*b* color parameters of these samples were determined using the MINOLTA CM 3220d spectrophotometer using the CIE D65 illuminant (natural day light) and the standard 10° observer function. The coordinates of the color space are L*, lightness; a*, the transition from green (−a*) to red (+a*); and b*, the transition from blue (−b*) to yellow (+b*).

### 2.3. Theoretical Study

#### 2.3.1. Computational Analysis of Dye Structures

The Marvin Sketch program (Marvin Sketch 20.19.0, 2021, ChemAxon Ltd., Budapest, Hungary, http://www.chemaxon.com (accessed 15 December 2021)) was employed to build the pyrazole azo structures. They were pre-optimized by the OMEGA software (OMEGA version 4.0.0.4 OpenEye Scientific Software, 2020, Santa Fe, NM, USA, http://www.eyesopen.com (accessed 15 December 2021)) [19], using the 94s variant of the MMFF (Merck molecular force field) [20]. The generated conformations were chosen based on an energy cutoff of 25 kcal/mol. The root-mean-square deviation (RMSD) values less than 0.5 Å were used to eliminate similarly shaped structures.

The conformations of minimum energy of the azo and hydrazo tautomers of the pyrazole azo dyes thus obtained were further optimized using the DFT approach [21] at the B3LYP [22,23] level using the 6-311G(d,p) [24,25] basis set, in the gas phase. The structures optimization of the pyrazole azo dyes was performed using the Gaussian 09 Revision B.01 package [26].

The frequency calculations confirmed that all the dye structures optimized in the ground state were true minima. ZPE (zero-point vibrational energy) corrections were calculated with unscaled frequencies.

Global quantum chemical descriptors were calculated for the azo and hydrazo ground state tautomers of the pyrazole azo dyes: the highest occupied (E_HOMO_) and lowest unoccupied (E_LUMO_) molecular orbital energies, the chemical potential (μ) [27], the chemical hardness (η) [28], the electrophilicity index (ω) [29], and the softness (σ) [28,30].

#### 2.3.2. UV-VIS, ^1^H NMR, and ^13^C NMR Spectra Simulation

Optimized ground state geometry of the azo and hydrazo tautomers of the pyrazole azo dyes was employed in the calculation of the ultraviolet-visible spectra. UV-VIS transitions of the new pyrazole azo dyes were simulated using the configuration interaction singles (CIS) [31], the time-dependent (TD) [32], and the Zerner’s intermediate neglect of differential overlap (ZINDO) [33] methods. The integral equation formalism variant (IEFPCM) of the polarizable continuum model (PCM) [34] was employed to validate the maximum wavelengths determined experimentally. The B3LYP approach and the 6-311G(d,p) basis set were employed in the DFT calculations starting from the ground state conformers of minimum energy to simulate the UV-VIS spectra in ethanol (used as solvent), to get more accurate absorption bands of both azo and hydrazo tautomers for each dye. The M06-2X functional [35] gives good long range charge transfer results and was employed utilizing the TD method at the 6-311G(d,p) level of theory, also in an ethanol solution. Eight singlet excited states were calculated for each approach. The computed maximum absorption peaks were compared with the experimental UV-VIS spectra.

The ^1^H NMR and ^13^C NMR chemical shifts were simulated using the gauge-including atomic orbital (GIAO) [36,37] approach and the integral equation formalism variant (IEFPCM) of the polarizable continuum model (PCM). The dimethylsulfoxide and chloroform were used as solvents for the minimum energy conformers of the azomethylene dye tautomers derived from the B3LYP calculations. The simulated GIAO ^13^C NMR chemical shifts were calculated considering the absolute shielding of tetramethylsilane (TMS) of 185.4 ppm [38]. The ^1^H NMR chemical shifts were obtained from the computed magnetic shielding constants using the lowest proton scaling factor approach for the solvent [39].

#### 2.3.3. Toxicity Simulation

The oral rodent acute (LD_50_) toxicity and the corresponding toxicity classes (according to the Globally Harmonized System of Classification and Labeling of Chemicals, https://www.osha.gov/hazcom, accessed 20 July 2022), the hepatotoxicity, and other toxicity endpoints (carcinogenicity, cytotoxicity, mutagenicity, immunotoxicity) of the new pyrazole azo dyes were evaluated using the ProTox-II software (https://tox-new.charite.de/protox_II/index.php?site=home (accessed 20 July 2022)) [40,41]. The bioconcentration factor (BCF) is an ecotoxicity characteristic used to evaluate the bioaccumulative potential of a substance, suitable to screen the bioconcentration in lipids of organisms present in the environment. Chemicals with high BCF values are less soluble in water and are expected to bioconcentrate in aquatic organisms. The pyrazole azo dye BCF values were calculated using the EPI Suite software [42].

## 3. Results and Discussions

### 3.1. Synthesis of the Pyrazole Azo Dyes

The synthesis of the new pyrazole azo dyes involves coupling reactions of 4-(ethoxycarbonyl)-3-methyl-1*H*-pyrazole-5-diazonium chloride with several components that have active methylene groups at equimolar ratios in aqueous-alcoholic solution.

**Scheme 2:**
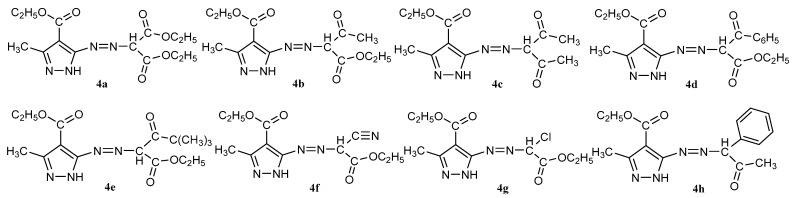
The azo structure of the new pyrazole azo dyes **4a**–**4h**.

The newly synthesized symmetrical pyrazole azo dyes (Scheme 2) were characterized by spectroscopic (FT-IR, UV-VIS) and spectrometric (^1^H NMR, ^13^C NMR, MS) examinations (see the Supplementary Materials).

### 3.2. FT-IR Spectra

FT-IR spectra confirmed the structure of the synthesized compounds. Thus, the three to five weak or medium bands present in the range of 3400 to 3190 cm^−1^ are associated with the N-H stretching vibration, $\nu_{N-H}$. The 1–3 bands of lower intensity at 3440–3300 cm^−1^ are assigned to the H-N bond in hydrazo tautomeric form (C_5Pyr_−NH−N=C<), while the other 2–3 bands, located at a lower wave number, at 3193–3197, 3139–3144, and 3104–3124 cm^−1^, correspond to the H-N bond in pyrazole [43].

**Figure 1:**
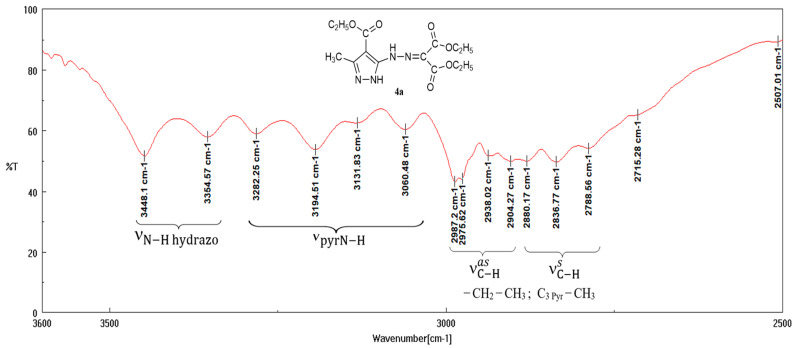
Enlarged (3600–2500cm^−1^) FT-IR spectrum of **4a**.

In order to confirm the presence of the hydrazo tautomer in the series of synthesized dyes, we presented enlarged FT-IR spectra in the range of 3600–2500 cm^−1^ (Figure 1). Only in the IR spectra of compounds **4a**, **4e**, **4f**, and **4g** do they appear distinctly, as sharp bands characteristic of the hydrazo form probably associated intramolecularly with >N–H·····O=C(OEt)–C_4_-pyrazole; in the other spectra, **4b**, **4c**, **4d**, and **4h**, these bands appear as shoulders overlapping/super-imposed over a broad band, which also indicates the intermolecular associations [44,45] (See Table S8, Supplementary Materials).

The other absorption bands corresponding to the hydrazo tautomer are: the stretching vibration of the C=N double bond $\nu_{C=N}$ at 1620–1670 cm^−1^, the stretching vibration of the pyrazole C-N single bond, $\nu_{C_{5\mathrm{Pyr}}-N\left(H\right)}$, with two intense bands at 1250–1315 and 1320–1345 cm^−1^, the bending in-plane deformation of the N-H bond, $\delta_{\mathrm{NH}}$, at 1490–1580 cm^−1^ (weak-medium bands difficult to detect), and scissoring out-of-plane vibration of the N-H bonds, $\gamma_{N-H}$, at 700–750 cm^−1^ (medium band). Except for the two intense bands, $\nu_{C_{\mathrm{Pyr}}-N}$, at 1250–1315 and 1320–1345 cm^−1^, the other bands mentioned above are uncharacteristic, overlapping by the corresponding bands of the ester groups ($\nu_{C=O};\;\nu_{\left(O\right)C-O};\;\delta_{\mathrm{NH}}$) and planar vibrations of the pyrazole ring (${\mathrm{Sk}}_{\mathrm{pyr}}\equiv \nu_{C=C}+\nu_{C=N}+\delta_{\mathrm{NH}}$).

The presence of the azo tautomer is more difficult to detect by IR spectroscopy, because the specific absorption band of the anti azo group $\nu_{N=N}$ at 1400–1420 cm^−1^ is weak or medium, not characteristic for azo dyes with aromatic heterocyclic structures that also contain alkyl groups. However, in the expanded/enlarged FT-IR spectra in the range of 3600–2500 cm^−1^, this weak-medium band is present and visible in compounds **4a** (medium), **4b** (weak), **4c** (weak), **4e**, **4f**, and **4g** (medium). This information delivered by FT-IR spectroscopy highlighted the presence of azo-hydrazo tautomers, confirming the structure of newly synthesized dyes.

The antisymmetric and symmetric stretching vibrations of the aliphatic tetrahedral C-H bond CH3 and CH2 groups are observed between 2985 and 2920 cm^−1^, $\nu_{C-H}^{as}$, and 2870–2830 cm^−1^, $\nu_{C-H}^{s}$. The very intense bands between 1715 and 1665 cm^−1^ are assigned to stretching vibration of the C=O double bond $\nu_{C=O}$ in both C4-pyrazole and 2-methylhydrazono ethoxycarbonyl groups as well as acetyl and pivaloyl ones. The two intense bands at 1570–1552 cm^−1^ and 1548–1538 cm^−1^ are assigned to the planar vibrations of the pyrazole ring, ${\mathrm{Sk}}_{\mathrm{pyr}}\equiv \nu_{C=C}+\nu_{C=N}+\delta_{\mathrm{NH}}$. The few very weak bands below 2020 cm^−1^ and up to 1800 cm^−1^ are combination bands and higher harmonic bands specific to aromatic and heterocyclic compounds with aromatic character. Moreover, in the two compounds with the phenyl group are present the medium bands at 1615–1600 cm^−1^ assigned to the planar vibrations of the aromatic ring, ${\mathrm{Sk}}_{\mathrm{ar}.}\equiv \nu_{C=C}+\delta_{\mathrm{CHar}.}$. The medium band located at 2209 cm^−1^ is assigned to the nitrile group, $\nu_{C\equiv N}$.

**Figure 2:**
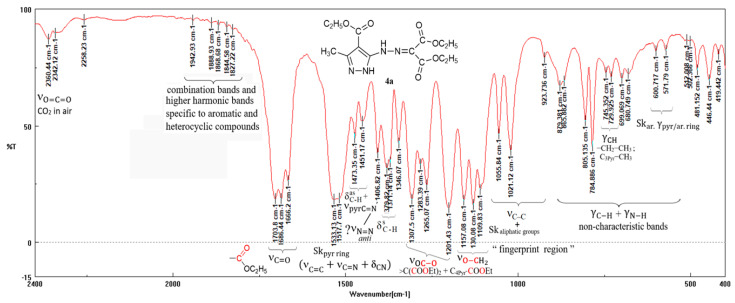
Enlarged (2400–400cm^−1^) FT-IR spectrum of **4a**.

The medium bands at 1475–1465 cm^−1^ and 1450–1435 cm^−1^ are assigned to an antisymmetric bending in-plane deformation of the aliphatic tetrahedral C-H, $\delta_{C-H}^{\mathrm{as}}$+ $\nu_{\mathrm{pyrC}=N}$, and at 1376–1372 cm^−1^ assigned to a symmetric bending in-plane deformation of the aliphatic tetrahedral C-H bond $\delta_{C-H}^{s}$ aliphatic; the weak-medium bands at 1050–1010 and 970–950 cm^−1^ are not characteristic ones, being attributed to stretching vibration of the aliphatic tetrahedral C- bond, $\nu_{C-C}$, to skeletal vibrations for aliphatic groups, and a medium band at 740–710 cm^−1^ assigned to scissoring out-of-plane deformation for the aliphatic groups CH_3_ and CH_2_, $\gamma_{\mathrm{CH}}$. In a few spectra, a weak-medium band at 1420–1410 cm^−1^ is present, assigned to anti azo group $\nu_{N=N}$. The intense or very intense bands at 1345–1320, 1318–1280, and 1200 cm^−1^ are assigned to stretching vibration of the carbon-oxygen single bond $\nu_{\mathrm{OC}-O}$ in methylene + C4-pyrazole carboxyethyl ester groups and the stretching vibration of the pyrazole carbon-nitrogen single bond, $\nu_{C_{5\mathrm{Pyr}}-N\left(H\right)}$, respectively. Moreover, the medium or intense bands at 1160–1100 cm^−1^ are assigned to stretching vibration of the aliphatic tetrahedral C-O bond $\nu_{O-{\mathrm{CH}}_{2}}$ in esters. The medium bands at 878–864 and the weak-medium bands 785–713 and 660–620 can reasonably associate with the scissoring out-of-plane vibration of the C-H and N-H bonds, $\gamma_{C-H}$ and $\gamma_{N-H},$ as well as with that of the carbon skeleton of the pyrazole and phenyl ring, ${\mathrm{Sk}}_{\mathrm{ar}.}\equiv \gamma_{\mathrm{pyr}/\mathrm{ar}.\;}$ (Figure 2).

### 3.3. Computational Analysis of Dye Structures

Several possible configurations of the new pyrazole azo synthesized dyes were calculated using the MMFF94s force field for each dye tautomer. The minimum energy structures were further geometry optimized using the B3LYP approach at the 6-311G(d,p) level, using the Gaussian 09 program [24]. The azo (having the *anti*-configuration form of the azo group) and hydrazo tautomer structures (Table S1, Supplementary Materials) and their total calculated energies in the gas phase are presented in Table S2 of the Supplementary Materials. The B3LYP approach indicates the most stable ground state hydrazo tautomer in the *anti*-configuration form. The conformers having the lowest calculated total energies were further used in spectral simulations.

Intramolecular hydrogen bonds (H-bonds) are present in the optimized structures of both azo and hydrazo dye tautomers (Table S1, Supplementary Materials). Hydrogen bonds were noticed in the case of the azo tautomers of the **4a**, **4e**, **4g**, and **4h** dyes and all hydrazo tautomers (Table S1, Supplementary Materials). H-bond energy was calculated by the VEGA ZZ v. 3.0.1 software (Istituto di Chimica Farmaceutica e Tossicologica "Pietro Pratesi", Milano, Italy) [46] using the CHARMM force field. The classification of hydrogen bonds according to the H-bond energies proposed by Jeffrey [47] is accepted by the broad community of scientists [48,49]. Jeffrey categorized the strength of H-bonds as weak interactions for values of the H-bond energy between 1 and 4 kcal mol^−1^, moderate between 4 and 15 kcal mol^−1,^ and strong from 14 to 40 kcal mol^−1^, respectively. One-center proton donor, A, and one-center proton acceptor, B, were noticed for most of the A–H⋯B hydrogen bonds in the azo and hydrazo tautomers, except the hydrazo tautomer of 4a, which had a three-center (bifurcated donor) [50] hydrogen bond.

Crystallographic X-ray data of some pyrazole, triazole, and tetrazole azo dyes were reported in azo-hydrazo tautomerism studies [51,52]. Intramolecular hydrogen bonds are present in most of the dyes included in these studies. H-bond parameters of the new azo dyes were compared to those of the dyes reported in the above mentioned articles: the N–H ····· A, (A=O or N atoms) hydrogen bond distance values were between 2.62 Å and 2.73 Å in the case of the azo tautomers (for A=N) (and between 2.27 Å and 2.34 Å for the azo and 2.62 Å for hydrazo tautomers [51,52]), between 1.86 Å and 2.2 Å (for A=O) (and between 1.83 Å and 2.06 Å [51,52]) for the hydrazo tautomers. The donor-acceptor H-bond lengths (d_N(H)…O_ for hydrazo and d_(H)N…N_ for azo tautomers) had values between 2.35 Å and 2.43 Å (and between 2.37 Å and 2.87 Å [51,52]) for the azo, and between 2.58 Å and 2.87 Å (and between 2.55 Å and 2.77 Å [51,52]) for the hydrazo tautomers, respectively. The hydrogen bond angle values were in the range between 61.66° and 63.39° (and between 64.18° and 120.44° [51,52]) for the N_pyrazole_HN_azo_ angles of azo tautomers, and between 116.53° and 124.89° (and between 125.95° and 140.6° [48,49]) for the NHO angles of the hydrazo tautomers. The H-bond energy was calculated for the azo dye crystal structures reported in the literature [51,52] using the VEGA ZZ software. The H-bond energy values were between −0.7 and −0.87 kcal mol^−1^ for the hydrazo, and between −0.18 and −0.22 kcal mol^−1^ for the azo tautomers, respectively. The calculated H-bond energy values of the eight new dyes were between −0.19 kcal mol^−1^ and −0.45 kcal mol^−1^ for the hydrazo and between −0.20 kcal mol^−1^ and −0.25 kcal mol^−1^ for the azo tautomers, respectively (Table S1, Supplementary Materials). Therefore, weak electrostatic intramolecular hydrogen bonds for both azo and hydrazo tautomers were found for all eight new azo dyes and azo dye crystal structures reported in the literature. Close values of the H-bond parameters of the newly synthesized azo dyes are noticed compared to those derived from the azo dye X-ray crystallographic data.

The analysis of the calculated dye global quantum chemical descriptors (Table S2, Supplementary Materials) confirmed the information on the hydrogen bonding of the dye tautomers presented above. They also give information on the potential chemical resistance to change the number of electrons among the dye tautomers and the tautomer stability, respectively. Generally, the negative values of the calculated energy of the minimum energy conformers in the ground state in the gas phase were higher in the case of the more stable hydrazo tautomers compared to the corresponding azo ones for each dye. The inspection of the higher calculated electronic chemical potential values for all hydrazo tautomers compared to the azo ones indicates electron donor and electron acceptor abilities for the hydrazo and azo tautomers, respectively. Therefore, electrostatic interactions would be expected in the case of the hydrazo forms, this fact being in agreement with the calculated hydrogen bond data presented above.

The chemical hardness (η) [53,54] is a measure of how easily molecular electron density can be changed. The harder the molecule, the more it will resist accepting or donating an electron charge. Higher negative values of chemical hardness were observed in the case of the azo tautomers, which could be considered harder than the hydrazo ones. These last tautomers would be more polarizable, and more easily involved in charge transfer reactions. The azo tautomers could be considered hard structures, electron acceptors, and less reactive.

Softness (σ) is a characteristic of molecules that evaluates the magnitude of chemical reactivity [55]. It is calculated as the reciprocal of hardness, measures the easiness of charge transfer, and is associated with a high polarizability. Hydrazo tautomers which have higher softness values compared to the azo ones and a smaller E_HOMO_-E_LUMO_ gap would have their electron density changed easier than the azo tautomers.

The electrophilicity index (ω) [29] determines the decrease of energy due to maximal electron movement between donors and acceptors. The electrophilicity index is a reactivity parameter that can quantitatively classify the global electrophilic nature of a molecule within a relative scale [56]. This index evaluates if a system is energetically stable by an optimal electronic charge transfer to it from the environment [57]. A more stable molecule, obtained by an electron transfer to it, corresponds to higher ω values. Higher electrophilicity index values are noticed in the case of the azo compared to the hydrazo tautomers for each dye.

### 3.4. Experimental and Computed ^1^H NMR and ^13^C NMR Spectra

The ^1^H NMR experimental spectra (δH ppm, DMSO-d_6_/CDCl_3_, operating frequency 200 MHz) revealed the characteristic signals for the 4-ethoxycarbonyl-3-methyl-1*H*-pyrazole ring (1.3 (3H, t, *J* = 6.9–7 Hz, CH_3_-CH_2_-O-CO-C_4Pyr_); 2.4–2.5 (3H, s, CH_3_-C_3Pyr_); 4.3 (2H, q, *J* = 6.9–7 Hz, CH_3_-CH_2_-O-CO-C_4Pyr_); 12–13.3 (1H, b.s, H-N_1_)) and for the C_5 Pyr_-((X- and Y-substituted ethylidene)hydrazinyl) group at 1*H*-pyrazole (9.5–10 in DMSO-d_6_/7–7.5 in CDCl_3_ (1H, b.s, X(Y)C=N-NH-C_5Pyr_)) or for its azo tautomeric form (3.4–3.6 (1H, s, X(Y)CH-N=N-C_5Pyr_)), as well as signals characteristic of protons in X and Y groups.

^13^C NMR experimental spectra (δC ppm, DMSO-d_6_/CDCl_3_, operating frequency 50 MHz) also revealed the characteristic signals for the 4-ethoxycarbonyl-3-methyl-1*H*-pyrazole ring (12–13.5 (CH_3_-C_3Pyr_); 14–15 (CH_3_-CH_2_-O-CO-C_4Pyr_); 60–61 (CH_3_-CH_2_-O-CO-C_4Pyr_); 162–163 (CH_3_-CH_2_-O-CO-C_4Pyr_); 93–98 (C_4Pyr_); 139–145 (C_3Pyr_); 150–155 (C_5Pyr_)) and for the 5-substituted hydrazinyl group at 1*H*-pyrazole (130–138 (X(Y)C=N-NH-C_5Pyr_)) or for its azo tautomeric form (75–95 (X(Y)CH-N=N-C_5Pyr_)).

Generally, the chemical shift values of ^13^C NMR for aromatic carbon atoms have values higher than 100 ppm, and the chemical shift values of ^1^H NMR are in the range of 1–10 ppm [58]. Experimental signals observed in the range of 107.8–139.92 ppm correspond to the carbon atoms of the azomethylene group. The carbon atoms belonging to the pyrazole carbon atoms linked to the azomethylene group have chemical shift values between 149.35 and 152.8 ppm. The experimental and computed ^13^C NMR chemical shifts are measured in deuterated dimethylsulfoxide (d6-DMSO) and deuterated chloroform (CDCl_3_) solvents.

The carbon chemical shift values were calculated starting from the azo and hydrazo optimized tautomer structures of the ground state, using the SCF magnetic shielding values calculated using the B3LYP functional by the GIAO method (Table S3, Supplementary Materials). They were calculated from the GIAO isotropic shielding tensors taking into account the absolute shielding of tetramethylsilane (TMS) of 185.4 ppm [38]. The results of the predicted chemical shift values of the carbon azomethylene group and the pyrazole carbon atom attached to the azomethylene group confirm the good agreement with the experimental ones, at the B3LYP functional and 6-311G(d,p) level for the hydrazo optimized tautomers.

The experimental ^1^H NMR spectra of pyrazole azo dyes (measured in d6-DMSO and CDCl_3_ solvents) show a signal for the hydrogen atom attached to the hydrazo group between 7.07 and 10.92 ppm. Closer predicted ^1^H chemical shift values of the hydrogen azomethylene group present in the hydrazo tautomers to the experimental ones were noticed for the **4d**, **4e**, **4f**, and **4g** dyes.

### 3.5. Experimental and Simulated UV-VIS Spectra

The experimental UV-VIS spectra (Table S4, Supplementary Materials) display an intense absorption characteristic in ethanol, located in the range of 312–359 nm, which could be attributed to the azo chromophore, and other absorption peaks, which could correspond to the 3-methyl-1*H*-pyrazole chromophore in the range of 216–223 nm, and the ethyl 3-methyl-1*H*-pyrazole-4-carboxylate chromophore in the ranges of 235–238 nm and 285–293, respectively.

The calculated UV-VIS oscillator strengths and wavelengths (λ_max_) of the new pyrazole azo derived from the CIS, TD, and ZINDO approaches are presented for eight excited states (Table S5, Supplementary Materials).

Both the polarization and diffuse functions are recommended in the prediction of UV-VIS spectra [59] because the polarization functions have the tendency to undervalue the maximum absorption peaks [60]. The configuration interaction singles (CIS), time-dependent (TD), and Zerner’s intermediate neglect of differential overlap (ZINDO) calculations were simulated at the B3LYP/6-311G(d,p) level to simulate the UV-VIS spectrum of the minimum energy tautomer for each dye. The calculated absorption maxima (λ_max_) and the oscillator strengths of the pyrazole azo in both tautomeric hydrazo and azo forms are summarized in Table S5, Supplementary Materials.

Solvent polarity could influence the equilibrium between the dye azo and hydrazo tautomers. The higher the value of a solvent’s dielectric constant, the more polar it will be. It was observed that hydrazo tautomers are preponderant when the solvent dielectric constant raises [61]. Therefore, ethanol, a polar solvent, with the dielectric constant value of 24.85, could favor the spectroscopic results of the hydrazo tautomer.

Dye **4a** shows three maximum experimental wavelengths, at 216 nm, which correspond to the 3-methyl-1*H*-pyrazole chromophore, at 345 nm, corresponding to the azo group, and a local maximum characteristic at 239 nm, for the chromophore ethyl-(3-methyl-1*H*-pyrazole-4)-carboxylate. In the calculated UV-VIS spectra, the following significant absorption maxima were observed: 222.27 nm (azo, CIS), 240.06 nm (hydrazo, CIS), 293.48 (azo, TD), 240.96 nm (hydrazo, TD), 288.72 nm (azo, ZINDO), 235.78 nm (hydrazo, ZINDO), 308.4 nm (azo, CIS), 344.99 nm (hydrazo, TD), 334.21 nm (azo, ZINDO), and 336.04 nm (hydrazo, ZINDO). The M06-2X functional gave the following significant maximum absorption peaks: 223.89 nm (azo, TD) and 246.21 nm (hydrazo, TD). According to the simulated maximum wavelengths, the experimental 216 nm absorption peak would be present in the azo tautomer and the 239 nm and 345 nm peaks would correspond to the hydrazo and the azo forms, respectively. The additional simulated peak of 336.04 nm corresponding to the –NH–N= group was derived for the hydrazo tautomer from ZINDO simulations.

In the experimental UV-VIS spectrum of dye **4b**, the following absorption maxima were noticed: 235 nm corresponding to the chromophore ethyl 3-methyl-1*H*-pyrazole-4-carboxylate and 322 nm for the azo group. In the simulated spectra, the following maximum peaks were observed: 226.16 nm (azo, CIS), 258.87 nm (hydrazo, CIS), 281.53 nm (azo, TD), 239.35 nm (hydrazo, TD), 265.3 nm (hydrazo, ZINDO), 326.05 nm (azo, TD), 361.9 nm (hydrazo, TD), 337.06 nm (azo, ZINDO), and 366.86 nm (hydrazo, ZINDO). The absorption maxima of 229.79 nm (azo, TD) and 259.72 nm (hydrazo, TD) were obtained by the M06-2X functional. The experimental 235 nm maximum wavelength would be present in the azo dye form (as simulated by the CIS sand TD methods) and the maximum absorption peak of 322 nm corresponds to the azo group (as resulted from TD and ZINDO calculations). The calculated peaks at 258.87 nm (by CIS), 259.72 nm (by TD), and 265.3 nm (by ZINDO), which would correspond to the chromophore ethyl 3-methyl-1*H*-pyrazole-4-carboxylate in the hydrazo tautomer, were not confirmed experimentally.

The following maximum absorption peaks were observed in the experimental UV-VIS spectrum of **4c** dye: 238 (local maximum) and 260 nm (local maximum) for the chromophore ethyl 3-methyl-1*H*-pyrazole-4-carboxylate and 331 nm for the azo group. In the simulated spectra, the following were obtained: 238.32 nm (azo, CIS), 263.21 nm (hydrazo, CIS), 282.12 nm (azo, TD), 248.11 nm (hydrazo, TD), 241.69 nm (hydrazo, ZINDO), 291.99 nm (azo, ZINDO), 263.21 nm (hydrazo, ZINDO), 342.93 nm (azo, TD), 365.55 nm (hydrazo, TD), 335.17 nm (azo, ZINDO), and 376.48 nm (hydrazo, ZINDO). Using the M06-2X functional, the following significant maximum absorption peaks were obtained: 233.49 nm (azo, TD) and 263.68 nm (hydrazo, TD). The experimental absorption peak of 238 nm was confirmed for the azo tautomer by the CIS and TD methods, and that of 260 nm by the CIS and ZINDO approaches for the hydrazo dye form. The presence of the azo tautomer with the maximum absorption peak of 331 nm was modeled by the TD approach. The hydrazo azo group was simulated by the TD and ZINDO methods and was not confirmed experimentally.

For **4d** dye, the experimental UV-VIS spectrum showed the following wavelengths: 236 nm (local maximum) for the chromophore ethyl 3-methyl-1*H*-pyrazole-4-carboxylate and 330 nm for the azo group. The following calculated wavelengths were obtained: 226.33 nm (azo, CIS), 220.34 nm (azo, CIS), 217.35 nm (azo, CIS), 215.54 nm (hydrazo, CIS), 248.71 nm (hydrazo, CIS), 246.77 nm (hydrazo, CIS), 293.06 nm (azo, TD), 307.84 nm (azo, TD), 288.27 nm (azo, ZINDO), 265.5 nm (azo, ZINDO), 266.21 nm (hydrazo, ZINDO), 265.14 nm (hydrazo, ZINDO), 346.35 nm (azo, TD), 358.11 nm (hydrazo, TD), 334.69 nm (azo, ZINDO), 326.09 nm (azo, ZINDO), and 354.61 nm (hydrazo, ZINDO). The TD results for the M06-2X hybrid were: 230.78 nm (azo, TD) and 252.52 nm (hydrazo, TD). The 236 nm absorption wavelength could correspond to the simulated spectra performed by the CIS and TD approaches for the azo tautomer. The 330 nm peak is in agreement with the simulated peaks by the ZINDO method for the azo tautomer. The corresponding simulated peaks of 266.21 nm and 265.14 nm (by ZINDO) and 252.52 nm (by TD), which would correspond to the hydrazo form, were not present in the experimental UV-VIS spectrum, and neither were those for the –N(H)-N= (hydrazo azo) group in the tautomer (358.11 nm by TD and 354.61 nm by ZINDO).

Maximum absorption experimental peaks for **4e** dye were: 212 nm (local maximum), corresponding to the 3-methyl-1*H*-pyrazole chromophore, 230 nm (local maximum) for the ethyl 3-methyl-1*H*-pyrazole-4-carboxylate chromophore, 285 nm for the chromophore ethyl 3-methyl-1*H*-pyrazole-4-carboxylate, and 358 nm for the azo group. The computed wavelengths were: 221.51 nm (azo, CIS), 245.84 nm (hydrazo, CIS), 292.1 nm (azo, TD), 239.08 nm (hydrazo, TD), 286.02 nm (azo, ZINDO), 239.04 nm (hydrazo, ZINDO), 344.91 nm (azo, TD), 351.97 nm (hydrazo, TD), 332.56 nm (azo, ZINDO), 323.07 nm (azo, ZINDO), and 349.31 nm (hydrazo, ZINDO). The absorption maxima obtained by the M06-2X functional were: 231.52 nm (azo, TD) and 249.82 nm (hydrazo, TD). The experimental absorption peak of 230 nm was simulated by the CIS, TD, and ZINDO approaches for the hydrazo tautomer. The experimental maximum wavelength of 358 nm would correspond to the –N(H)–N= group in the hydrazo tautomer, simulated by the TD and ZINDO approaches, and that of 285 nm to the azo tautomer (by TD and ZINDO). The experimental local maximum absorption peak of 212 nm had a corresponding wavelength calculated by the CIS method for the azo tautomer.

The experimental wavelengths for **4f** dye were: 216 nm (local maximum) for the 3-methyl-1*H*-pyrazole chromophore and 347 nm for the azo group. The calculated wavelengths were: 205.31 nm (azo, CIS), 234.9 nm (azo, CIS), 259.71 nm (hydrazo, CIS), 294.92 nm (azo, TD), 240.67 nm (hydrazo, TD), 242.94 nm (hydrazo, ZINDO), 266.84 nm (hydrazo, ZINDO), 359.97nm (hydrazo, TD), 329.99 nm (azo, ZINDO), and 366.71 nm (hydrazo, ZINDO). The following significant maximum absorption peaks were obtained by the M06-2X functional: 226.47 nm (azo, TD), 204.58 nm (azo, TD), and 257.02 nm (hydrazo, TD). The experimental 216 nm wavelength would correspond to the absorption peaks calculated by the CIS and TD methods for the azo tautomer, and the 347 nm absorption peak to the hydrazo tautomer peaks calculated by the TD and ZINDO approaches.

For **4g** dye, the experimental UV-VIS spectrum showed the following maximum peaks: 220 nm (local maximum) for the carboxyethyl group in esters, 237 nm (local maximum) for chromophore ethyl 3-methyl-1*H*-pyrazole-4-carboxylate, and 312 nm for the azo group. The calculated maximum absorption peaks were: 207.65 nm (azo, CIS), 228.15 nm (hydrazo, CIS), 239.91 nm (azo, CIS), for the chlorine-carboxyethyl chromophore, 297.52 nm (azo, TD), 306.95 nm (azo, TD), and 325.87 nm (hydrazo, TD). The maximum absorption peaks obtained by the M06-2X functional were: 232.64 nm (azo, TD) and 235.23 nm (hydrazo, TD). The experimental peaks of 220 nm and 237 nm would correspond to those calculated by the CIS and TD methods for the azo and hydrazo tautomers, respectively, and that of 312 nm would correspond to that computed by the TD method for the hydrazo tautomer.

The **4h** dye experimental wavelengths were: 223 nm for the ester carboxyethyl chromophore, 293 nm with a shoulder at 305 nm for the ethyl 3-methyl-1*H*-pyrazole-4-carboxylate, and 359 nm for the azo group. The calculated absorption maxima were: 229.92 nm (azo, CIS), 262.39 nm (hydrazo, CIS), 291.69 nm (azo, TD), 252.72 nm (hydrazo, TD), 306.07 nm (azo, ZINDO), 270.76 nm (hydrazo, ZINDO), 265.41 nm (hydrazo, ZINDO), 368.13 nm (hydrazo, TD), 329.1nm (azo, ZINDO), and 365.64 nm (hydrazo, ZINDO). The absorption maxima 223.37 nm (azo, TD), 212.76 nm (azo, TD), and 257.62 nm (hydrazo, TD) were obtained by the M06-2X functional. The experimental maximum wavelength of 223 nm corresponds to those calculated by the CIS and TD approaches for the azo tautomer. The experimental peaks of 293 nm and 305 nm are in agreement with those simulated by the CIS and TD methods for the azo tautomer. The 359 nm experimental peak corresponds to the N(H)–N= group and was found in the simulated spectra by the TD and ZINDO methods of the hydrazo tautomer.

The theoretical CIS, TD, and ZINDO methods led to light absorption information, which was compared to the experimental UV-VIS spectra. More calculated maximum absorption peaks were present in the simulated spectra of the pyrazole azo dyes compared to the experimental ones (e.g., the peak corresponding to the chromophore ethyl-(3-methyl-1*H*-pyrazole-4)-carboxylate and to the –N(H)N= group in the hydrazo tautomers). These results indicate that both azo and hydrazo forms were present in the UV-VIS simulated spectra in ethanol. The TD method computed using the M06-2X functional gave similar absorption peaks to the experimental ones, except those corresponding to the –N=N– and –N(H)=N– groups, which were absent.

The maximum absorption bands in the range of 312–359 nm of the pyrazole azo dyes can be attributed to n → π* and/or π → π* electronic transitions of the azo chromophore. Most of the calculated maximum wavelengths of the azo tautomers using the CIS and ZINDO approaches were closer to the experimental absorption data and in fewer cases by the TD method using the B3LYP functional. Generally, in all pyrazole azo derivatives, the hydrazo tautomers absorb the light at higher wavelengths compared to the experiment due to their higher electron resonance ability and because of the longer resonance system. This happened due to the deprotonation of the active methylene group attached to the azo moiety by the solvent molecules, which increases the electron resonance and shifts the absorption peaks to the higher wavelengths. This fact is in agreement with the lower E_HOMO_-E_LUMO_ energy gap values due to the π electron extended conjugation in the hydrazo tautomers compared to the azo ones. The spectral shift is mostly the result of solute–solvent interactions, which better stabilize the π* antibonding orbital compared to the π bonding orbital in polar solvents.

**Scheme 3:**
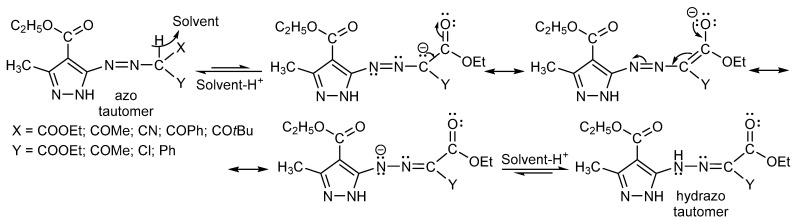
Azo-hydrazo tautomers and conjugation of the new pyrazole azo dyes.

The theoretical UV-VIS spectroscopic results confirmed the experimental ones. It could be concluded that the pyrazole azo dyes present the hydrazo tautomer as the prevalent form compared to the azo form, mainly in highly polarizable solvents, such as ethanol (Scheme 3).

### 3.6. Experimental Mass Spectra

The mass spectra for the new synthesized azo-pyrazole dyes were performed by two different techniques: electrospray ionization technique (ESI) for the compounds **4a**–**4d**, **4f**, and **4g** and electron impact ionization technique (EI) for the compounds **4f**, **4g**, and **4h**. The experimental spectra recorded by both techniques highlighted the corresponding molecular ion (peak) [M-H]^−^ in the ESI-MS technique and [M]^+·^ in the EI-MS one, respectively, confirming the molar mass of the synthesized compounds.

Moreover, in the case of dye **4g**, ethyl 2-(2-(4-(ethoxycarbonyl)-3-methyl-1H-pyrazol-5-yl)hydrazono)-2-chloroacetate, the molecular ion [M]^+·^ is represented by two peaks at 302 and 304 in a ratio of 3.2:1 corresponding to the two isotopes of the chlorine atom ^35^Cl and ^37^Cl in a relative ratio of 3:1. The next molecular ion in the spectrum of the mass also confirms the presence of the chlorine atom in its structure by the two peaks from 256 and 258 in the same ratio of 3.1:1. Thus, [M-46]^+^ corresponds to the elimination of a radical residue of C_2_H_5_OH, common in MS fragmentation of ethyl esters.

### 3.7. Color Analysis

As a general consideration, all dyes show decreasing values of luminosity L*, a moderate increase in red for the a* parameter, and an increase in yellow for the b* parameter when the dye concentration increases in the acrylic mixture. The electron-donating and electron-withdrawing groups influence the dye color [62,63].

**Figure 3:**
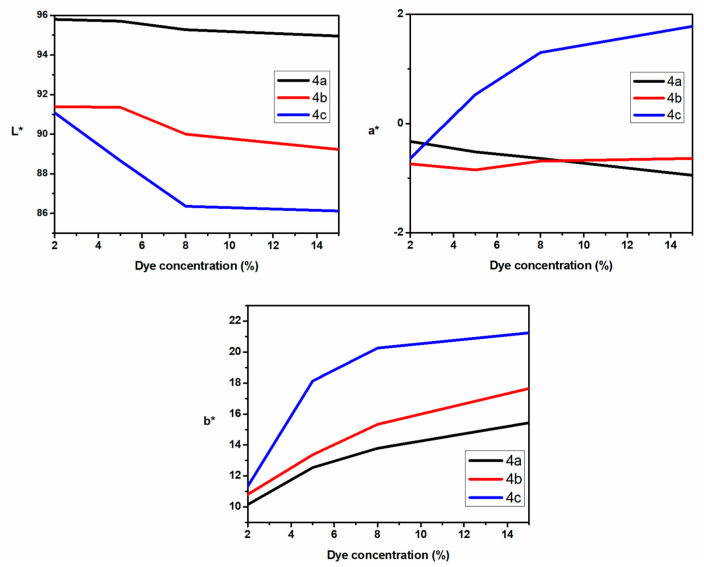
The dependence of CIEL*a*b* parameters on the dye concentration for dyes **4a**, **4b**, and **4c**.

The dependence of CIEL*a*b* parameters on the dye concentration in dyes **4a**, **4b**, and **4c** (Figure 3) reveal that the highest value for luminosity appears for dye **4a**. This dye presents two ester groups at the side chain. The a* parameter is in the green domain and the b* parameter is in the low value of the yellow domain. When one of the ester groups is changed with an acyl one (dye **4b**), the a* parameter presents similar values to those of dye **4a**, but the b* parameter has an increased value in the yellow field. When both side groups are acyl ones (dye **4c**), the shift of a* parameter to red is obvious, and the values of the b* parameter are higher in the yellow domain, as the dye concentration in the resins increases.

**Scheme 4:**
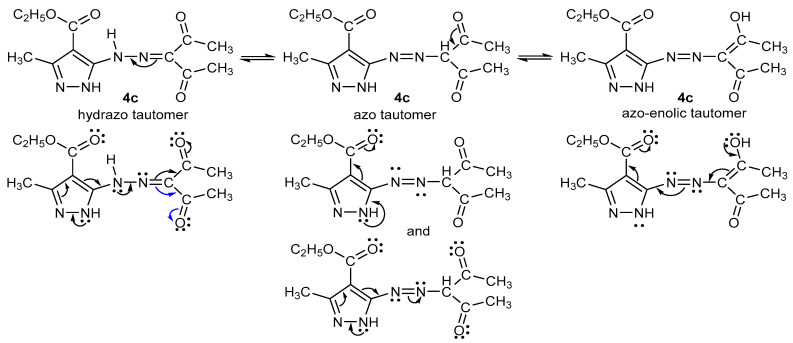
Azo-hydrazo tautomers and conjugation of dye **4c**.

This behavior in the color space may be explained by the fact that the acetyl groups, having stronger -E (electromer withdrawing) and -I (inductive withdrawing) effects than the ester groups, may favor both the hydrazo and azo-enolic tautomers, which presents the most extended conjugation. In dye **4c**, both acyl groups may be implicated in the conjugation (Scheme 4).

**Scheme 5:**
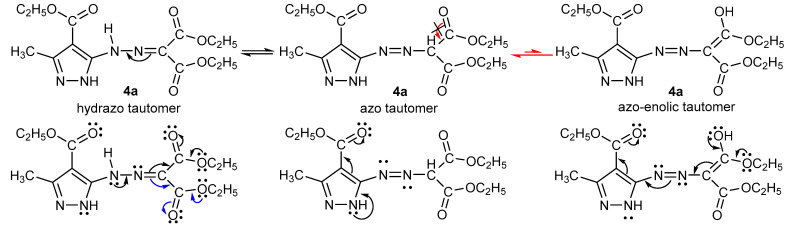
Azo-hydrazo tautomers and conjugation of dye **4a**.

In the case of dye **4a**, with two ester groups, the formation of an azo-enolic tautomer is less probable, due to the internal conjugation in the ester group, favoring the hydrazo tautomer (Scheme 5).

**Figure 4:**
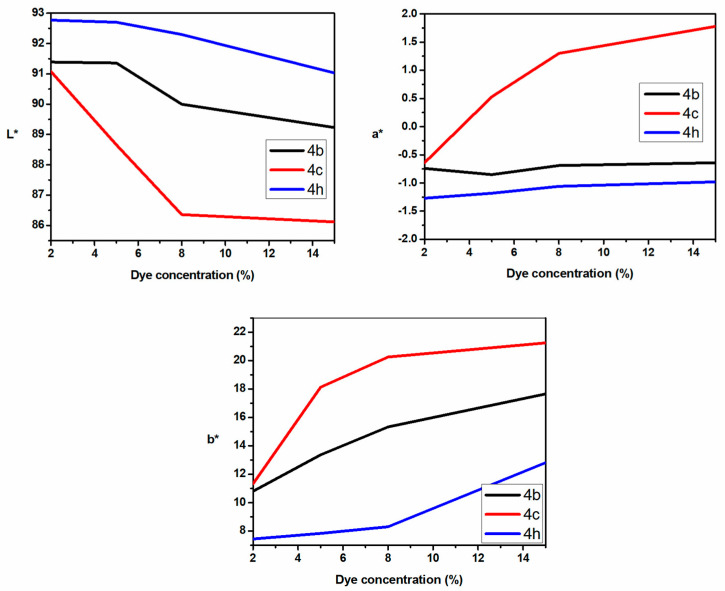
The dependence of CIEL*a*b* parameters on the dye concentration for dyes **4b**, **4c**, and **4h**.

The dependence of the CIEL*a*b* parameters on the dye concentration in dyes **4b**, **4c**, and **4h** (Figure 4), where at the side chain there is an acetyl group and other different ones, was compared.

The highest value for the luminosity appears for dye **4h**. The parameter a* is in the red domain for dye **4c**, and in the light green domain for dyes **4b** and **4h**. At the same time, the parameter b* has higher values in the yellow domain for dye **4c**, and lower values, also in the yellow domain, for dyes **4b** and **4h**, respectively.

This behavior in the color space may be explained by the fact that the phenyl group in dye **4h** is not involved in the extended conjugation of the azo group with active methylene.

**Figure 5:**
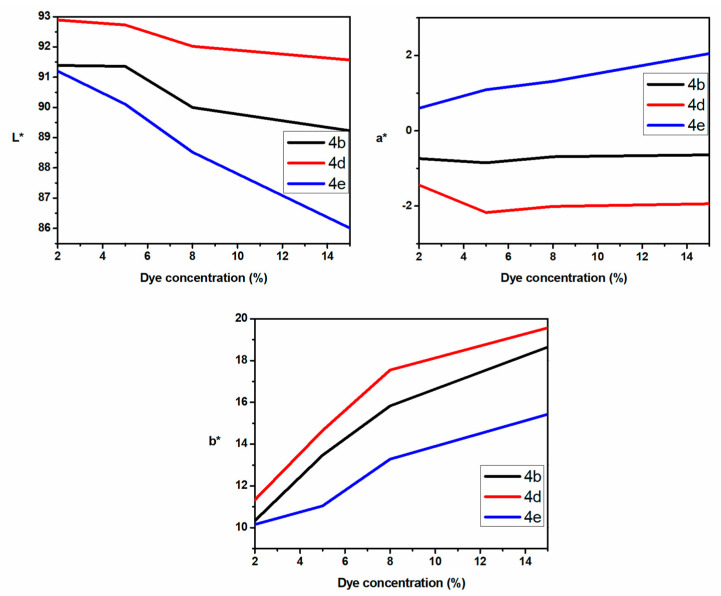
The dependence of CIEL*a*b* parameters on the dye concentration for dyes **4b**, **4d**, and **4e**.

Considering dyes **4b**, **4d**, and **4e**, which have an ester group in the side chain, the dependence of the CIEL*a*b* parameters on dye concentration is presented in Figure 5. Dye **4d** with the benzoyl group has the highest luminosity. The a* parameter is in the low red domain for dye **4e** and in the light green domain for the other two dyes. The b* parameter has higher values in the yellow domain for dye **4d** and lower values for dye **4e**.

These aspects may be explained considering that there may be an internal conjugation present in the benzoyl group that decreases the -E and -I effect of this group. The acetyl (dye **4b**) and pivaloyl (dye **4e**) groups, with +I effect of the methyl and *tert*-butyl, respectively, may favor the enolic tautomer, increasing the extended conjugation.

**Figure 6:**
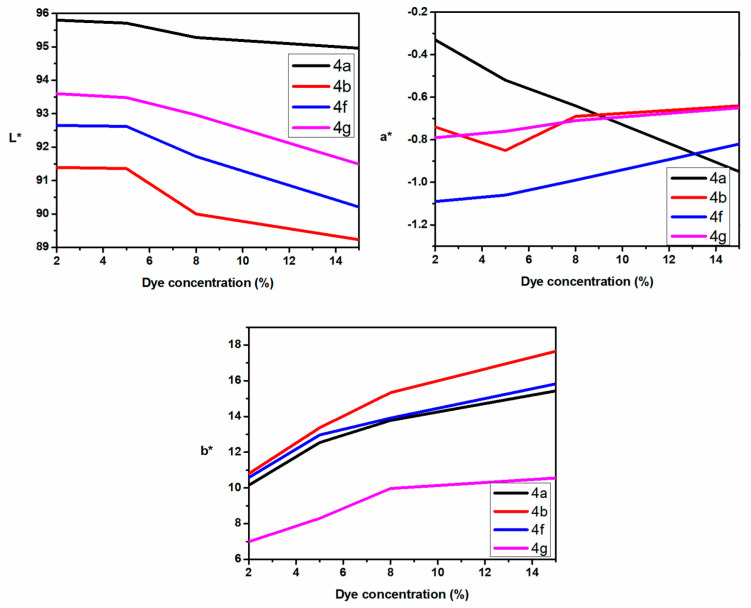
The dependence of CIEL*a*b* parameters on the dye concentration for dyes **4a**, **4b**, **4f**, and **4g**.

The dependence of the CIEL*a*b* parameters on the dye concentration of dyes **4a**, **4b**, **4f**, and **4g** (Figure 6) reveals that the highest luminosity appears for dye **4a** with two ester groups, followed by dye **4g** having one halogen atom, then dye **4f** which includes one cyan group, and dye **4b** with an acetyl group has the lowest luminosity. The other parameters, a* and b*, respectively, have approximately similar variations, except parameter a* of dye **4a**, but their values are close to one another, except for the b* parameter of dye **4g**.

**Figure 7:**
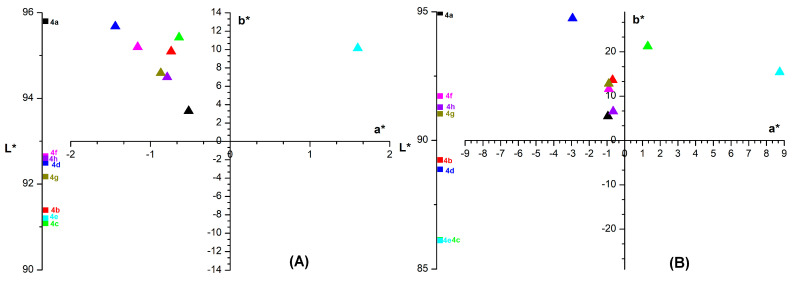
The extreme color parameters in the CIEL*a*b* system for all the new synthesized dyes at 2% (**A**) and 15% (**B**).

The Figure 7 presents the extreme color parameters in the CIEL*a*b* system for all the new synthesized dyes at 2% and 15%, that emphasize and confirm the results discussed.

**Scheme 6:**
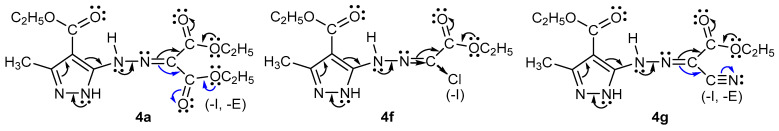
Conjugation for dyes **4a**, **4f**, and **4g**.

**Scheme 7:**
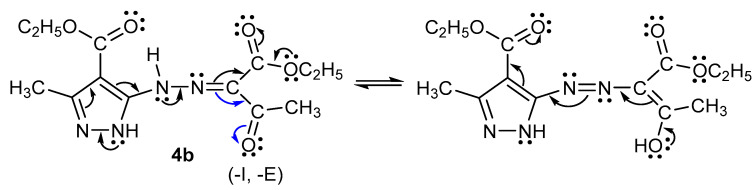
Conjugation and tautomers for dye **4b**.

This behavior in the color space may be explained for dyes **4a**, **4f**, and **4g**, considering the substituent polarity of the azomethylene group (Scheme 6) [64,65,66], and in dye **4b** the extended conjugation for the enolic tautomer appears, as presented above (Scheme 7).

The azo dye behavior in the color space indicates the prevalent hydrazo tautomer presence in the azo-hydrazo tautomerism, this fact being in accordance with the dye structural information obtained by the physico-chemical analyses presented above, and UV-VIS, ^1^H NMR, and ^13^C NMR spectra simulations using the DFT calculations.

### 3.8. Dye Toxicity Prediction

Several toxicity characteristics were calculated for the new azo dyes: the oral rodent acute toxicity (median lethal dose, LD_50_) and the corresponding toxicity classes, and other toxicities (Table S9, Supplementary Materials). The estimated LD_50_ values of the hydrazo tautomers of dyes **4c**, **4d**, **4f**, and **4g** correspond to the class III of toxicity (they would be toxic if swallowed). All the other hydrazo and azo tautomers would be harmful if swallowed (included in class IV of toxicity). The estimated dye hepatotoxicity, carcinogenicity, and mutagenicity effects indicate that they are not present for all dyes (for a probability of around 50%), except the azo and hydrazo tautomers of dye **4d**, which would have low hepatotoxicity (with probabilities of 50% and 51%, respectively) and low mutagenicity for the azo forms of dyes **4c** and **4h** (with probabilities of 51% and 50%, respectively). With a high probability for all the azo and hydrazo forms of all dyes, immunotoxicity and cytotoxicity effects are not present. The calculated BCF values of all azo and hydrazo tautomers of the pyrazole azo dyes are less than 1000; therefore, they have a low bioconcentration potential (https://www.epa.gov/sites/default/files/2015-05/documents/05.pdf, accessed 20 July 2022) for the aquatic organisms.

## 4. Conclusions

New eight pyrazole azo dyes were synthesized by the coupling reaction of the diazonium salt of ethyl 5-amino-3-methyl-1*H*-pyrazole-4-carboxylate with components having active methylene groups, in ethanol, in the presence of natrium acetate, with potential application in the varnish industry for light color paints. These compounds were characterized by physico-chemical analytical approaches. The experimental spectroscopic (UV-VIS, FT-IR, ^1^H NMR, ^13^C NMR) and spectrometric (MS) data explicitly support the proposed chemical structures of these new dyes. Theoretical calculations were performed to obtain structural information about these dyes in the absence of X-ray crystallographic data and to compare their UV-VIS, ^1^H NMR, and ^13^C NMR spectra outcomes with the experimental ones. Molecular mechanics and DFT calculations using the B3LYP functional were used for dye structure investigations in the gas phase. UV-VIS (at the B3LYP and M06-2X levels), ^1^H NMR, and ^13^C NMR spectra were simulated by several approaches, using the optimized azo and hydrazo dye tautomers of ground state obtained using DFT calculations. Weak intermolecular interactions were noticed (π-stacking and hydrogen bonding of the unconventional N−H···N type) in the dye azo tautomers and of type C−H···O in the hydrazo ones. The presence of H-bonds in all hydrazo dye tautomers of minimum energy indicates their better stability compared to the azo ones, this fact being in accordance with the calculated DFT minimum energy values and the azo dye behavior in the color space. The calculated ground-state global molecular reactivity parameters indicated that the hydrazo tautomers would be more easily involved in charge transfer reactions, having electron donor ability, contrary to the electron acceptor azo forms, which would be less reactive. The calculated maximum absorption wavelengths and chemical shifts were compared to the experimental spectra. The UV-VIS spectra were simulated using the CIS, TD, and ZINDO methods. The GIAO approach was employed to generate the chemical shifts of the ^1^H NMR and ^13^C NMR spectra. The theoretical ^1^H NMR and ^13^C NMR spectroscopic results were in agreement with the experimental ones, the pyrazole azo dyes being present as predominant hydrazo tautomers compared to the azo forms. Good predictions of the maximum absorption peaks were noticed when CIS, TD, and ZINDO methods were employed in UV-VIS spectra simulations. Both azo and hydrazo tautomers were present in the UV-VIS simulated spectra in ethanol solution. The electron donor hydrazo tautomers absorb the light at higher wavelengths compared to the experiment in the case of all these simulation approaches, being more polarizable due to their π electron extended conjugation. In order to emphasize the correlation between the substituents having methylene-active groups and the results obtained by the other analyses performed in this paper, the CIEL*a*b* color space approach was used. The measurements were performed for the newly synthesized dyes in powder and for their mixtures in different concentrations with a water-based acrylic resin that contained 65% titanium dioxide. The mixtures were applied on a cellulosic support. These analyses confirm the influence of the molecule conjugation on the dyes’ color. The estimated toxicity characteristics of the new azo dyes indicate a low bioaccumulative risk for aquatic organisms: they would be toxic or harmful if swallowed and they would have a very low probability risk to develop immunotoxicity and cytotoxicity effects and a low hepatotoxicity, carcinogenicity, and mutagenicity potential.

## Data Availability

All the experimental data obtained are presented, in the form of tables and/or figures, in the article and in the Supplementary Materials.

## Supplementary Materials

The following supporting information can be downloaded at: https://www.mdpi.com/article/10.3390/ma15165507/s1, Spectral data of the new azomethylene dyes; Table S1: Hydrogen bond properties of the azo and hydrazone energy optimized tautomers of the new azomethylene dyes; Table S2: Computed data obtained using molecular mechanics and quantum chemical (B3LYP) calculations for the energy minimized azomethylene dye tautomers; Table S3: Experimental (Exp) and calculated ^13^C and ^1^H chemical shift values of the azomethylene dyes; Table S4: Experimental UV-VIS spectra of the azomethylene dyes in ethanol; Table S5: Experimental and calculated UV-VIS absorption maxima (λ_max_, in parentheses) and their corresponding oscillator strength values of the azomethylene dyes obtained using the CIS, TD, and ZINDO methods; Table S6: Experimental MS spectra of the azomethylene dyes; Table S7: Experimental FT-IR spectra (in the range/domain of 4000–400 cm^−1^) of the azomethylene dyes; Table S8: Experimental FT-IR spectra (extended in the range/domain of 3600–2500 and 2500–400 cm^−1^) of the azomethylene dyes; Table S9: Calculated bioconcentration factor (BCF), oral rodent toxicity (LD50) with toxicity classes, and other endpoint toxicities (their probability is included in parentheses) for the new azomethylene dyes.
