# Supplementary material for: Experimental and Computational Study of Novel Pyrazole Azo Dyes as Colored Materials for Light Color Paints
Source: Materials (Basel). 2022 Aug 11;15(16):5507. doi: 10.3390/ma15165507 (PMC9410184; doi:10.3390/ma15165507)
Supplement: Supplementary file 1 [file materials-15-05507-s001.zip › materials-1784788-supplementary.pdf]

materials

Supplementary Material

Experimental and computational study of novel pyrazole azo dyes as colored materials for light color paints

Sabina Nitu [1,*], Marius Silviu Milea [1,*], Sorina Boran [1,*], Giannin Mosoarca [1,*], Alina D. Zamfir [2,*], Simona Popa [1,*] and Simona Funar-Timofei [3,*]

[1] Politehnica University of Timisoara, Faculty of Industrial Chemistry and Environmental Engineering, Bd. V. Parvan, No. 6, 300223, Timisoara, Romania;
[2] National Institute for Research and Development in Electrochemistry and Condensed Matter, Plautius Andronescu Str. 1, 300224, Timisoara, Romania;
[3] "Coriolan Dragulescu" Institute of Chemistry, Romanian Academy, Mihai Viteazul Blvd. No. 24, 300223, Timisoara.
* Correspondence: sabina.nitu@upt.ro (S.N.); marius.milea@upt.ro (M.S.M.); sorina.boran@upt.ro (S.B); giannin.mosoarca@upt.ro (G.M.); alina.zamfir@uav.ro (A.Z.); simona.popa@upt.ro (S.P.); timofei@acad-icht.tm.edu.ro (S.F-T); Tel.: +40-256-404-231 (S.N.); +40-256-404-232 (M.S.M.), +40-256-404-228 (S.B); +40-256-404-185 (G.M.); +40-256-404-212 (S.P.); +40-256-491-818 (S.F-T.).

Spectral data of the new azomethylene dyes

(4a)

diethyl 2-(2-(4-(ethoxycarbonyl)-3-methyl-1*H*-pyrazol-5-yl)hydrazono)malonate
Orange powder; yield 70.5%; mp 136-138°C
FT/IR (KBr disk, ν cm^{-1}): 3477w; 3353w; 3284w; 3193.5w; 3060.5w; 2986m; 2935w; 2905w; 2835w; 1704s; 1686.4s; 1667sh; 1533s; 1525sh; 1472w; 1450w; 1407m; 1380m; 1346w; 1307.5s; 1265m; 1201.4s; 1157m; 1130s; 1110m; 1056w; 1021m; 876.5w; 805m; 785m; 730w; 700w; 572w; 481w; 447w
UV-VIS (λ_{max}, nm; ethanol, ε , $mol^{-1}\cdot dm^3\cdot cm^{-1}$): 216 (19430); 239 (12330); 345 (20700)
^{1}H-RMN (δH ppm, $CDCl_3$, 200 MHz): 13.3 (1H, s, $\underline{HN}_{Pyr}$); 7.5 (1H, b.s, $\underline{NH}$-C_{5Pyr}); 4.42-4.25 (6H, m, C_{4Pyr}-$COO\underline{CH_2}CH_3$; $N{=}C(COO\underline{CH_2}CH_3)_2$); 2.45(3H, s, C_{3Pyr}-CH_3); 1.42-1.28 (9H, m, C_{4Pyr}-$COOCH_2\underline{CH_3}$; $N{=}C(COOCH_2\underline{CH_3})_2$);
^{13}C-RMN (δC ppm, $CDCl_3$, 50 MHz) 164.1 ($N{=}C$-$\underline{CO}OCH_2CH_3$); 163.5 ($N{=}C$-$\underline{CO}OCH_2CH_3$); 162.0 (C_{4Pyr}-$\underline{CO}OCH_2CH_3$) ; 150.4 (C_{5Pyr}); 145.8 (C_{3Pyr}); 122.9 ($N{=}\underline{C}(COOCH_2CH_3)_2$); 97.7 ($C_{4Pyr}$); 61.7 ($N{=}C(COO\underline{CH_2}$-$CH_3)_2$); 60,5 ($C_{4Pyr}$-$COO\underline{CH_2}CH_3$); 14.4($C_{4Pyr}$-$COOCH_2\underline{CH_3}$); 14.0 ($N{=}C(COOCH_2\underline{CH_3})_2$); 12.1 ($C_{3Pyr}$-$\underline{CH_3}$).
MS: m/z 339.285 (17.8) $[M\text{-}H]^-$; 293.204 (38.7); 193.914 (7.3); 158.789 (100); 112.442 (38.5); 68.049 (6.2); molecular formula $C_{14}H_{20}N_4O_6$ requires 340.14 $[M]^{+\cdot}$

(4b)

ethyl 2-(2-(4-(ethoxycarbonyl)-3-methyl-1H-pyrazol-5-yl)hydrazono)-3-oxobutanoate
Yellow powder; yield 75.5%; mp 152-154°C
FT/IR (KBr disk, ν cm^{-1}): 3290m; 3261m; 3196m; 2984w; 2935w; 2904w; 2872w; 1714m; 1679s; 1606m; 1557s; 1476m; 1444w; 1371m; 1317s; 1244m; 1192s; 1175m; 1138s; 1071m; 1053s; 1017w; 945m; 878w; 840w; 784w; 728w; 673w; 448w; 412w
UV-VIS (λ_{max}, nm; ethanol, ε , $mol^{-1}\cdot dm^3\cdot cm^{-1}$): 235 (11620); 322 (17120)
^{1}H-RMN (δH ppm, $CDCl_3$ / DMSO-d_6, 200 MHz): 10.86 (1H, s, $\underline{HN}_{Pyr}$); 7.07 (1H, b.s, $\underline{NH}$-C_{5Pyr}); 4.32 (4H, d q., *J* = 7Hz, C_{4Pyr}-$COO\underline{CH_2}CH_3$; $N{=}C$-$COO\underline{CH_2}CH_3$); 2.44 (3H, s, C_{3Pyr}-CH_3); 2.25 (3H, s, $CO\underline{CH_3}$); 1.41-1.32 (6H, d t., *J* = 6.8-7Hz, C_{4Pyr}-$COOCH_2\underline{CH_3}$; $N{=}C$-$COOCH_2\underline{CH_3}$);
^{13}C-RMN (δC ppm, $CDCl_3$ / DMSO-d_6, 50 MHz) 195.5 ($\underline{CO}CH_3$); 163.3 ($N{=}C$-$\underline{CO}OCH_2CH_3$); 162.5 (C_{4Pyr}-$\underline{CO}OCH_2CH_3$); 150.4 (C_{5Pyr}); 138.8 (C_{3Pyr}); 133.3 (NH-N=$\underline{C}$<);

93.13 (C_{4Pyr}); 61.2 ($N{=}C(COO\underline{CH}_2\text{-}CH_3)_2$); 59.84 ($C_{4Pyr}\text{-}COO\underline{CH}_2CH_3$); 25.44 ($CO\underline{CH}_3$); 14.48 ($C_{4Pyr}\text{-}COOCH_2\underline{CH}_3$); 14.19 ($N{=}C\text{-}COOCH_2\underline{CH}_3$); 14.03 ($C_{3Pyr}\text{-}\underline{CH}_3$).
MS: m/z 309.232 (34.8) $[M\text{-}H]^-$; 291.222 (71.7); 236.135 (69.5); 219.043 (35.3); 128.587 (100); 82.192 (4.2); molecular formula $C_{13}H_{18}N_4O_5$ requires 310.13 $[M]^{+\cdot}$

(4c)
3-(2-(4-(ethoxycarbonyl)-3-methyl-1H-pyrazol-5-yl)hydrazono)-2,4-pentandione
Yellow powder; yield 60.5%; mp 140-142°C
FT/IR (KBr disk, ν cm^{-1}): 3430sh,w; 3296s; 3191sh,w; 2982w; 2940w; 2872.5w; 2792w; 1692m-s; 1669s; 1603.5m-s; 1557s; 1474m; 1461w; 1445.4w; 1369sh,m; 1357m-i; 1317s; 1240m; 1186s; 1159m-s; 1127s; 1069w-m; 1039w; 1023w; 943m-s; 877w; 782m; 734w; 663.4w; 596w; 517w; 448w; 418.5w
UV-VIS (λ_{max}, nm; ethanol, ε , mol^{-1}·dm^3·cm^{-1}): 238 (5580); 260 (3720); 331 (7010)
^{1}H-RMN (δH ppm, DMSO-d_6, 200 MHz): 11.98 (1H, b.s, $\underline{HN}_{Pyr}$); 7.13 (1H, b.s, $\underline{NH}\text{-}C_{5Pyr}$); 4.27 (2H, q, *J* = 6.9Hz, $C_{4Pyr}\text{-}COO\underline{CH}_2CH_3$); 2.40 (3H, s, $CO\underline{CH}_3$); 2.35 (3H, s, $CO\underline{CH}_3$); 2.13 (3H, s, $C_{3Pyr}\text{-}CH_3$); 1.29 (3H, t, *J* = 6.9Hz, $C_{4Pyr}\text{-}COOCH_2\underline{CH}_3$);
^{13}C-RMN (δC ppm, DMSO-d_6, 50 MHz) 195.3 ($\underline{CO}CH_3$); 162.2 ($N{=}C\text{-}\underline{CO}OCH_2CH_3$); 149.5 ($C_{5Pyr}$); 138.9 ($C_{3Pyr}$); 138.12 ($NH\text{-}N{=}\underline{C}(COCH_3\underline{)_2}$); 92.65 ($C_{4Pyr}$); 59.17 ($C_{4Pyr}\text{-}COO\underline{CH}_2CH_3$); 26.57 ($CO\underline{CH}_3$); 24.37 ($CO\underline{CH}_3$); 14.48 ($C_{4Pyr}\text{-}COOCH_2\underline{CH}_3$); 13.79 ($C_{3Pyr}\text{-}\underline{CH}_3$).
MS: m/z 279.219 (13.2) $[M\text{-}H]^-$; 261.163 (100); 226.735 (15.8); 219.043 (49.8); 188.926 (15.2); 167.837 (21.0); 146.721 (3.4); 98.361 (38.1); 57.968 (8.7); molecular formula $C_{12}H_{16}N_4O_4$ requires 280.12 $[M]^{+\cdot}$

(4d)
ethyl 2-(2-(4-(ethoxycarbonyl)-3-methyl-1H-pyrazol-5-yl)hydrazono)-2-benzoylacetate
Yellow powder; yield 62.2%; mp 128-130°C
FT/IR (KBr disk, ν cm^{-1}): 3264m; 3270sh,w; 3191w; 2983w-m; 2935w; 2904w; 1720m-s; 1675s; 1605m; 1558s; 1474m; 1446w; 1394w; 1372m; 1323s; 1239m; 1203w; 1181m; 1137sh,m; 1115s; 1073w; 1024w; 949m; 911w; 841w; 785m; 759w; 713.5w-m; 695w; 676w; 409w
UV-VIS (λ_{max}, nm; ethanol, ε , mol^{-1}·dm^3·cm^{-1}): 236 (12720); 330 (14930)
^{1}H-RMN (δH ppm, $CDCl_3$, 200 MHz): 13.1 (1H, s, $\underline{HN}_{Pyr}$); 10.2 (1H, b.s, $\underline{NH}\text{-}C_{5Pyr}$); 7.8-7.3 (5H, m, $COC_6\underline{H}_5$); 4.34-4.22 (4H, m, $C_{4Pyr}\text{-}COO\underline{CH}_2CH_3$; $N{=}C\text{-}COO\underline{CH}_2CH_3$); 2.24 (3H, s, $C_{3Pyr}\text{-}CH_3$); 1.31-1.28 (6H, m, $C_{4Pyr}\text{-}COOCH_2\underline{CH}_3$; $N{=}C\text{-}COOCH_2\underline{CH}_3$);
^{13}C-RMN (δC ppm, $CDCl_3$, 50 MHz) 185.5 ($\underline{CO}C_6H_5$); 163.7 ($N{=}C\text{-}\underline{CO}OCH_2CH_3$); 162.8 ($C_{4Pyr}\text{-}\underline{CO}OCH_2CH_3$); 152.8 ($C_{5Pyr}$); 138.9 ($C_{3Pyr}$); 134.2 ($NH\text{-}N{=}\underline{C}<$); 130.1 ($C_{1Ph}$); 128.94 ($C_{4Ph}$); 128.34 ($C_{3Ph}$); 125.9 ($C_{2Ph}$); 93.4 ($C_{4Pyr}$); 62.1 ($N{=}C\text{-}COO\underline{CH}_2\text{-}CH_3$); 60.5 ($C_{4Pyr}\text{-}COO\underline{CH}_2CH_3$); 25.44 ($CO\underline{CH}_3$); 14.45 ($C_{4Pyr}\text{-}COOCH_2\underline{CH}_3$); 14.08 ($N{=}C\text{-}COOCH_2\underline{CH}_3$); 13.92 ($C_{3Pyr}\text{-}\underline{CH}_3$).
MS: m/z 371.334 (8.3) $[M\text{-}H]^-$; 325.273 (44.9); 267.134 (10.1); 226.053 (7.3); 190.936 (93.7); 144.669 (100); molecular formula $C_{18}H_{20}N_4O_5$ requires 372.14 $[M]^{+\cdot}$

(4e)
ethyl 2-(2-(4-(ethoxycarbonyl)-3-methyl-1H-pyrazol-5-yl)hydrazono)-2-pivaloylacetate
Yellowish powder; yield 59%; mp 102-104°C
FT/IR (KBr disk, ν cm^{-1}): 3449w-m; 3358w; 3284w; 3194.5w; 3059.5w; 2987w-m; 2880w; 2835w; 1704s; 1686.5s; 1666m-s; 1532s; 1525s; 1472w; 1451w; 1407w; 1381m; 1346w; 1307.5s; 1284w; 12645m; 1201.4s; 1157m-s; 1130s; 1110m-s; 1056m; 1022m; 878.4w; 805w-m; 785m; 730w; 700w; 481w; 447.4w
UV-VIS (λ_{max}, nm; ethanol, ε , mol^{-1}·dm^3·cm^{-1}): 212 (38560) max local; 230 (23550) max local; 285 (12840); 358 (22780)
^{1}H-RMN (δH ppm, DMSO-d_6, 200 MHz): 12.72(1H, s, HN_{Pyr}); 10.2(1H, b.s, $\underline{NH}\text{-}C_{5Pyr}$); 4.23-4.28(4H, m, $C_{4Pyr}\text{-}COO\underline{CH}_2CH_3$; $N{=}C\text{-}COO\underline{CH}_2CH_3$); 2.45(3H, s, $C_{3Pyr}\text{-}CH_3$); 1.28-1.34(6H, d. t., *J* = 6.9Hz, $C_{4Pyr}\text{-}COOCH_2\underline{CH_3;}$ $N{=}C\text{-}COOCH_2\underline{CH_3}$); 1.16(9H, s, $COC(\underline{CH}_3)_3$)

^{13}C-RMN (δC ppm, DMSO-d$_6$, 50 MHz): 210.5($\underline{CO}$-C(CH_3)$_3$); 163.8(C_{4Pyr}-$\underline{CO}OCH_2CH_3$); 162.7(N=C-$\underline{CO}OCH_2CH_3$); 149.6(C_{5Pyr}); 144.1(C_{3Pyr}); 133,8(N=$\underline{C}$<); 97.5(C_{4Pyr}); 61.8(N=C-$COO\underline{C}H_2CH_3$); 60.7(C_{4Pyr}-$COO\underline{C}H_2CH_3$); 41.2($COC\underline{C}$(CH_3)$_3$); 26.8(COC($\underline{C}H_3$)$_3$); 14.3(C_{4Pyr}-$COOCH_2\underline{C}H_3$); 14.1(N=C-$COOCH_2\underline{C}H_3$); 13.4(C_{3Pyr}-CH_3).

(4f)

ethyl 2-(2-(4-(ethoxycarbonyl)-3-methyl-1H-pyrazol-5-yl)hydrazono)-2-cyanoacetate
Yellow powder; yield 68.2%; mp 121-123°C (method 1 from ethyl cyanoacetate);
Yellow powder; yield 74.8%; mp 122-124°C (method 2 from ethyl 2-cyanoacetylacetate);
FT/IR (KBr disk, ν cm^{-1}): 3504w; 3363w; 3285m; 3153w; 3135w; 3077w; 2989w-m; 2931w; 2847w; 2209m; 1715.4s; 1600.6s; 1555s; 1538s; 1455w-m; 1403w; 1373w-m; 1309.4s; 1294s; 1233m; 1164.8m-s; 1144m-s; 1101s; 1054m; 1013.4m; 871m; 848w-m; 825w-m; 781m; 763m; 650w; 556.4w-m; 435w
UV-VIS (λ_{max}, nm; ethanol, ε , $mol^{-1}\cdot dm^3\cdot cm^{-1}$): 216 (37620) max local; 347 (30730)
^{1}H-RMN (δH ppm, DMSO-d$_6$, 200 MHz): 13.1 (1H, s, $\underline{HN}_{Pyr}$); 10.92 (1H, s, $\underline{NH}$-C_{5Pyr}); 4.38-4.23 (4H, m, C_{4Pyr}-$COO\underline{CH}_2CH_3$; N=C-$COO\underline{CH}_2CH_3$); 2.42 (3H, s, C_{3Pyr}-CH_3); 1.37-1.29 (6H, m, C_{4Pyr}-$COOCH_2\underline{CH}_3$; N=C-$COOCH_2\underline{CH}_3$);
^{13}C-RMN (δC ppm, DMSO-d$_6$, 50 MHz): 163.6 (C_{4Pyr}-$\underline{CO}OCH_2CH_3$); 159.9 (N=C-$\underline{CO}OCH_2CH_3$); 149.7 (C_{5Pyr}); 144.3 (C_{3Pyr}); 110.1 ($\underline{C}$≡N); 107.8 (NH-N=$\underline{C}$<); 97.9 (C_{4Pyr}); 61.8 (C_{4Pyr}-$COO\underline{CH}_2CH_3$); 60.3 (N=C-$COO\underline{CH}_2$-CH_3); 14.08 (C_{4Pyr}-$COOCH_2\underline{CH}_3$; N=C-$COOCH_2\underline{CH}_3$); 11.14 (C_{3Pyr}-$\underline{CH}_3$).
MS: m/z 292.187 (62.8) $[M\text{-}H]^-$; 246.102 (19.3); 188.88 (32.0); 132.583 (13.9); 111.425 (100); 65.02 (18); molecular formula $C_{12}H_{15}N_5O_4$ requires 293.11 $[M]^{+\cdot}$
MS: m/z 293 (52) $M^{+\cdot}$; 247 (100); 221 (54); 174 (34); 123 (40); 109 (15); 66 (37); 41 (33); 39 (93); molecular formula $C_{12}H_{15}N_5O_4$ requires 293.11 $M^{+\cdot}$

(4g)

ethyl 2-(2-(4-(ethoxycarbonyl)-3-methyl-1H-pyrazol-5-yl)hydrazono)-2-chloroacetate
Yellow powder; yield 72.3 %; mp 187-189°C (method 1 with ethyl 2-chloroacetylacetate)
FT/IR (KBr disk, ν cm^{-1}): 3509.8w-m; 3372w; 3283w-m; 3193.5w; 3136.7w; 3076w; 3022w; 2987w-m; 2918w-m; 2857w; 1730sh; 1713.4s; 1674s; 1572.7sh; 1548.6s; 1476m; 1454m-s; 1419m; 1389m; 1370m; 1344m; 1298s; 1281.5s; 1157s; 1137s; 1069m; 1022m; 867w; 806m; 787m; 752m; 656w; 582.4w; 513w; 487w
UV-VIS (λ_{max}, nm; ethanol, ε , $mol^{-1}\cdot dm^3\cdot cm^{-1}$): 220 (12440) max local; 237 (10120) max local; 312 (18860)
^{1}H-RMN (δH ppm, DMSO-d$_6$, 200 MHz): 12.82 (1H, s, $\underline{HN}_{Pyr}$); 9.93 (1H, s, $\underline{NH}$-C_{5Pyr}); 4.39 (2H, q, *J* = 7Hz, N=C-$COO\underline{CH}_2CH_3$); 4.26 (2H, q, *J* = 7Hz, C_{4Pyr}-$COO\underline{CH}_2CH_3$); 2.41 (3H, s, C_{3Pyr}-CH_3); 1.41-1.29 (6H, m, C_{4Pyr}-$COOCH_2\underline{CH}_3$; N=C-$COOCH_2\underline{CH}_3$); obs. There is no graph of the proton spectrum!
^{13}C-RMN (δC ppm, DMSO-d$_6$, 50 MHz) 164.01 (C_{4Pyr}-$\underline{CO}OCH_2CH_3$); 158.77 (N=C-$\underline{CO}OCH_2CH_3$); 150.68 (C_{5Pyr}); 144.03 (C_{3Pyr}); 117.53 (C_{4Pyr}); 96.31 (NH-N=$\underline{C}$<); 62.51 (C_{4Pyr}-$COO\underline{CH}_2CH_3$); 59.94 (N=C-$COO\underline{CH}_2$-CH_3); 14.14 (C_{4Pyr}-$COOCH_2\underline{CH}_3$); 14.05 (N=C-$COOCH_2\underline{CH}_3$); 11.36 (C_{3Pyr}-$\underline{CH}_3$).
MS: m/z 301.71 (4.3) $[M\text{-}H]^-$; 267.587 (100); 193.26 (15.2); 165.057 (25.4); 146.922 (12.6); molecular formula $C_{11}H_{15}ClN_4O_4$ requires 302.08 $M^{+\cdot}$
MS: m/z 304 (16); 302 (5) ratio 3.2:1; 258 (18); 256 (57); 221 (27); 149 (33); 123 (37); 67 (35); 66 (100); molecular formula $C_{11}H_{15}ClN_4O_4$ requires 302.08 $M^{+\cdot}$ m/z: 302.08 (100.0%), 304.08 (32.0%) ratio 3.125:1

(4h)
1-(2-(4-(ethoxycarbonyl)-3-methyl-1H-pyrazol-5-yl)hydrazono)-1-phenylacetone
Yellow-orange powder; yield 63.7%; mp 145-147°C
FT/IR (KBr disk, ν cm^{-1}): 3313s; 3186m; 3090m; 2980m; 2932m; 2904m; 2867w; 1889w; 1707m; 1671s; 1609s; 1580s; 1563s; 1321m; 1237m; 1189s; 1063m; 944m; 780w; 656w
UV-VIS (λ_{max}, nm; ethanol, ε , $mol^{-1}\cdot dm^{3}\cdot cm^{-1}$): 223 (34720); 293 (11530) cu umăr la 305 (10600); 359 (22310)
^{1}H-RMN (δH ppm, DMSO-d_6, 200 MHz): 11.2 (1H, $\underline{HN}_{Pyr}$); 7.83-7.86 (2H, d, *J* = 7.5Hz, $\underline{HC}_{2Ph}$); 7.40-7.59 (3H, m, $\underline{HC}_{3;4Ph}$); 4.26 (2H, q, *J* = 6.9Hz, C_{4Pyr}-COO$\underline{CH_2}$$CH_3$); 2.36 (3H, s, C_{3Pyr}-CH_3); 1.88 (3H, s, N=C-CO$\underline{CH_3}$); 1.30 (3H, t, *J* = 6.9Hz, C_{4Pyr}-COO$CH_2$$\underline{CH_3}$)
^{13}C-RMN (δC ppm, DMSO-d_6, 50 MHz) 200.5 (N=C(Ph)-$\underline{CO}$$CH_3$); 162.59 ($C_{4Pyr}$-$\underline{CO}OCH_2CH_3$); 149.35 ($C_{5Pyr}$); 142.36 ($C_{3Pyr}$); 139.92 (NH-N=$\underline{C}$<); 135.33 ($C_{1,Ph}$); 129.93-127.97 ($C_{2-4,Ph}$); 90.82 ($C_{4Pyr}$); 58.84 ($C_{4Pyr}$-COO$\underline{CH_2}$$CH_3$); 25.21 (N=C(Ph)-CO$\underline{CH_3}$); 14.54 ($C_{4Pyr}$-COO$CH_2$$\underline{CH_3}$); 13.93 ($C_{3Pyr}$-$\underline{CH_3}$).
MS: m/z 314 (16) $M^{+\cdot}$; 299 (18); 296 (56); 252 (16); 251 (42); 250 (71); 249 (76); 225 (22); 224 (27); 211 (46); 165 (23); 152 (15); 123 (17); 116 (78); 115 (100); 103; 90; 78; 65 (21); molecular formula $C_{16}H_{18}N_4O_3$ requires 314.14 $M^{+\cdot}$

Table S1. Hydrogen bond properties of the azo and hydrazone energy optimized tautomers of the new azomethylene dyes.*

**Azo dye tautomer**	**Hydrazone dye tautomer**
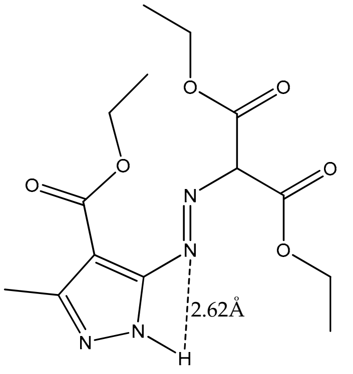 4a1<br>$d_{H-N...N}$=2.35Å<br>NHN angle= 63.39°<br>H-bond energy = -0.20 kcal/mol	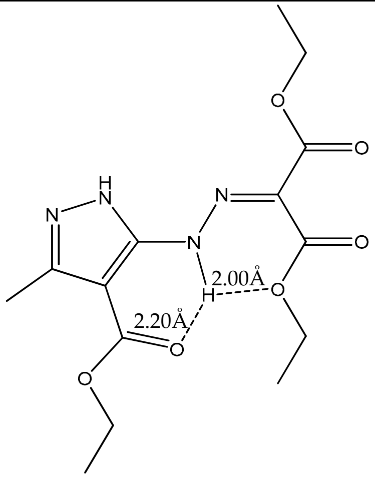 4a2<br>$d_{N(H)...O(=C)}$ = 2.86 Å<br>$d_{NH...O(C)}$ = 2.67 Å<br>NHO(=) angle= 121.1°<br>NHO(<) angle = 120.79°<br>three-centre (bifurcated donor)<br>H-bond energy = -0.45 kcal/mol

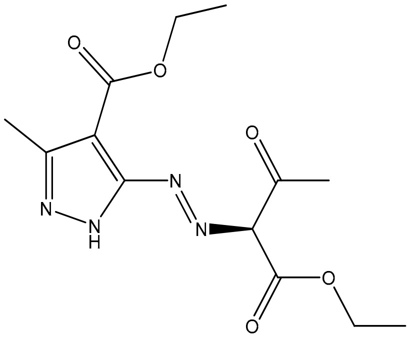<br>4b1	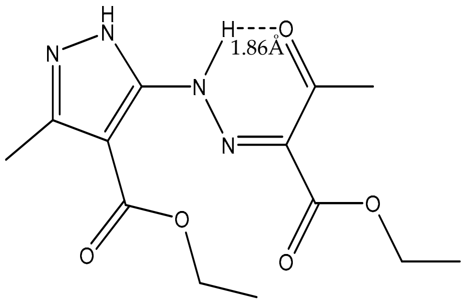<br><br>4b2<br>$d_{N(H)...O}$ = 2.58 Å<br>NHO angle= 124.03°<br>H-bond energy = -0.33 kcal/mol
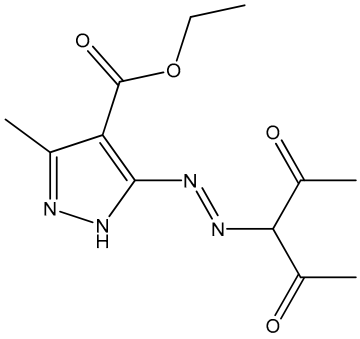<br>4c1	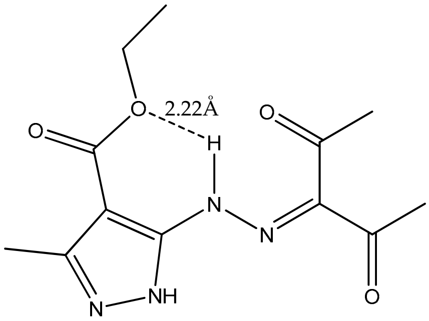<br><br>4c2<br>$d_{N(H)...O}$ = 2.83 Å<br>NHO angle=116.53°<br>H-bond energy = -0.19 kcal/mol
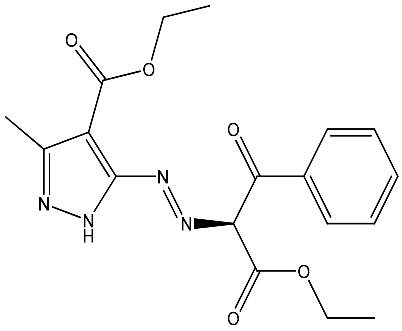<br>4d1	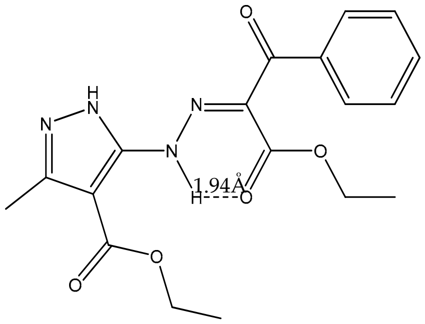<br><br>4d2<br>$d_{N(H)...O}$ = 2.66 Å<br>NHO angle = 124.87°<br>H-bond energy = -0.33 kcal/mol

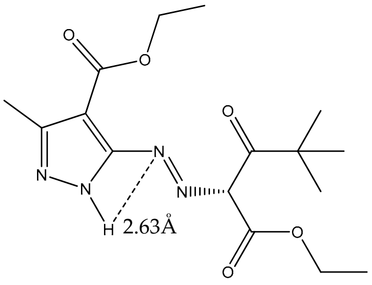<br>4e1<br>$d_{(H)N...N}$=2.35Å<br>NHN angle= 63.16°<br>H-bond energy = -0.21 kcal/mol	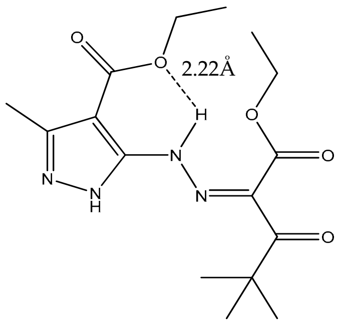<br>4e2<br>$d_{N(H)...O}$ = 2.87 Å<br>NHO angle = 119.79°<br>H-bond energy = -0.21 kcal/mol
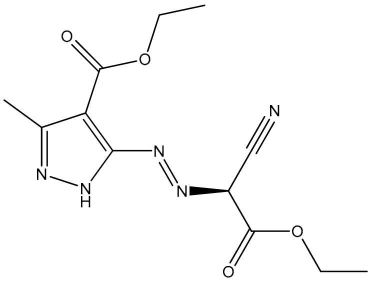<br>4f1	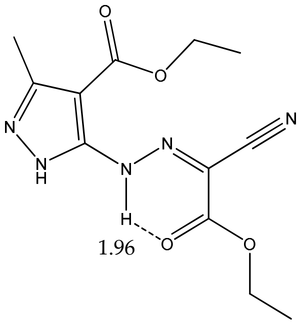<br>4f2<br>$d_{N(H)...O}$ = 2.67 Å<br>NHO angle = 123.91°<br>H-bond energy = -0.29 kcal/mol
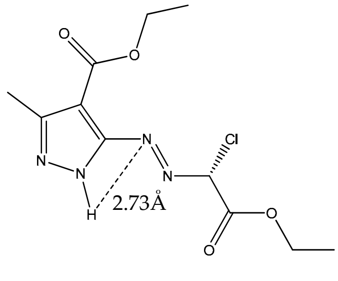<br>4g1<br>$d_{(H)N...N}$=2.42Å<br>NHN angle = 61.66°<br>H-bond energy = -0.25 kcal/mol	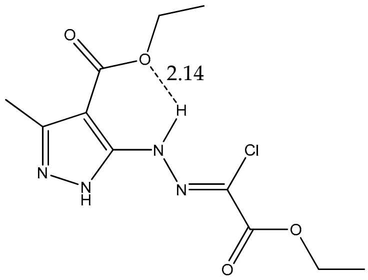<br>4g2<br>$d_{N(H)...O}$ = 2.80 Å<br>NHO angle = 120.69°<br>H-bond energy = -0.21 kcal/mol

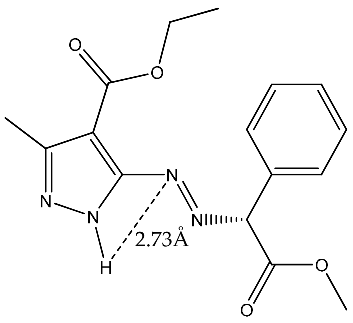<br><br>4h1<br>$d_{(H)N...N}$=2.43Å<br>NHN angle = 61.91°<br>H-bond energy = -0.24 kcal/mol	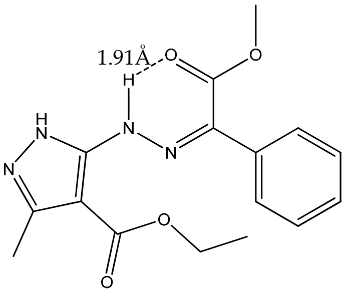<br><br>4h2<br>$d_{N(H)...O}$ = 2.63 Å<br>NHO angle = 124.89°<br>H-bond energy = -0.33 kcal/mol

* N – H .... A, (A= acceptor: O or N) and donor-acceptor ($d_{N(H)...O}$ and $d_{(H)N...N}$) hydrogen bond lengths (expressed in Ångström, Å); NHN and NHO-hydrogen bond angles; H-bond -hydrogen bond; H-bond energies were calculated using the CHARMM force field evaluated by the VEGA ZZ v. 3.0.1 software [1].

Table S2. Computed data obtained using molecular mechanics and quantum chemical (B3LYP) calculations for the energy minimized azomethylene dye tautomers.*

Dye	Tautomer	$E_{MMFF94s}$ (kcal/mol)	B3LYP									
			E(RB3LYP) (a.u.)	ZPE (Hartree/Particle)	Hcorr	Gcorr	E_{HOMO} (eV)	E_{LUMO} (eV)	η (eV)	μ (eV)	ω	σ
4a1	azo	16.88	-1216.180	0.348	0.376	0.287	-0.249	-0.090	-0.079	-0.169	0.181	12.639
4a2	hydrazone	89.19	-1216.214	0.350	0.377	0.290	-0.231	-0.082	-0.074	-0.156	0.165	13.480
4b1	azo	21.01	-1101.603	0.315	0.340	0.256	-0.252	-0.097	-0.077	-0.174	0.197	12.917
4b2	hydrazone	82.51	-1101.640	0.316	0.340	0.260	-0.234	-0.096	-0.069	-0.165	0.197	14.451
4c1	azo	24.27	-987.023	0.281	0.303	0.228	-0.249	-0.103	-0.073	-0.176	0.211	13.680
4c2	hydrazone	75.54	-987.062	0.282	0.305	0.231	-0.241	-0.108	-0.067	-0.174	0.227	14.988
4d1	azo	58.73	-1293.378	0.368	0.396	0.305	-0.248	-0.097	-0.076	-0.172	0.196	13.204
4d2	hydrazone	127.24	-1293.417	0.369	0.397	0.308	-0.231	-0.091	-0.070	-0.161	0.186	14.336
4e1	azo	35.43	-1219.570	0.399	0.428	0.337	-0.247	-0.092	-0.078	-0.169	0.185	12.877
4e2	hydrazone	97.4	-1219.604	0.400	0.429	0.338	-0.229	-0.086	-0.071	-0.157	0.173	14.008
4f1	azo	25.24	-1041.176	0.276	0.299	0.220	-0.268	-0.110	-0.079	-0.189	0.226	12.643
4f2	hydrazone	87.48	-1041.215	0.278	0.300	0.224	-0.239	-0.101	-0.069	-0.170	0.209	14.483
4g1	azo	42.03	-1408.542	0.269	0.291	0.213	-0.269	-0.114	-0.078	-0.191	0.236	12.881
4g2	hydrazone	92.46	-1408.572	0.269	0.292	0.215	-0.232	-0.080	-0.076	-0.156	0.159	13.148
4h1	azo	64.23	-1140.703	0.330	0.355	0.272	-0.252	-0.095	-0.079	-0.174	0.192	12.720
4h2	hydrazone	103.26	-1140.733	0.331	0.356	0.276	0.000	-0.083	-0.067	-0.150	0.168	14.863

* $E_{MMFF94s}$ –MMFF94s energy; E(RB3LYP) – calculated total energy (one Hartree = 627.509 kcal mol^{-1}); ZPE - unscaled zero-point correction; Hcorr - thermal correction to the enthalpy; Gcorr - thermal correction to the Gibbs free energy; E_{HOMO} and E_{LUMO} – highest occupied and lowest unoccupied molecular orbital energies, respectively; μ-electronic chemical potential; η-chemical hardness; ω-electrophilicity; σ-softness.

Table S3. Experimental (Exp) and calculated ^{13}C and ^{1}H chemical shift values of the azomethylene dyes.*

Dye	Tautomer	Exp ^{13}C chemical shifts for the Pyr-NH-N=C</ Pyr-N=N-CH< moiety (ppm)	Calculated ^{13}C chemical shifts for the Pyr-NH-N=C</ Pyr-N=N-CH< moiety (ppm)	Exp ^{1}H chemical shifts for the Pyr-NH-N=C< group (ppm)	Calculated ^{1}H chemical shifts for the Pyr-NH-N=C< group (ppm)	Exp ^{13}C chemical shifts for the C_{Pyr}-NH-N=C</ C_{Pyr}-N=N-CH< moiety (ppm)	Calculated ^{13}C chemical shifts for the C_{Pyr}-NH-N=C</ C_{Pyr}-N=N-CH< moiety (ppm)
4a1	hydrazone	122.9	131.61 ($CHCl_3$)**	7.5	11.16 ($CHCl_3$)**	150.4	154.54 ($CHCl_3$)**
4a2	azo		92.71 ($CHCl_3$)**				161.79 ($CHCl_3$)**
4b1	hydrazone	133.3	131.97 ($CHCl_3$)**<br>132.29 (DMSO)**	7.07	14.06 ($CHCl_3$)**<br>17.48 (DMSO)**	150.43	154.54 ($CHCl_3$)**<br>149.41 (DMSO)**
4b2	azo		105.47 ($CHCl_3$)**<br>105.33 (DMSO)**				161.72 ($CHCl_3$)**<br>156.17 (DMSO)**
4c1	hydrazone	138.12	139.43 (DMSO)**	7.13	14.27 (DMSO)**	149.5	150.79 (DMSO)**
4c2	azo		113.88 (DMSO)**				162.58 (DMSO)**
4d1	hydrazone	134.2	135.82 ($CHCl_3$)**	10.2	12.39 ($CHCl_3$)**	152.8	151.36 ($CHCl_3$)**
4d2	azo		95.95 ($CHCl_3$)**				162.77 ($CHCl_3$)**
4e1	hydrazone	133.8	137.11 (DMSO)**	10.2	12.41 (DMSO)**	149.6	154.75 (DMSO)**
4e2	azo		94.48 (DMSO)**				162.85 (DMSO)**
4f1	hydrazone	107.8	112.70 (DMSO)**	10.92	13.06 (DMSO)**	149.7	153.55 (DMSO)**
4f2	azo		74.35 (DMSO)**				158.99 (DMSO)**
4g1	hydrazone	117.53	127.31 (DMSO)**	9.93	8.88 (DMSO)**	150.68	150.94 (DMSO)**
4g2	azo		89.68 (DMSO)**				157.33 (DMSO)**
4h1	hydrazone	139.92	138.61 (DMSO)**	-	12.55 (DMSO)**	149.35	152.61 (DMSO)**
4h2	azo		95.66 (DMSO)**				156.96 (DMSO)**

* Pyr – pyrazole ring; the chemical shifts are calculated for the highlighted carbon and hydrogen atoms included in the mentioned groups. ** Solvent used (in paranthesis): DMSO-dimethylsulfoxide, CHCl3–chlorophorm.

Table S4. Experimental UV-VIS spectra of the azomethylene dyes in ethanol.

<table>
<tr>
<td>
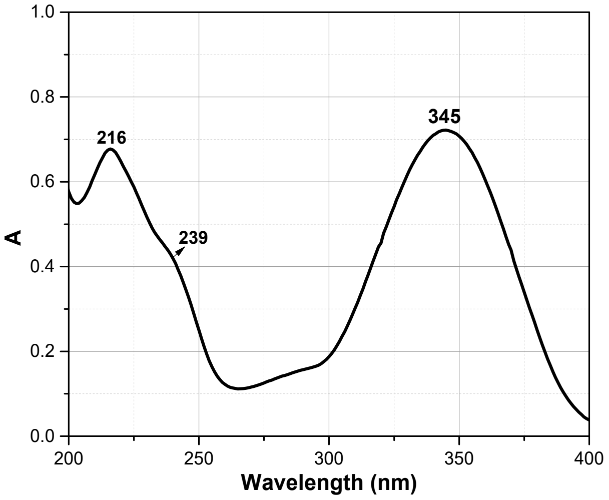

4a
λ_{max}, nm (ε, mol^{-1}·dm^3·cm^{-1}); ethanol:
216 (19430); 239 (12330); 345 (20700)
</td>
<td>
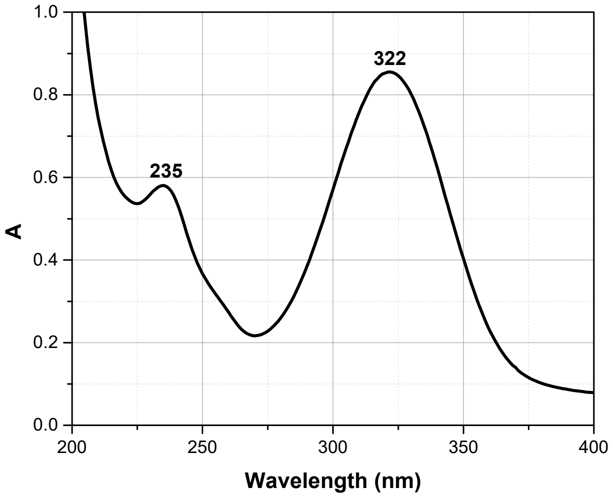

4b
λ_{max}, nm (ε, mol^{-1}·dm^3·cm^{-1}); ethanol:
235 (11620); 322 (17120)
</td>
</tr>
<tr>
<td>
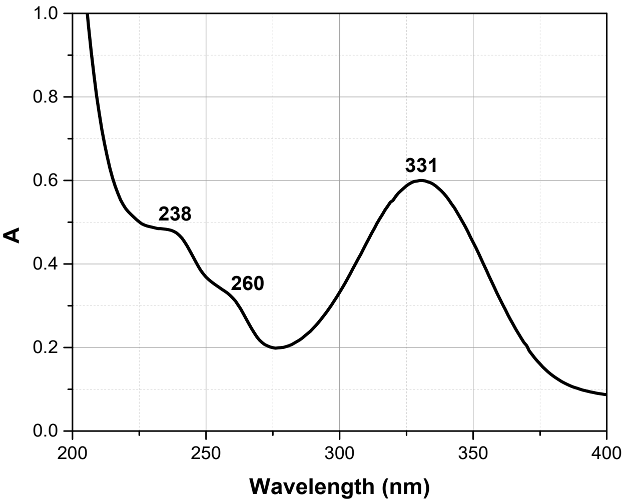

4c
λ_{max}, nm (ε, mol^{-1}·dm^3·cm^{-1}); ethanol:
238 (5580); 260 (3720); 331 (7010)
</td>
<td>
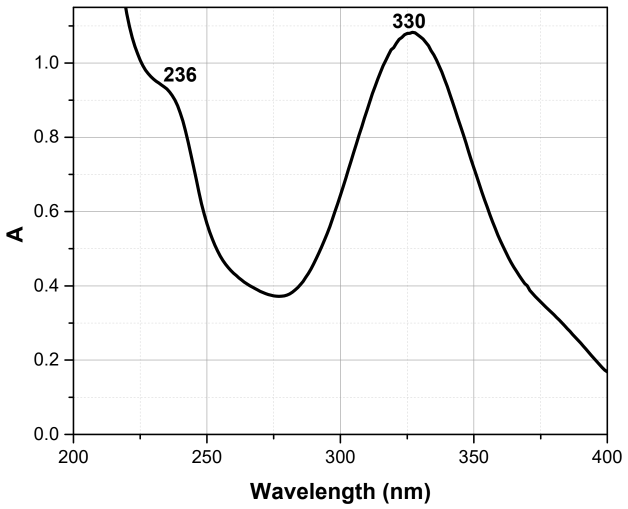

4d
λ_{max}, nm (ε, mol^{-1}·dm^3·cm^{-1}); ethanol:
236 (12720); 330 (14930)
</td>
</tr>
</table>

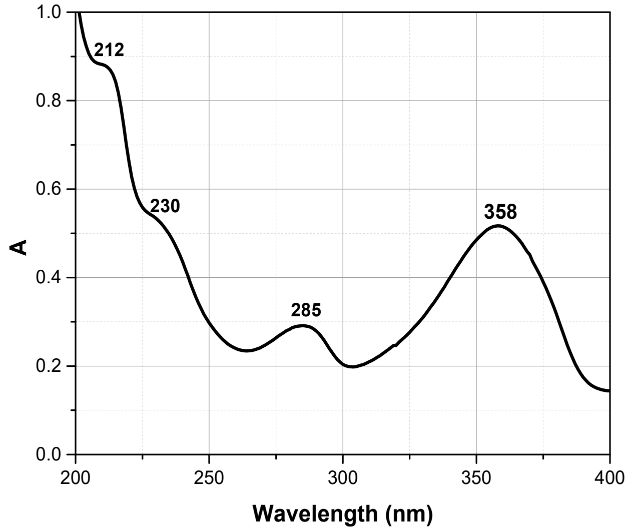

4e

λ_{max}, nm (ε, mol^{-1}·dm^{3}·cm^{-1}); ethanol:

212 (38560); 230 (23550); 285 (12840); 358 (22780)

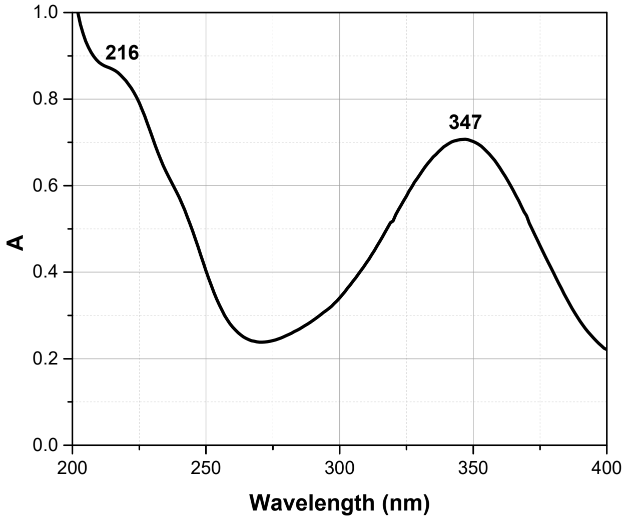

4f

λ_{max}, nm (ε, mol^{-1}·dm^{3}·cm^{-1}); ethanol:

216 (37620); 347 (30730)

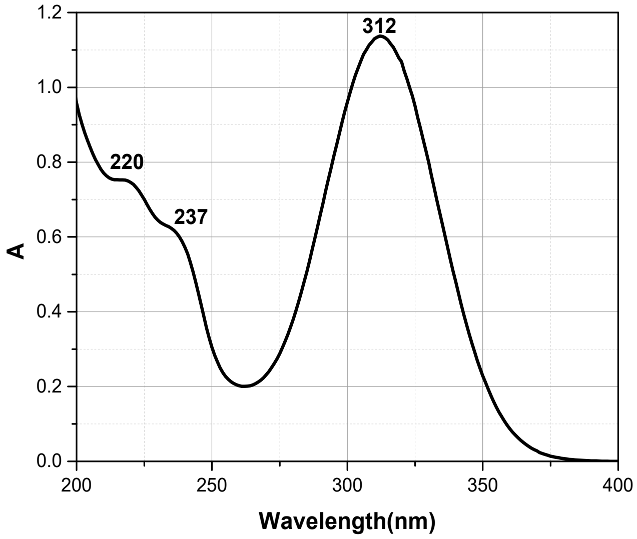

4g

λ_{max}, nm (ε, mol^{-1}·dm^{3}·cm^{-1}); ethanol:

220 (12440); 237 (10120); 312 (18860)

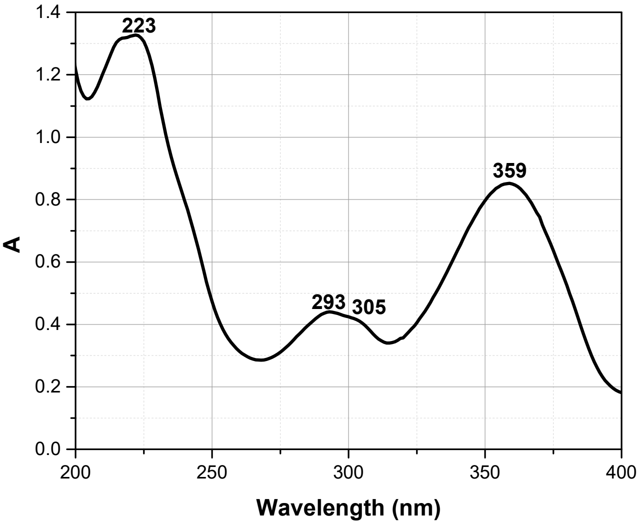

4h

λ_{max}, nm (ε, mol^{-1}·dm^{3}·cm^{-1}); ethanol:

223 (34720); 293 (11530); 305 (10600); 359 (22310)

Table S5. Experimental and calculated UV-VIS absorption maxima (λ_{max}, in parantheses) and their corresponding oscillator strength values of the azomethylene dyes obtained using the CIS, TD and ZINDO methods and B3LYP functional.

Dye	Tautomer	Method	Oscillator strength (λ_{max}, nm)								Experimental Wavelength (nm)
			S1*	S2*	S3*	S4*	S5*	S6*	S7*	S8*	
4a1	azo	CIS	0.004 (358.06)	0.488 (222.77)	0.298 (191.98)	0.002 (183.37)	0.002 (180.64)	0.003 (179.08)	0.023 (157.83)	0.492 (155.43)	216
4a2	hydrazo	CIS	0.982 (240.06)	0.025 (223.3)	0.002 (187.63)	0.030 (182.54)	0.443 (175.93)	0.001 (172.17)	0.033 (170.19)	0.391 (157.68)	239 (local maximum)
4a1	azo	TD	0.009 (423.65)	0.105 (308.4)	0.311 (293.48)	0.005 (286.16)	0.001 (269.21)	0.010 (257.18)	0.011 (243.46)	0.031 (238.93)	345
		TD**	0.002** (481.15)**	0.003** (283.45)**	0.030** (249.87)**	0.001** (245.83)**	0.355** (223.89)**	0.004** (211.90)**	0.016** (209.07)**	0.106** (203.56)**	
4a2	hydrazo	TD	0.556 (344.99)	0.001 (302.04)	0.021 (281.85)	0.000 (260.47)	0.002 (254.15)	0.207 (240.96)	0.012 (235.47)	0.020 (231.69)	
		TD**	0.001** (303.08)**	0.004** (276.07)**	0.0006** (271.71)**	0.718** (246.21)**	0.071** (226.41)**	0.019** (191.49)**	0.002** (187.57)**	0.022** (186.37)**	
4a1	azo	ZINDO	0.004 (460.32)	0.147 (334.21)	0.080 (325.73)	0.062 (318.22)	0.001 (312.93)	0.129 (288.72)	0.016 (245.32)	0.055 (234.63)	
4a2	hydrazo	ZINDO	0.002 (364.45)	0.033 (346.73)	0.493 (336.04)	0.001 (320.9)	0.027 (294.99)	0.088 (267.39)	0.010 (253.98)	0.404 (235.78)	
4b1	azo	CIS	0.003 (363.04)	0.099 (235.11)	0.459 (226.16)	0.311 (194.76)	0.001 (183.59)	0.001 (183.26)	0.032 (158.5)	0.584 (155.23)	235
4b2	hydrazo	CIS	0.003 (269.09)	0.933 (258.87)	0.002 (209.56)	0.000 (186.95)	0.295 (178.86)	0.000 (173.88)	0.079 (172.3)	0.323 (164.24)	322
4b1	azo	TD	0.004 (429.4)	0.325 (326.05)	0.049 (303.23)	0.022 (288.55)	0.105 (281.53)	0.011 (273.27)	0.007 (259.93)	0.008 (244.12)	
		TD**	0.0016** (488.16)**	0.0016** (321.73)**	0.0029** (287.58)**	0.0296** (253.46)**	0.4195** (229.79)**	0.0153** (209.74)**	0.0615** (207.58)**	0.0472** (202.69)**	
4b2	hydrazo	TD	0.001 (377.58)	0.594 (361.9)	0.029 (301.1)	0.000 (291.58)	0.000 (279.41)	0.047 (246.8)	0.000 (242.82)	0.183 (239.35)	
		TD**	0.0004** (406.47)**	0.0008** (295.48)**	0.0004** (273.82)**	0.7239** (259.72)**	0.0018** (240.24)**	0.0155** (201.77)**	0.0007** (198.11)**	0.000** (195.61)**	

4b1	azo	ZINDO	0.002 (470.57)	0.003 (368.89)	0.210 (337.06)	0.083 (328.29)	0.035 (321.06)	0.133 (293.34)	0.016 (255.15)	0.022 (244.2)	
4b2	hydrazo	ZINDO	0.001 (498.56)	0.503 (366.86)	0.001 (355.03)	0.001 (327.64)	0.004 (318.48)	0.102 (265.3)	0.003 (263.16)	0.253 (242.14)	
4c1	azo	CIS	0.004 (368.04)	0.128 (238.32)	0.019 (234.15)	0.389 (223.96)	0.314 (194)	0.001 (183.08)	0.105 (159.5)	0.491 (156.22)	238 (local maximum)
4c2	hydrazo	CIS	0.012 (273.32)	0.961 (263.21)	0.001 (240.49)	0.002 (205.29)	0.261 (178.66)	0.014 (176.12)	0.054 (172.3)	0.383 (164.2)	260 (local maximum)
4c1	azo	TD	0.017 (436.84)	0.259 (342.93)	0.032 (306.3)	0.030 (303.95)	0.006 (290.61)	0.136 (282.12)	0.012 (272.65)	0.005 (268.03)	331
		TD**	0.0026** (496.76)**	0.0025** (326.19)**	0.0023** (319.29)**	0.0026** (286.96)**	0.4136** (233.49)**	0.0149** (211.20)**	0.0273** (208.24)**	0.0449** (204.70)**	
4c2	hydrazo	TD	0.001 (405)	0.575 (365.55)	0.001 (338.97)	0.030 (304.97)	0.002 (275.59)	0.000 (265.47)	0.158 (248.11)	0.016 (245.49)	
		TD**	0.0003** (418.83)**	0.000** (367.40)**	0.0032** (271.86)**	0.7118** (263.68)**	0.0041** (246.23)**	0.0003** (208.74)**	0.0053** (193.04)**	0.0137** (191.14)**	
4c1	azo	ZINDO	0.004 (472.96)	0.009 (380.49)	0.002 (372.15)	0.156 (335.17)	0.147 (326.2)	0.133 (291.99)	0.017 (259.88)	0.014 (246.26)	
4c2	hydrazo	ZINDO	0.001 (500.95)	0.000 (431.68)	0.540 (376.48)	0.001 (327.11)	0.007 (317.82)	0.055 (269.46)	0.111 (263.21)	0.126 (241.69)	
4d1	azo	CIS	0.003 (362.83)	0.044 (247.81)	0.392 (226.33)	0.139 (220.34)	0.288 (217.35)	0.295 (193.15)	0.001 (183.58)	0.008 (178.27)	236 (local maximum)
4d2	hydrazo	CIS	0.592 (248.71)	0.319 (246.77)	0.049 (223.03)	0.257 (215.54)	0.080 (214.94)	0.151 (182.68)	0.285 (176.36)	0.405 (175.86)	330
4d1	azo	TD	0.006 (438.73)	0.112 (346.35)	0.049 (317.72)	0.008 (314.77)	0.107 (307.84)	0.020 (297.15)	0.026 (296.79)	0.169 (293.06)	
		TD**	0.0021** (488.37)**	0.0019** (368.78)**	0.0028** (285.99)**	0.0013** (245.02)**	0.3605** (230.78)**	0.019** (214.45)**	0.0194** (212.75)**	0.0229** (210.61)**	
4d2	hydrazo	TD	0.534 (358.11)	0.012 (344.67)	0.070 (307.38)	0.005 (299.46)	0.003 (292.94)	0.064 (290.09)	0.015 (289.34)	0.009 (267.15)	
		TD**	0.0006** (389.08)**	0.0066** (312.83)**	0.0005** (271.37)**	0.7190** (252.52)**	0.0267** (227.72)**	0.0463** (208.89)**	0.0013** (204.55)**	0.0088** (199.98)**	
4d1	azo	ZINDO	0.003 (462.83)	0.005 (419.87)	0.111 (334.69)	0.164 (326.09)	0.001 (312.59)	0.117 (288.27)	0.027 (278.44)	0.211 (265.5)	

4d2	hydrazo	ZINDO	0.003 (425.52)	0.023 (392.2)	0.473 (354.61)	0.001 (324.84)	0.028 (297.9)	0.024 (277.83)	0.234 (266.21)	0.111 (265.14)	
4e1	azo	CIS	0.003 (360.64)	0.033 (243.6)	0.446 (221.51)	0.300 (191.02)	0.001 (182.47)	0.003 (178.35)	0.022 (157.16)	0.460 (156.6)	212 (local maximum)
4e2	hydrazo	CIS	0.001 (251.81)	0.886 (245.84)	0.055 (220.41)	0.007 (180.54)	0.444 (176.29)	0.000 (172.73)	0.040 (170.45)	0.287 (159.42)	230 (local maximum)
4e1	azo	TD	0.008 (429.92)	0.114 (344.91)	0.049 (309.6)	0.230 (292.1)	0.017 (289.19)	0.001 (273.44)	0.013 (264.67)	0.014 (245.3)	285
		TD**	0.0028** (484.70)**	0.003** (329.60)**	0.0029** (283.46)**	0.0032** (244.74)**	0.3374** (231.52)**	0.0321** (213.18)**	0.0137** (211.24)**	0.0252** (207.81)**	358
4e2	hydrazo	TD	0.004 (357.49)	0.592 (351.97)	0.014 (285.37)	0.012 (284.32)	0.001 (264.41)	0.029 (260.41)	0.037 (257.37)	0.156 (239.08)	
		TD**	0.0003** (368.36)**	0.0114** (307.64)**	0.0004** (270.67)**	0.7370** (249.82)**	0.0398** (226.19)**	0.0014** (201.53)**	0.0019** (189.38)**	0.0145** (187.32)**	
4e1	azo	ZINDO	0.004 (458.55)	0.005 (385.97)	0.103 (332.56)	0.166 (323.07)	0.001 (315.66)	0.119 (286.02)	0.010 (249.05)	0.031 (243.84)	
4e2	hydrazo	ZINDO	0.002 (412.81)	0.025 (390.79)	0.446 (349.31)	0.001 (326.03)	0.014 (297.74)	0.092 (268.37)	0.011 (255.7)	0.274 (239.04)	
4f1	azo	CIS	0.001 (355.02)	0.683 (234.9)	0.213 (205.31)	0.001 (186.37)	0.003 (177.99)	0.003 (169.43)	0.011 (165.43)	0.029 (158.8)	216 (local maximum)
4f2	hydrazo	CIS	1.021 (259.71)	0.003 (227.73)	0.003 (181.48)	0.208 (180.55)	0.001 (178.4)	0.000 (173.57)	0.013 (173.53)	0.000 (173.53)	347
4f1	azo	TD	0.001 (429.85)	0.067 (321.21)	0.003 (316.75)	0.531 (294.92)	0.019 (275.88)	0.028 (286.02)	0.002 (251.25)	0.000 (238.87)	
		TD**	0.0003** (476.63)**	0.0005** (303.12)**	0.0031** (241.17)**	0.5049** (226.47)**	0.0184** (216.19)**	0.0372** (215.68)**	0.0945** (211.46)**	0.1364** (204.58)**	
4f2	hydrazo	TD	0.656 (359.97)	0.028 (303.49)	0.000 (296.39)	0.000 (278.99)	0.175 (240.67)	0.000 (237.15)	0.022 (236.11)	0.110 (226.64)	
		TD**	0.0003** (313.98)**	0.0005** (275.43)**	0.8270** (257.02)**	0.0043** (228.01)**	0.0002** ()200.71**	0.0347** (198.82)**	0.0256** (192.14)**	0.0507** (185.89)**	
4f1	azo	ZINDO	0.001 (459.21)	0.003 (338.83)	0.434 (329.99)	0.002 (317.16)	0.094 (310.54)	0.009 (246.77)	0.003 (235.97)	0.175 (228.9)	
4f2	hydrazo	ZINDO	0.000 (399.15)	0.506 (366.71)	0.001 (326.54)	0.009 (297.45)	0.100 (266.84)	0.012 (263.33)	0.236 (242.94)	0.000 (239.96)	

4g1	azo	CIS	0.000<br>(359.27)	0.758<br>(239.91)	0.228<br>(207.65)	0.005<br>(189.18)	0.001<br>(185.17)	0.014<br>(167.81)	0.002<br>(163.87)	0.018<br>(162.09)	220<br>(local maximum)
4g2	hydrazo	CIS	1.081<br>(228.15)	0.000<br>(192.31)	0.005<br>(190.74)	0.001<br>(175.78)	0.294<br>(173.62)	0.033<br>(168.12)	0.003<br>(164.19)	0.564<br>(156.14)	237<br>(local maximum)
4g1	azo	TD	0.000<br>(443.98)	0.090<br>(331.02)	0.001<br>(310.43)	0.385<br>(306.95)	0.128<br>(297.52)	0.001<br>(267.87)	0.053<br>(264.07)	0.001<br>(254.34)	312
		TD**	0.000**<br>(500.97)**	0.0004**<br>(305.50)**	0.0194**<br>(270.83)**	0.5865**<br>(232.64)**	0.0250**<br>(223.69)**	0.0291**<br>(219.40)**	0.0711**<br>(218.22)**	0.0331**<br>(217.45)**	
4g2	hydrazo	TD	0.653<br>(325.87)	0.028<br>(267.85)	0.000<br>(264.61)	0.000<br>(245.62)	0.095<br>(236.64)	0.000<br>(224.33)	0.000<br>(222.28)	0.091<br>(220.07)	
		TD**	0.0004**<br>(287.54)**	0.0003**<br>(271.31)**	0.8588**<br>(235.23)**	0.0053**<br>(206.42)**	0.004**<br>(181.50)**	0.0349**<br>(179.87)**	0.0081**<br>(179.43)**	0.0851**<br>(176.82)**	
4h1	azo	CIS	0.000<br>(359.12)	0.852<br>(229.92)	0.005<br>(204.18)	0.024<br>(201.04)	0.211<br>(198.37)	0.001<br>(183.15)	0.002<br>(182.24)	0.291<br>(176.79)	223
4h2	hydrazo	CIS	1.027<br>(262.39)	0.007<br>(223.51)	0.009<br>(208.69)	0.069<br>(203.1)	0.015<br>(184.28)	0.124<br>(177.69)	0.003<br>(174.92)	0.520<br>(172.12)	293 with shoulder<br>at 305
4h1	azo	TD	0.001<br>(434.08)	0.076<br>(330.75)	0.005<br>(318.37)	0.127<br>(308.12)	0.422<br>(291.69)	0.018<br>(287.58)	0.022<br>(265.15)	0.002<br>(251.05)	359
		TD**	0001**<br>(483.64)**	0.0004**<br>(294.43)**	0.0118**<br>(251.23)**	0.4526**<br>(223.37)**	0.3047**<br>(212.76)**	0.0075**<br>(207.19)**	0.0583**<br>(206.48)**	0.0479**<br>(202.50)**	
4h2	hydrazo	TD	0.630<br>(368.13)	0.002<br>(296.44)	0.022<br>(287.56)	0.015<br>(279.72)	0.008<br>(278.6)	0.264<br>(252.72)	0.001<br>(252.45)	0.012<br>(248.58)	
		TD**	0.0038**<br>(311.21)**	0.0009**<br>(270.50)**	0.9073**<br>(257.62)**	0.0050**<br>(225.69)**	0.0008**<br>(199.95)**	0.0178**<br>(187.49)**	0.1079**<br>(184.93)**	0.0066**<br>(183.72)**	
4h1	azo	ZINDO	0.001<br>(457.85)	0.003<br>(336.77)	0.535<br>(329.1)	0.001<br>(315.84)	0.123<br>(306.07)	0.025<br>(289.67)	0.000<br>(271.62)	0.009<br>(255.15)	
4h2	hydrazo	ZINDO	0.016<br>(395.31)	0.538<br>(365.64)	0.001<br>(323.32)	0.014<br>(293.69)	0.001<br>(276.46)	0.136<br>(270.76)	0.108<br>(265.41)	0.020<br>(255.43)	

* Excited states. ** TD results obtained using the M06-2X functional.

Table S6. Experimental MS spectra of the azomethylene dyes.

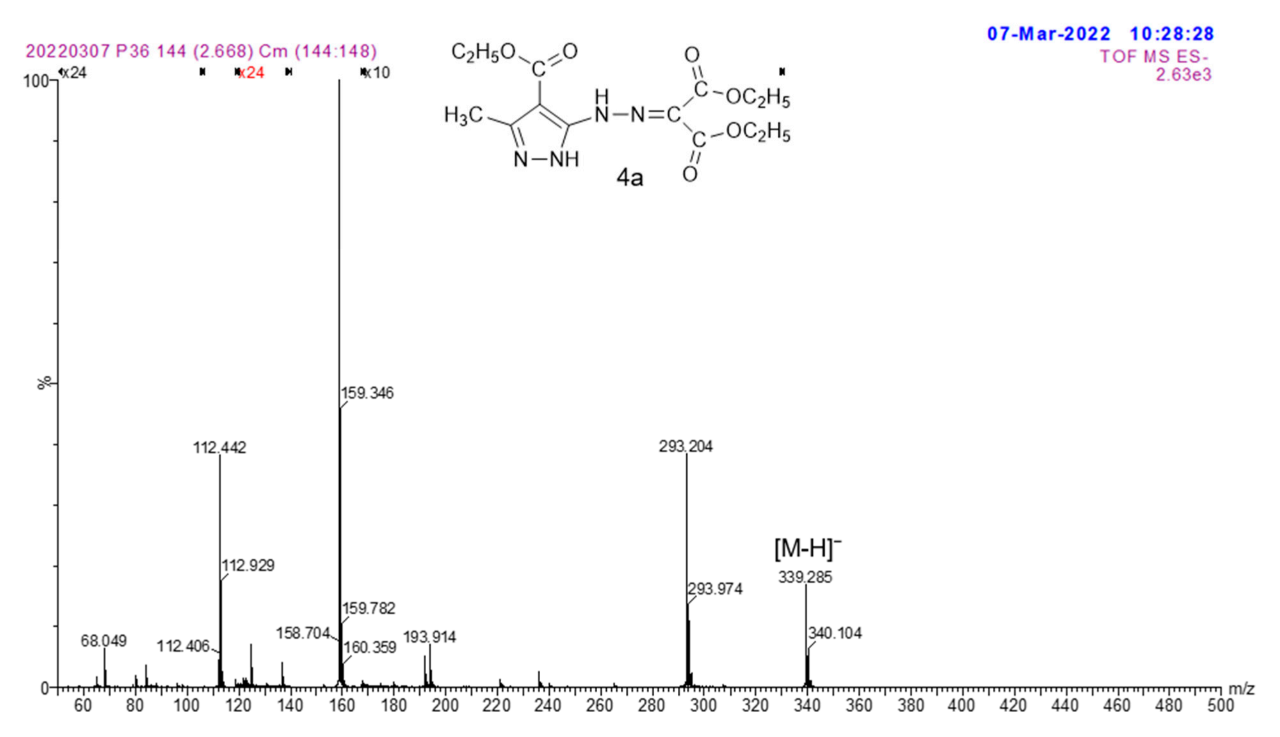

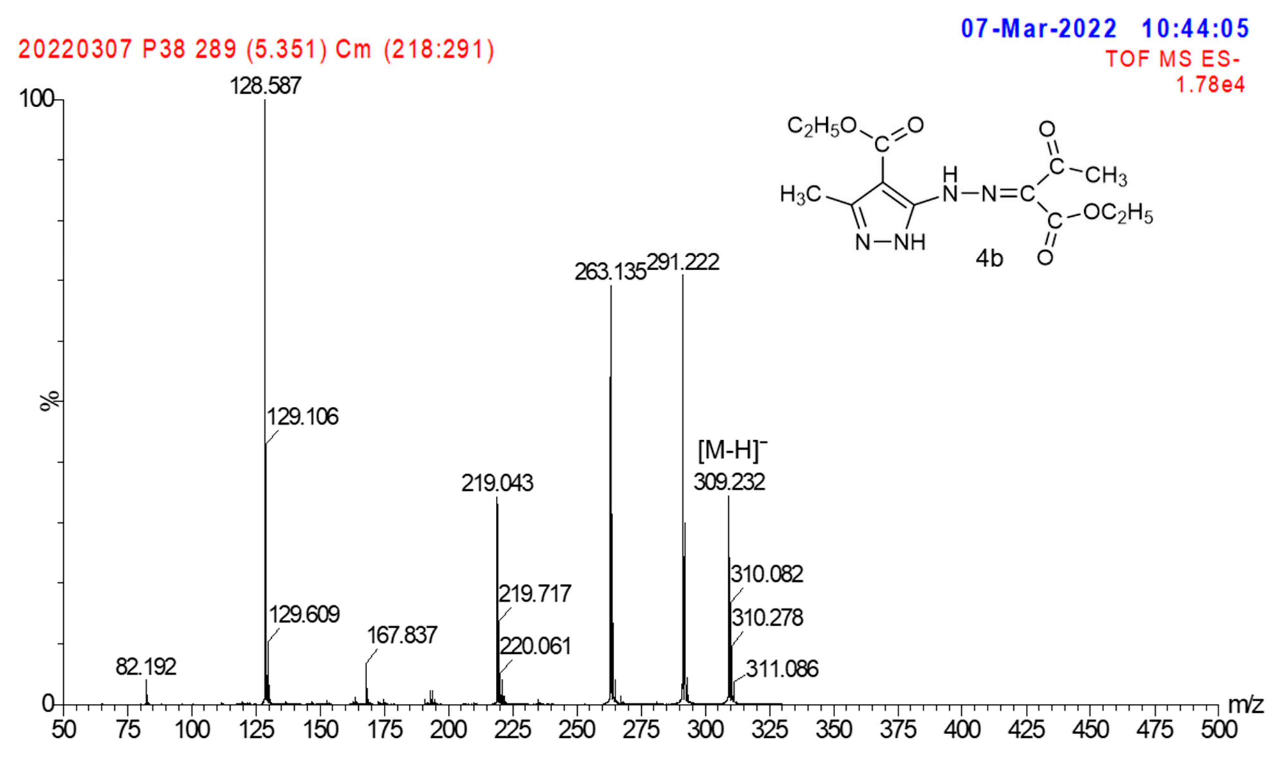

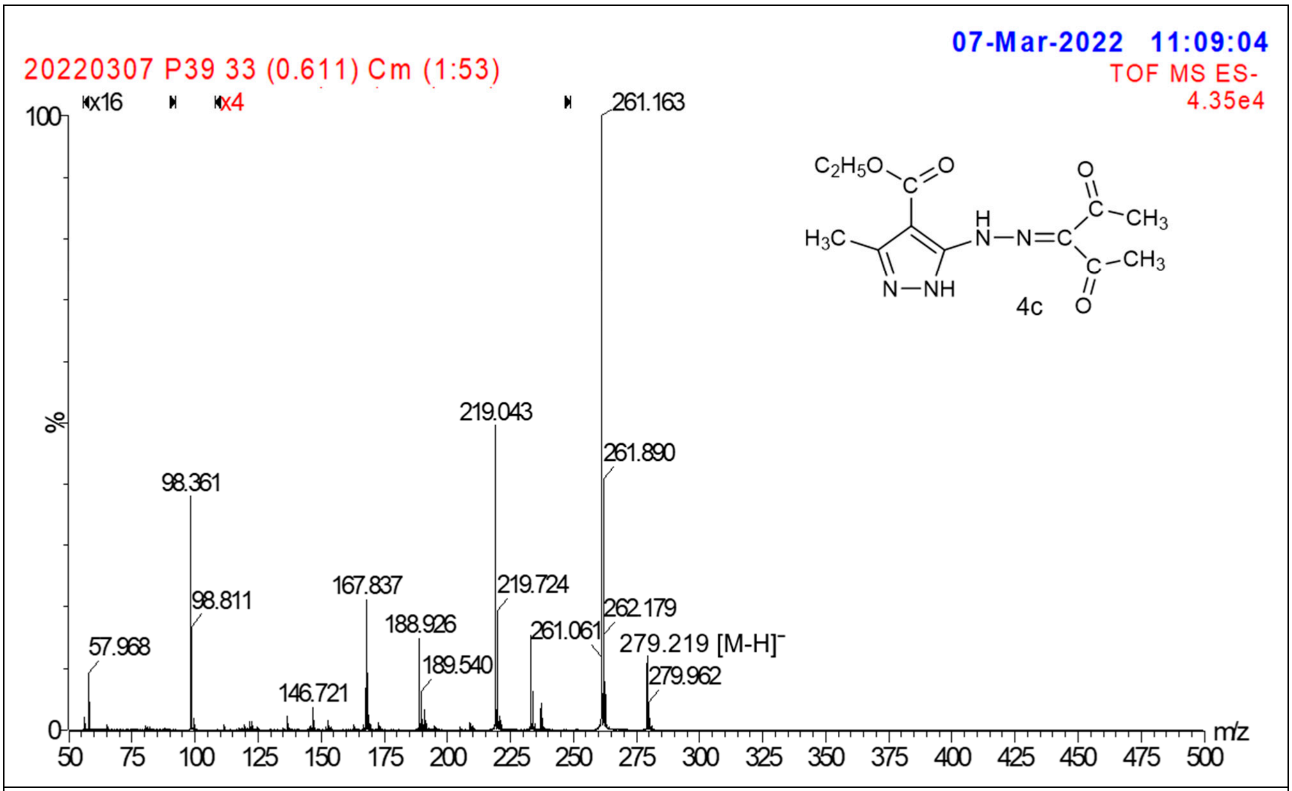
07-Mar-2022 11:09:04
20220307 P39 33 (0.611) Cm (1:53)
TOF MS ES-
4.35e4
x16
x4
261.163
4c
219.043
261.890
98.361
167.837
219.724
98.811
262.179
188.926
261.061
57.968
279.219 [M-H]⁻
189.540
279.962
146.721
%
m/z

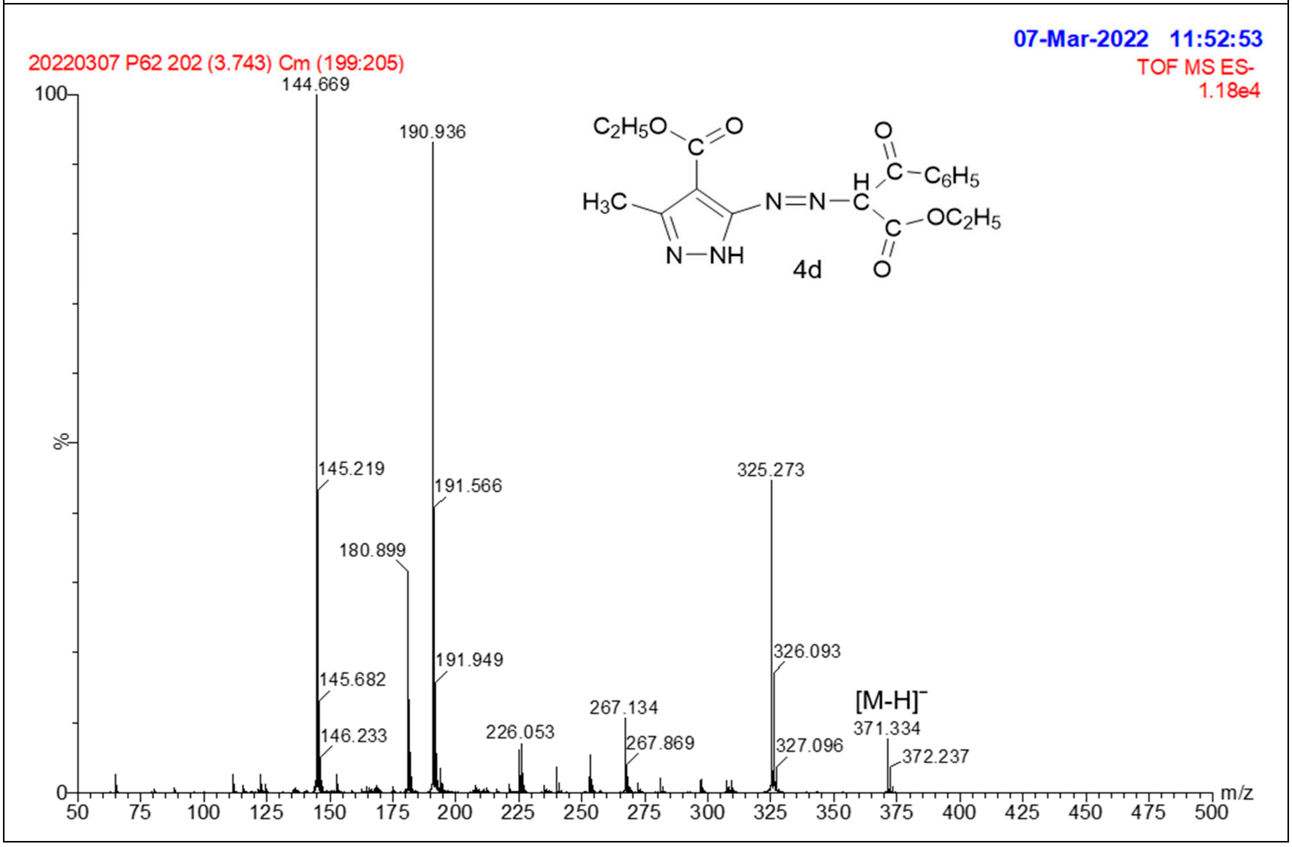
07-Mar-2022 11:52:53
20220307 P62 202 (3.743) Cm (199:205)
TOF MS ES-
1.18e4
144.669
190.936
4d
145.219
191.566
325.273
180.899
326.093
191.949
145.682
267.134
[M-H]⁻
371.334
226.053
267.869
146.233
327.096
372.237
%
m/z

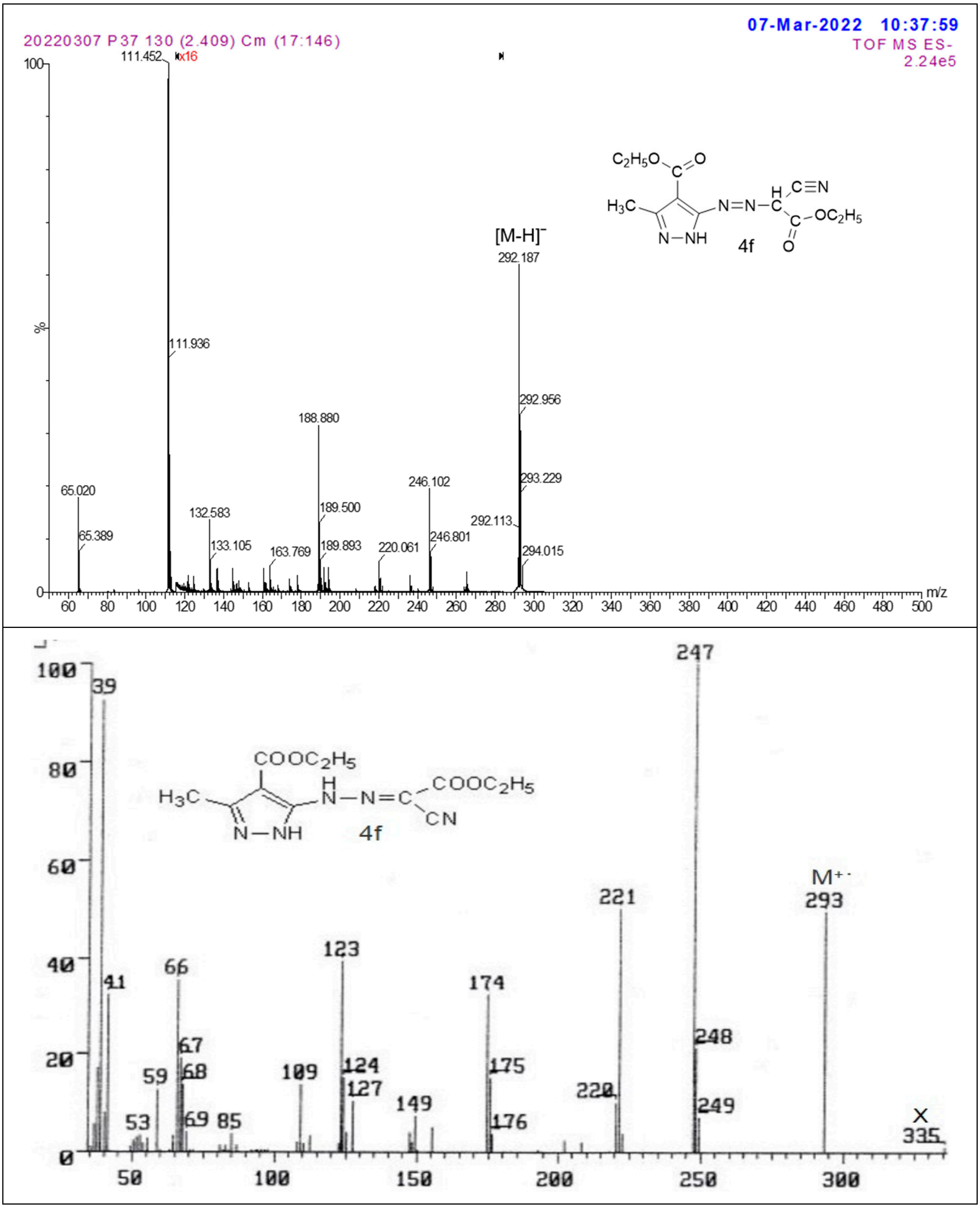

20220307 P37 130 (2.409) Cm (17:146)
07-Mar-2022 10:37:59
TOF MS ES-
2.24e5
x16
%
111.452
111.936
65.020
65.389
132.583
133.105
163.769
188.880
189.500
189.893
220.061
246.102
246.801
292.113
[M-H]⁻
292.187
292.956
293.229
294.015
m/z
4f
247
39
41
53
59
66
67
68
69
85
109
123
124
127
149
174
175
176
220
221
248
249
M+·
293
X
335

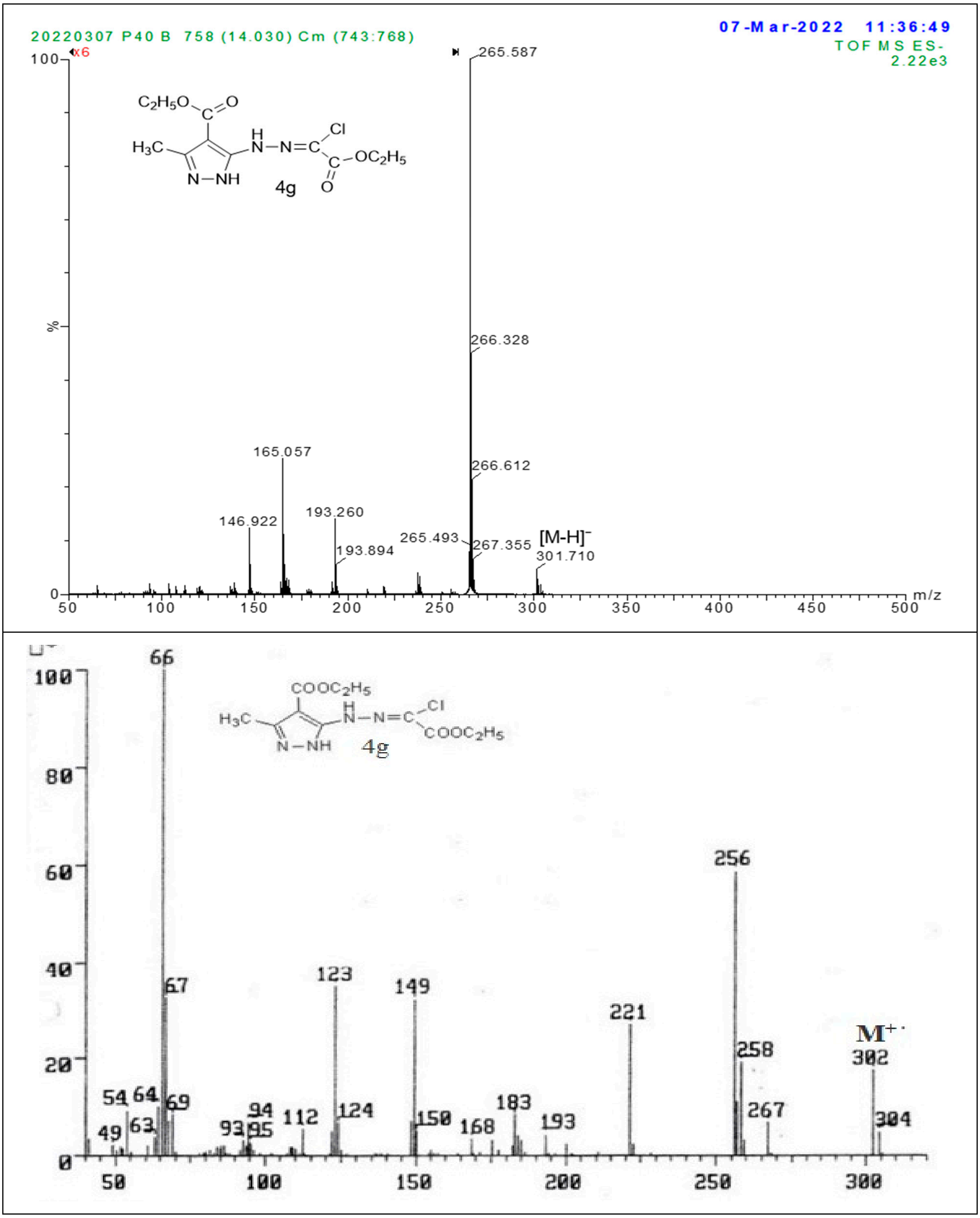
20220307 P40 B 758 (14.030) Cm (743:768)
07-Mar-2022 11:36:49
TOF MS ES-
2.22e3
x6
265.587
266.328
266.612
165.057
146.922
193.260
193.894
265.493
267.355
[M-H]⁻
301.710
%
m/z
4g
M⁺·
66
256
67
123
149
221
258
302
54
64
63
69
49
94
93
95
112
124
150
168
183
193
267
304

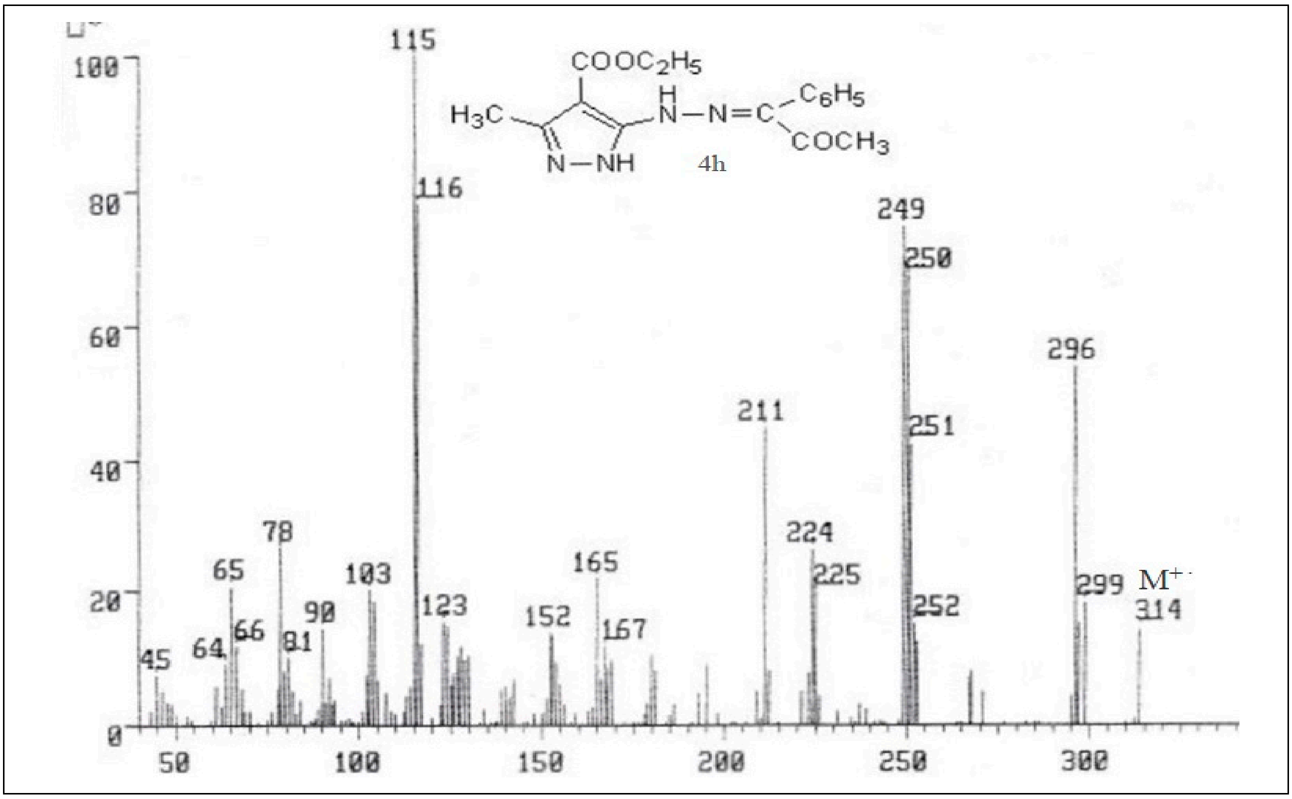
COOC2H5
H3C
N—NH
H
N—N=C
C6H5
COCH3
4h
115
116
249
250
296
211
251
78
65
103
224
225
165
299
M+·
314
123
252
90
152
66
167
81
64
45
100
80
60
40
20
0
50
100
150
200
250
300

Table S7. Experimental FT-IR spectra (in the range/domain 4000 - 400 cm^{-1}) of the azomethylene dyes.

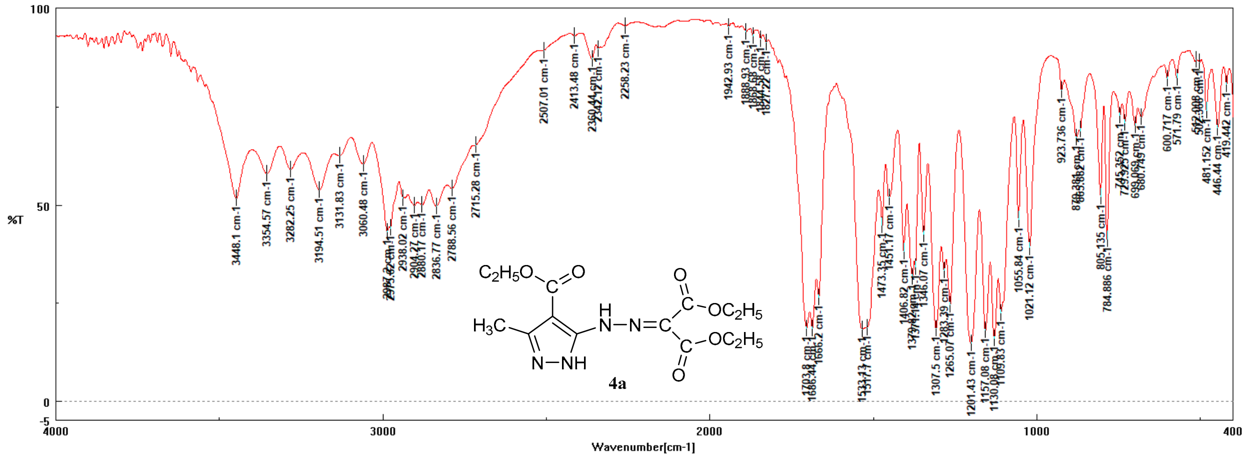

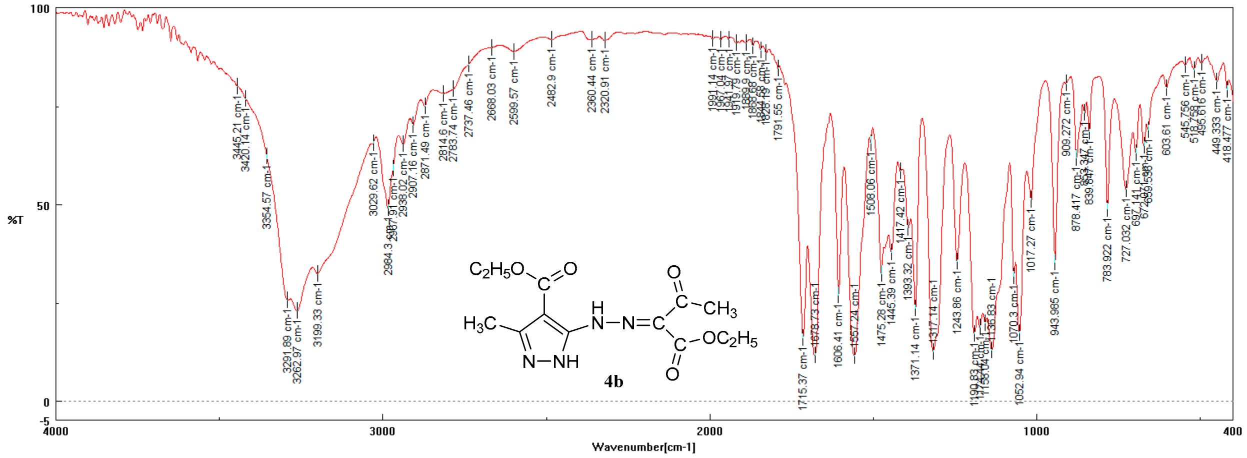

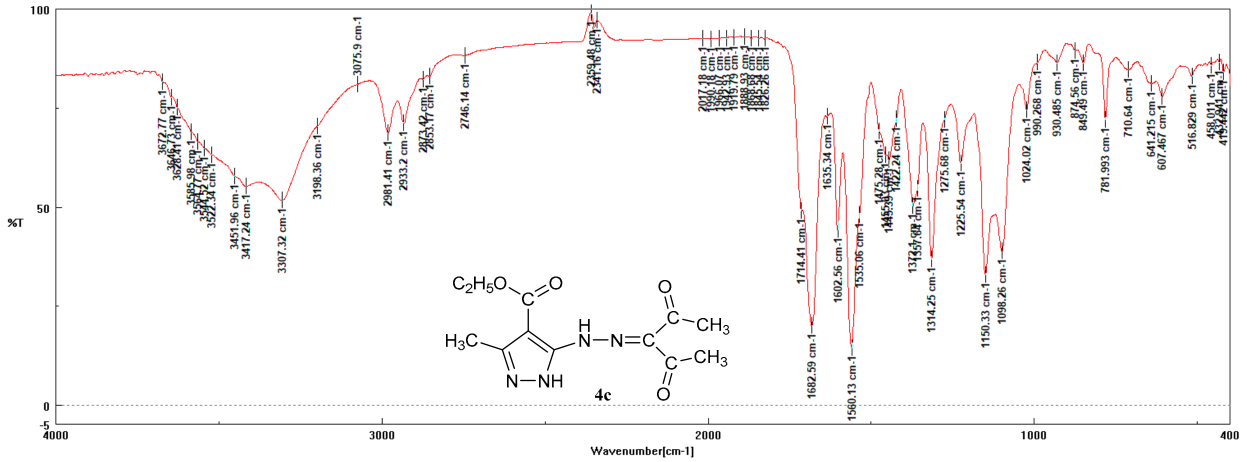

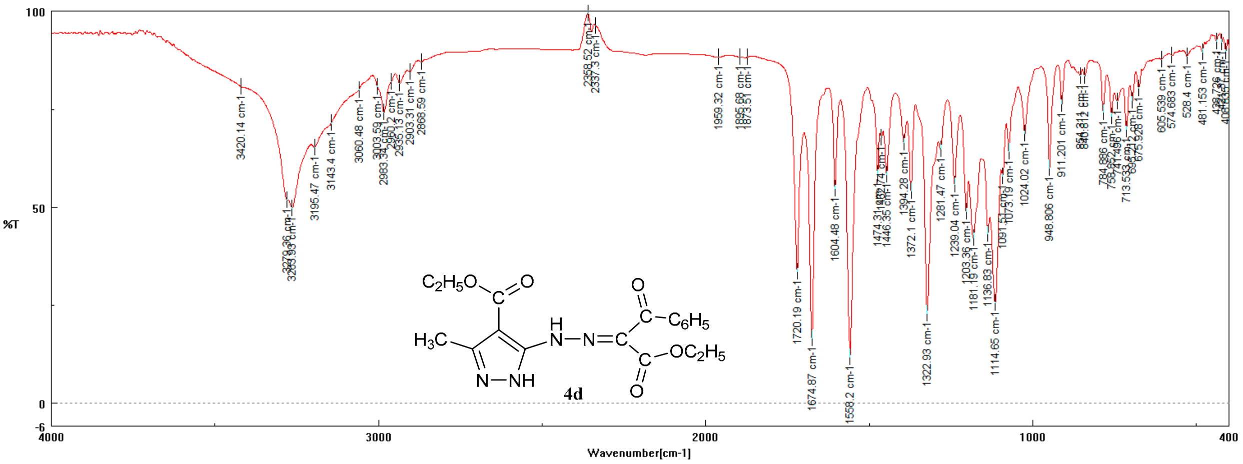

%T
Wavenumber[cm-1]
4d

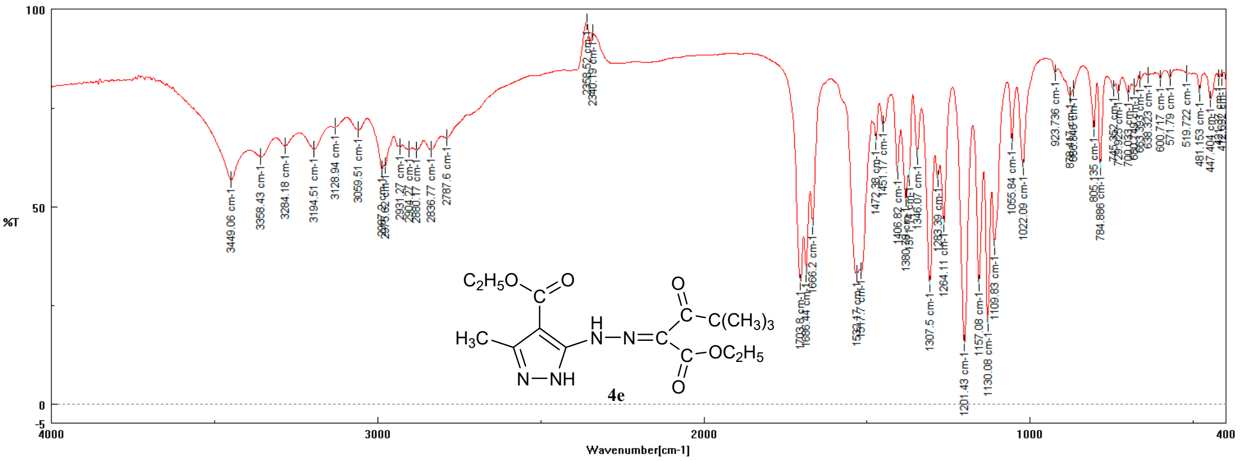

%T
Wavenumber[cm-1]
4e

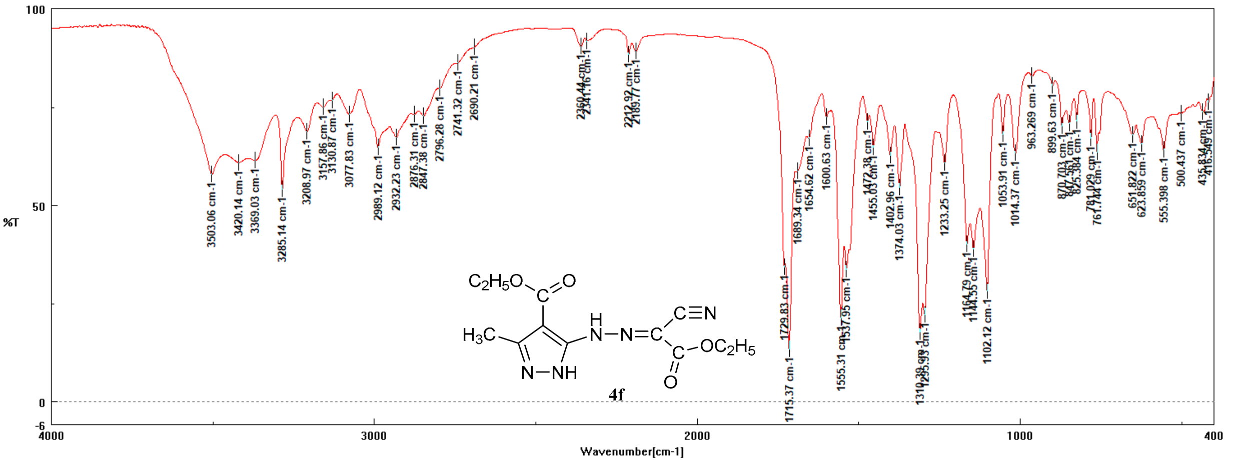

%T
Wavenumber[cm-1]
4f

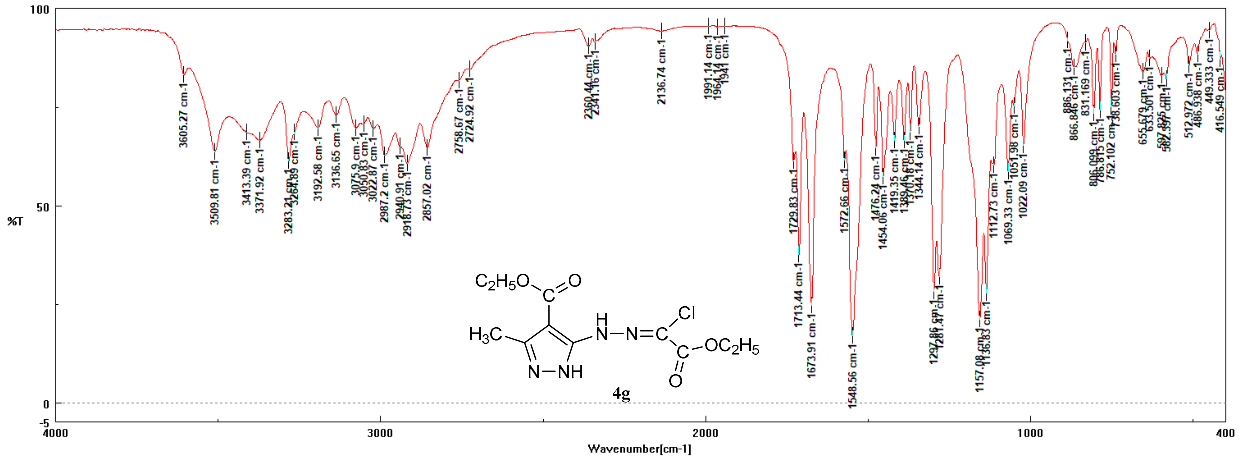
100
50
0
%T
4000
3000
2000
1000
400
Wavenumber[cm-1]
4g

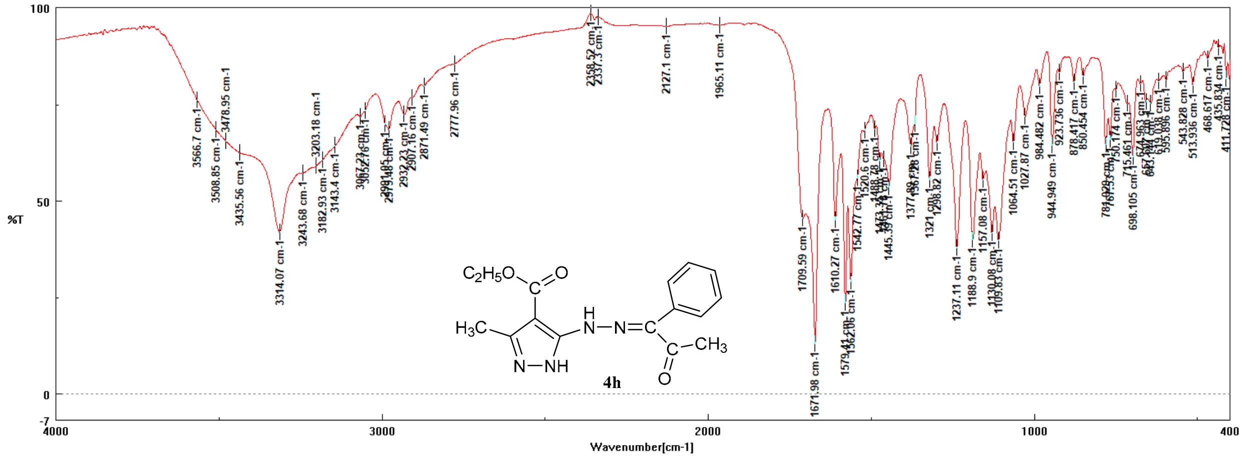
100
50
0
%T
4000
3000
2000
1000
400
Wavenumber[cm-1]
4h

Table S8. Experimental FT-IR spectra (extended in the range/domain 3600 - 2500 and 2500 - 400 cm^{-1}) of the azomethylene dyes.

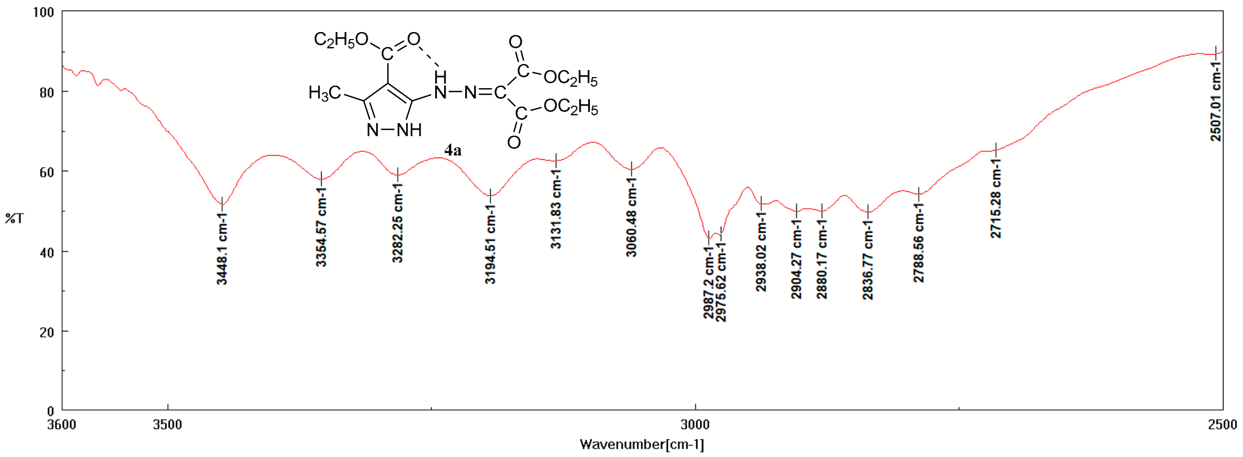

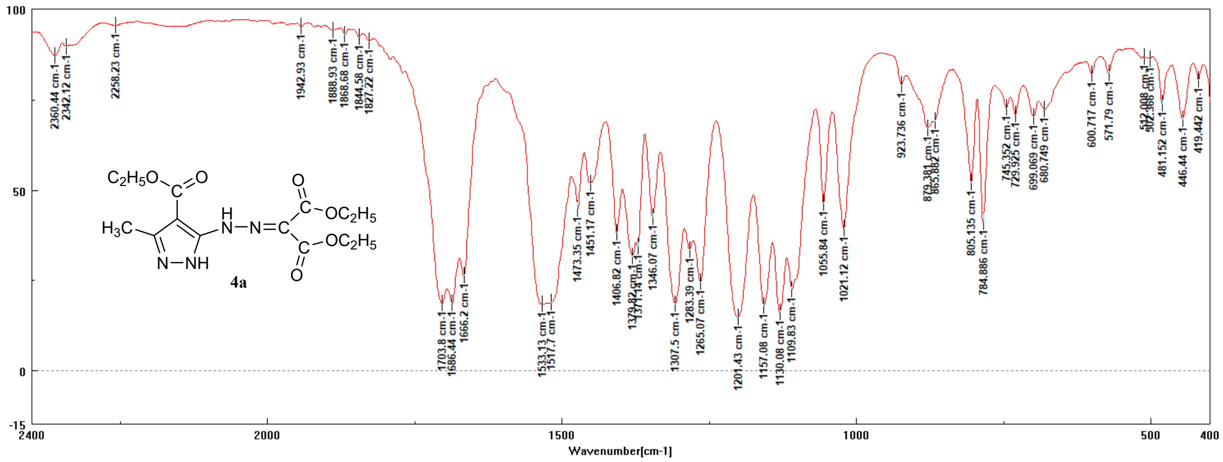

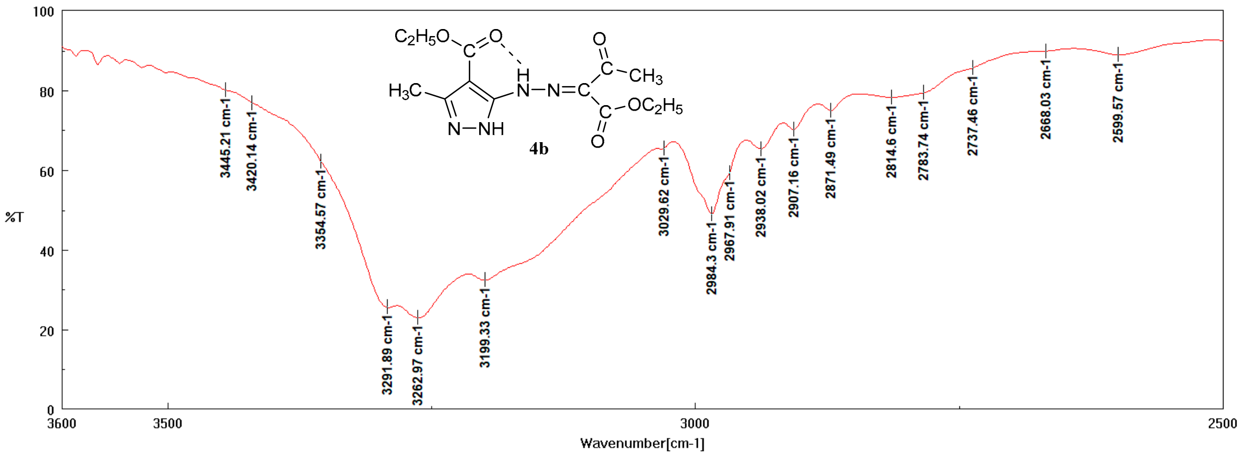

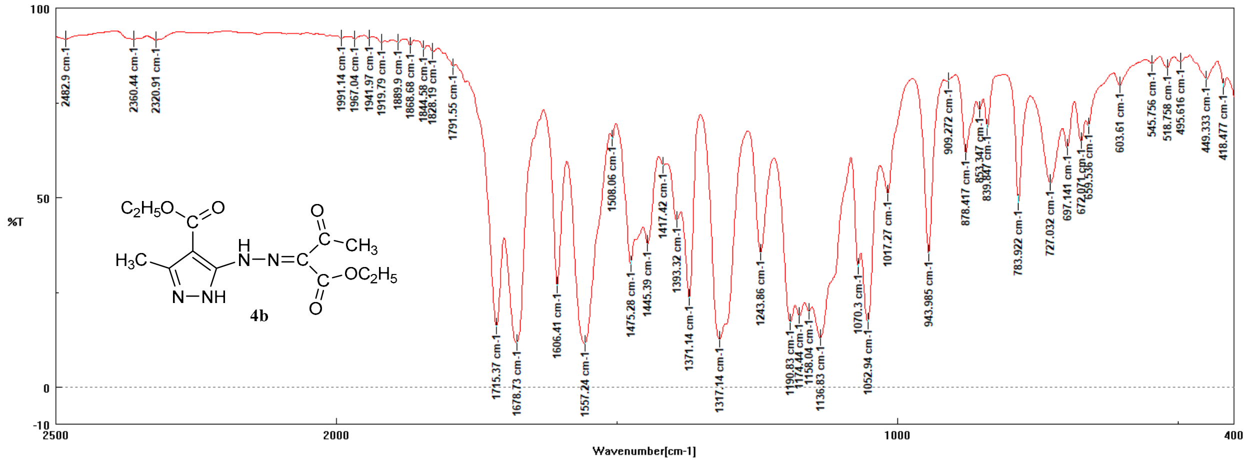
%T
Wavenumber[cm-1]
4b

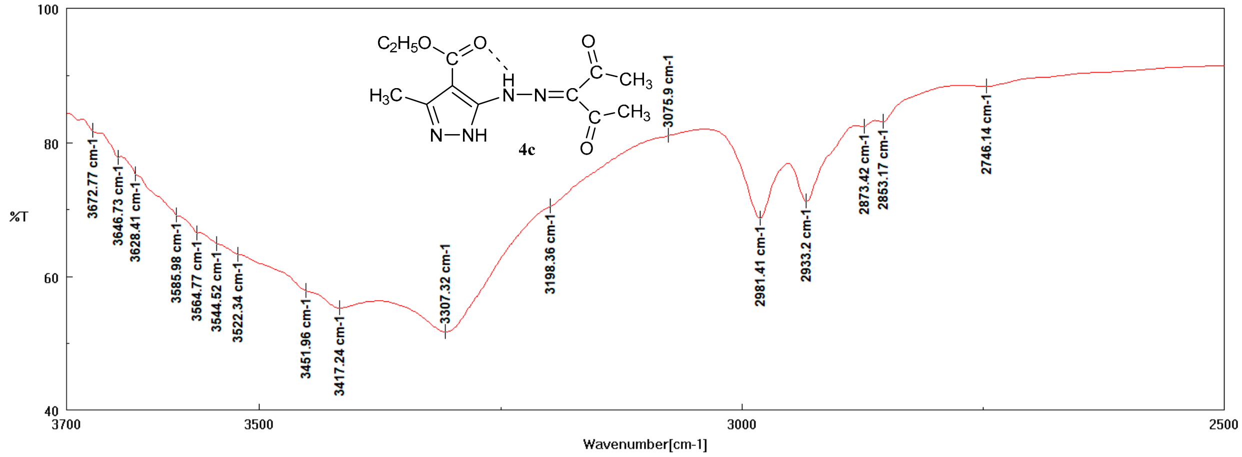
%T
Wavenumber[cm-1]
4c
3672.77 cm-1
3646.73 cm-1
3628.41 cm-1
3585.98 cm-1
3564.77 cm-1
3544.52 cm-1
3522.34 cm-1
3451.96 cm-1
3417.24 cm-1
3307.32 cm-1
3198.36 cm-1
3075.9 cm-1
2981.41 cm-1
2933.2 cm-1
2873.42 cm-1
2853.17 cm-1
2746.14 cm-1

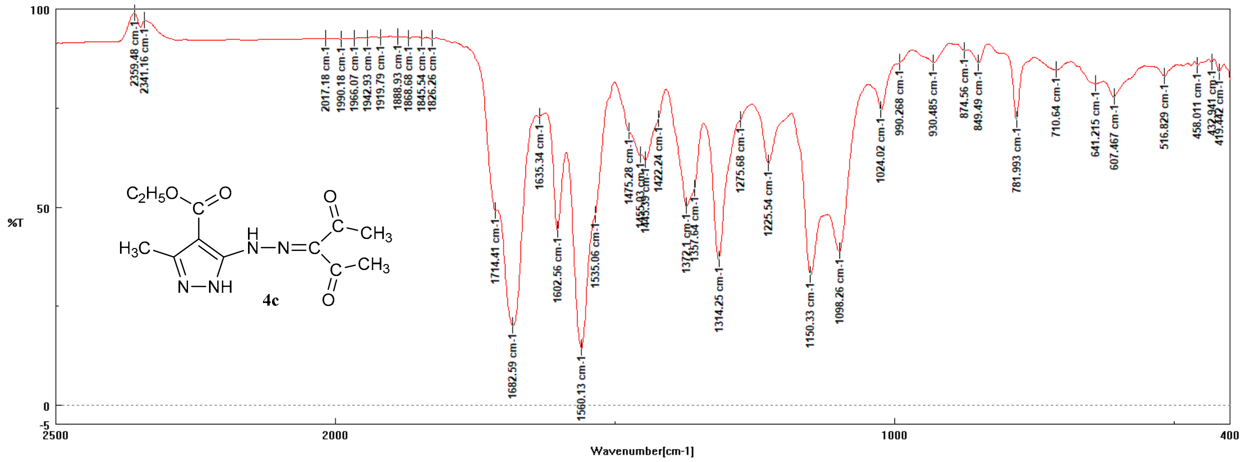
%T
Wavenumber[cm-1]
4c

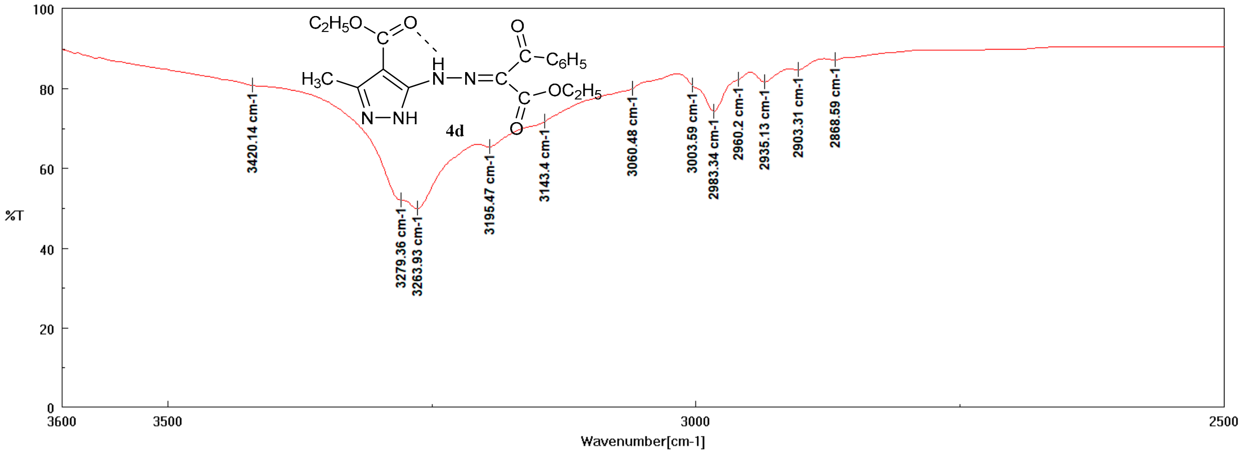

4d
%T
Wavenumber[cm-1]
3420.14 cm-1
3279.36 cm-1
3263.93 cm-1
3195.47 cm-1
3143.4 cm-1
3060.48 cm-1
3003.59 cm-1
2983.34 cm-1
2960.2 cm-1
2935.13 cm-1
2903.31 cm-1
2868.59 cm-1

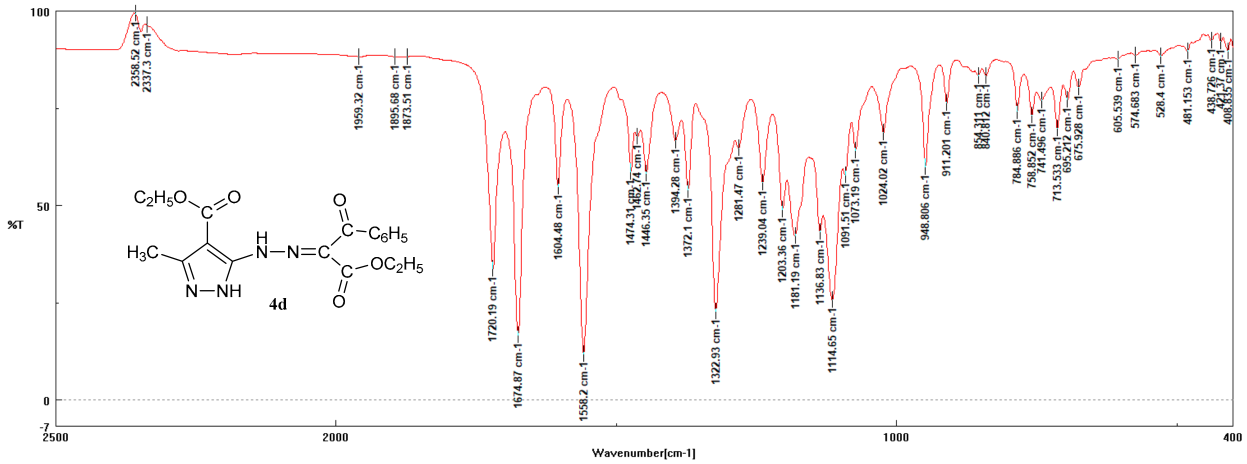

4d
%T
Wavenumber[cm-1]
2358.52 cm-1
2337.3 cm-1
1959.32 cm-1
1895.68 cm-1
1873.51 cm-1
1720.19 cm-1
1674.87 cm-1
1604.48 cm-1
1558.2 cm-1
1474.31 cm-1
1462.74 cm-1
1446.35 cm-1
1394.28 cm-1
1372.1 cm-1
1322.93 cm-1
1281.47 cm-1
1239.04 cm-1
1203.36 cm-1
1181.19 cm-1
1136.83 cm-1
1114.65 cm-1
1091.51 cm-1
1073.19 cm-1
1024.02 cm-1
948.806 cm-1
911.201 cm-1
784.886 cm-1
758.852 cm-1
741.496 cm-1
713.533 cm-1
695.212 cm-1
675.928 cm-1
605.539 cm-1
574.683 cm-1
528.4 cm-1
481.153 cm-1

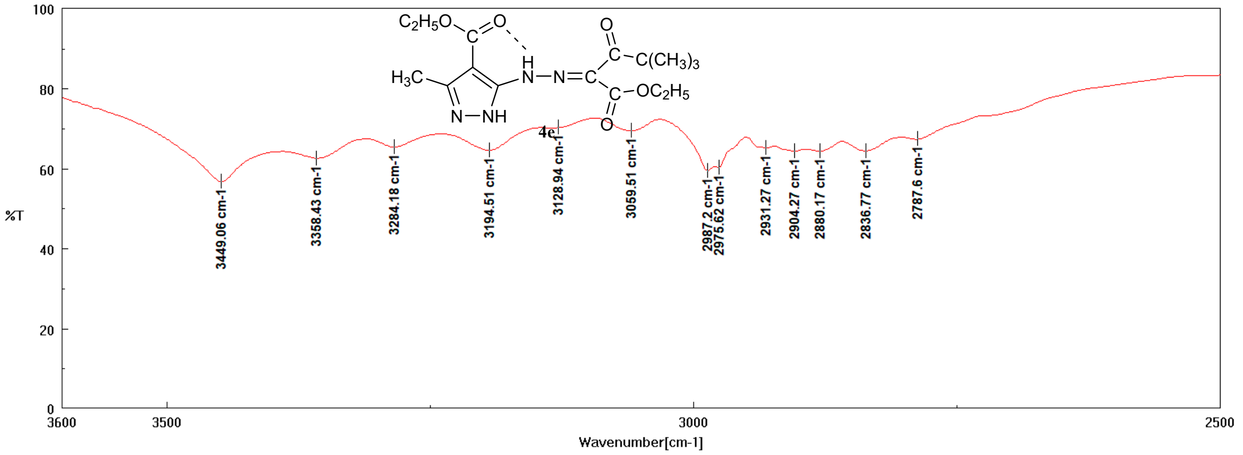

4e
%T
Wavenumber[cm-1]
3449.06 cm-1
3358.43 cm-1
3284.18 cm-1
3194.51 cm-1
3128.94 cm-1
3059.51 cm-1
2987.2 cm-1
2975.62 cm-1
2931.27 cm-1
2904.27 cm-1
2880.17 cm-1
2836.77 cm-1
2787.6 cm-1

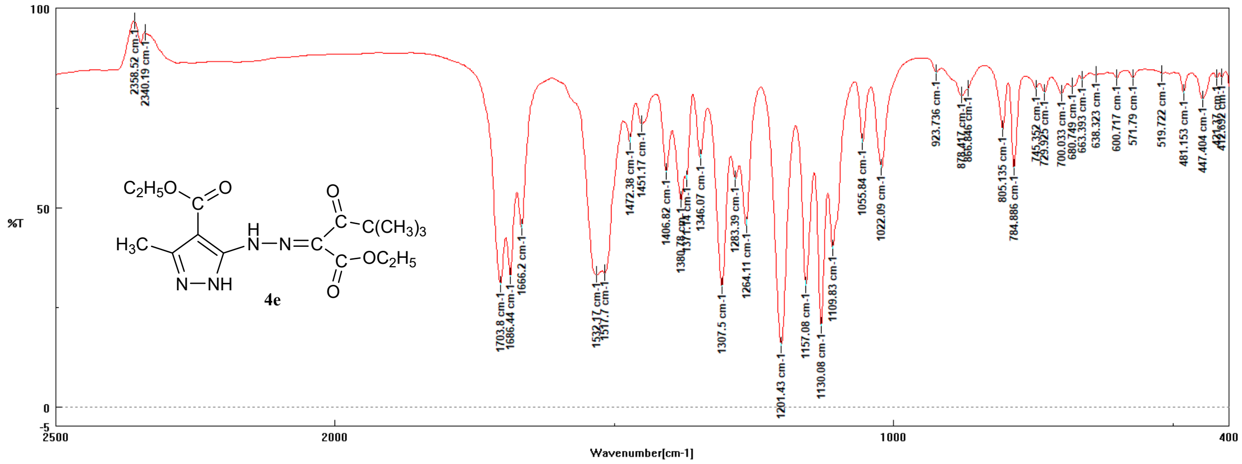
100
50
0
-5
%T
2500
2000
1000
400
Wavenumber[cm-1]
4e
2358.52 cm-1
2340.19 cm-1
1703.8 cm-1
1686.44 cm-1
1666.2 cm-1
1532.17 cm-1
1517.7 cm-1
1472.38 cm-1
1451.17 cm-1
1406.82 cm-1
1346.07 cm-1
1307.5 cm-1
1283.39 cm-1
1264.11 cm-1
1201.43 cm-1
1157.08 cm-1
1130.08 cm-1
1109.83 cm-1
1055.84 cm-1
1022.09 cm-1
923.736 cm-1
805.135 cm-1
784.886 cm-1
638.323 cm-1
600.717 cm-1
571.79 cm-1
519.722 cm-1
481.153 cm-1
447.404 cm-1

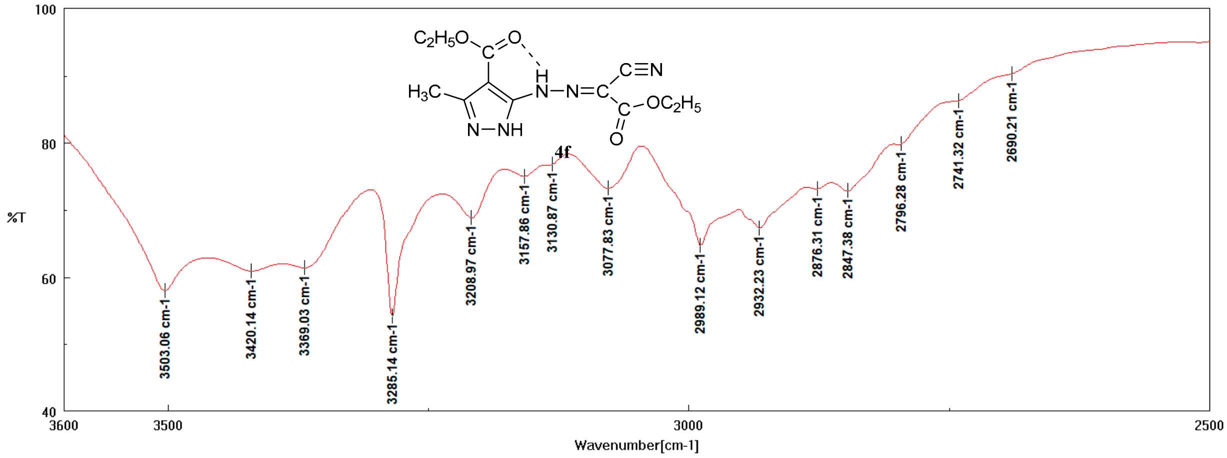
100
80
60
40
%T
3600
3500
3000
2500
Wavenumber[cm-1]
4f
3503.06 cm-1
3420.14 cm-1
3369.03 cm-1
3285.14 cm-1
3208.97 cm-1
3157.86 cm-1
3130.87 cm-1
3077.83 cm-1
2989.12 cm-1
2932.23 cm-1
2876.31 cm-1
2847.38 cm-1
2796.28 cm-1
2741.32 cm-1
2690.21 cm-1

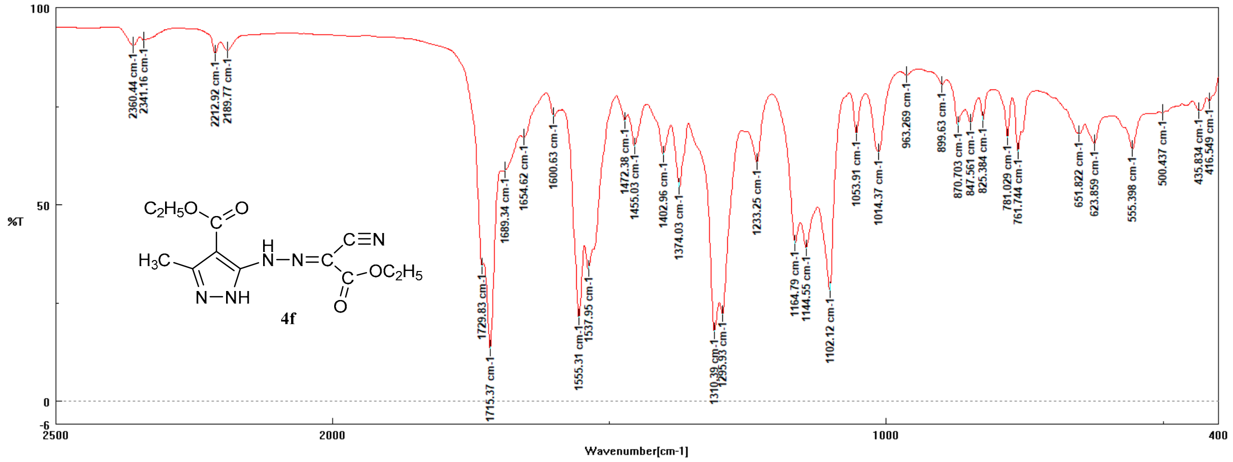
100
50
0
-6
%T
2500
2000
1000
400
Wavenumber[cm-1]
4f
2360.44 cm-1
2341.16 cm-1
2212.92 cm-1
2189.77 cm-1
1729.83 cm-1
1715.37 cm-1
1689.34 cm-1
1654.62 cm-1
1600.63 cm-1
1555.31 cm-1
1537.95 cm-1
1472.38 cm-1
1455.03 cm-1
1402.96 cm-1
1374.03 cm-1
1233.25 cm-1
1164.79 cm-1
1144.55 cm-1
1102.12 cm-1
1053.91 cm-1
1014.37 cm-1
963.269 cm-1
899.63 cm-1
870.703 cm-1
847.561 cm-1
825.384 cm-1
781.029 cm-1
761.744 cm-1
651.822 cm-1
623.859 cm-1
555.398 cm-1
500.437 cm-1
435.834 cm-1
416.549 cm-1

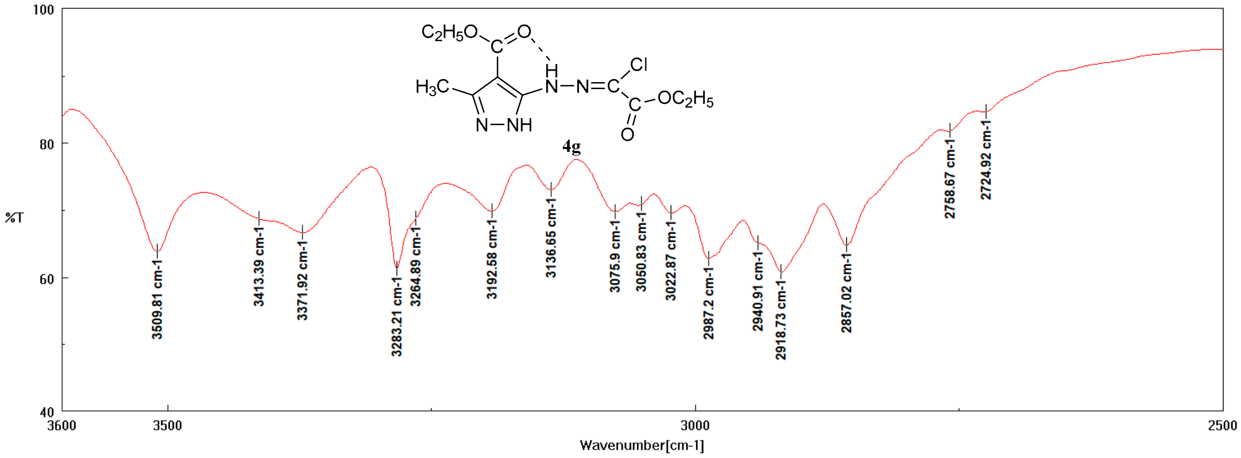
4g
%T
Wavenumber[cm-1]
3509.81 cm-1
3413.39 cm-1
3371.92 cm-1
3283.21 cm-1
3264.89 cm-1
3192.58 cm-1
3136.65 cm-1
3075.9 cm-1
3050.83 cm-1
3022.87 cm-1
2987.2 cm-1
2940.91 cm-1
2918.73 cm-1
2857.02 cm-1
2758.67 cm-1
2724.92 cm-1

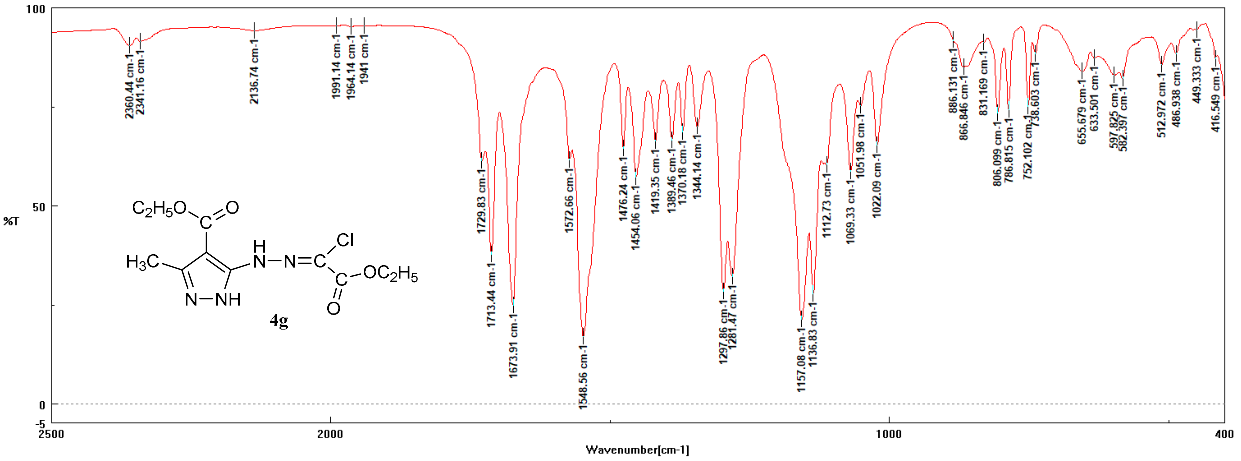
4g
%T
Wavenumber[cm-1]
2360.44 cm-1
2341.16 cm-1
2136.74 cm-1
1991.14 cm-1
1964.14 cm-1
1941 cm-1
1729.83 cm-1
1713.44 cm-1
1673.91 cm-1
1572.66 cm-1
1548.56 cm-1
1476.24 cm-1
1454.06 cm-1
1419.35 cm-1
1389.46 cm-1
1370.18 cm-1
1344.14 cm-1
1297.86 cm-1
1281.47 cm-1
1157.08 cm-1
1136.83 cm-1
1112.73 cm-1
1069.33 cm-1
1051.98 cm-1
1022.09 cm-1
886.131 cm-1
866.846 cm-1
831.169 cm-1
806.099 cm-1
786.815 cm-1
752.102 cm-1
738.603 cm-1
655.679 cm-1
633.501 cm-1
597.825 cm-1
582.397 cm-1
512.972 cm-1
486.938 cm-1
449.333 cm-1
416.549 cm-1

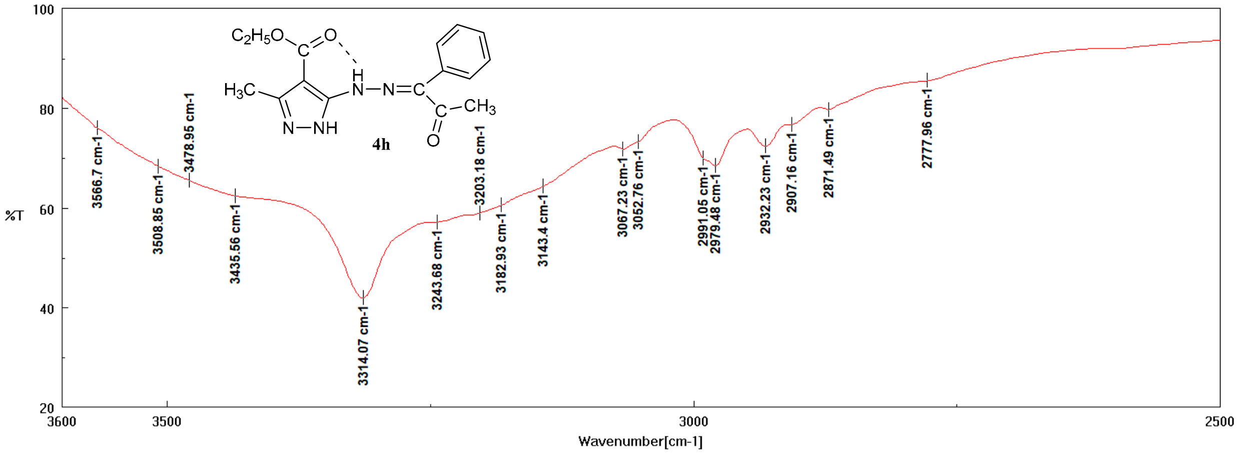
4h
%T
Wavenumber[cm-1]
3566.7 cm-1
3508.85 cm-1
3478.95 cm-1
3435.56 cm-1
3314.07 cm-1
3243.68 cm-1
3203.18 cm-1
3182.93 cm-1
3143.4 cm-1
3067.23 cm-1
3052.76 cm-1
2991.05 cm-1
2979.48 cm-1
2932.23 cm-1
2907.16 cm-1
2871.49 cm-1
2777.96 cm-1

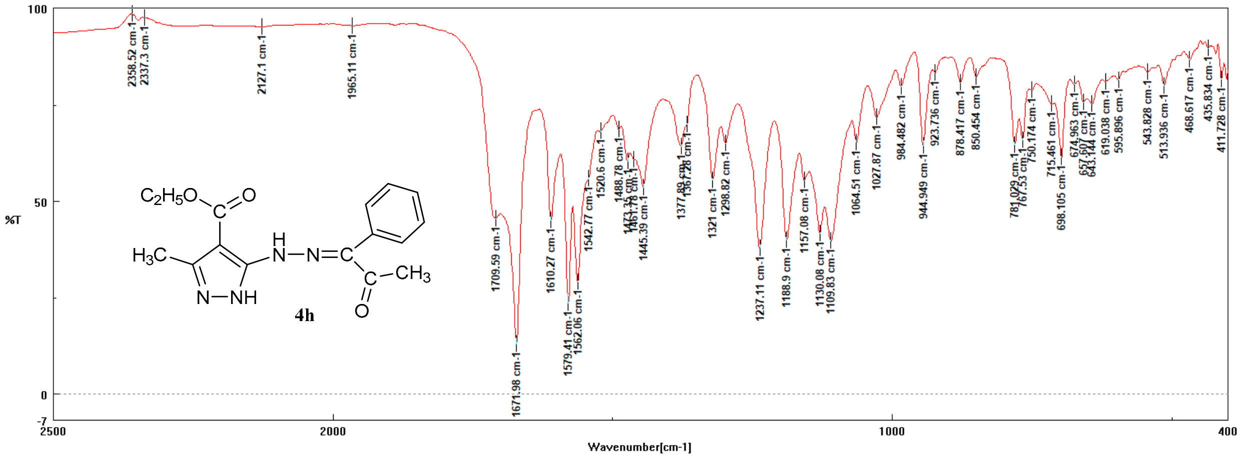

100
50
0
-7
%T
2500
2000
1000
400
Wavenumber[cm-1]
4h
2358.52 cm-1
2337.3 cm-1
2127.1 cm-1
1965.11 cm-1
1709.59 cm-1
1671.98 cm-1
1610.27 cm-1
1579.41 cm-1
1562.06 cm-1
1542.77 cm-1
1520.6 cm-1
1488.78 cm-1
1445.39 cm-1
1321 cm-1
1298.82 cm-1
1237.11 cm-1
1188.9 cm-1
1157.08 cm-1
1130.08 cm-1
1109.83 cm-1
1064.51 cm-1
1027.87 cm-1
984.482 cm-1
944.949 cm-1
923.736 cm-1
878.417 cm-1
850.454 cm-1
781.029 cm-1
750.174 cm-1
715.461 cm-1
698.105 cm-1
674.963 cm-1
619.038 cm-1
595.896 cm-1
543.828 cm-1
513.936 cm-1
468.617 cm-1
435.834 cm-1
411.728 cm-1

Table S9. Calculated bioconcentration factor (BCF), oral rodent toxicity (LD_{50}) with toxicity classes and other endpoint toxicities (their probability is included in parenthesis) for the new azomethylene dyes.

Dye	Tautomer	BCF* (L/kg wet-wt)	LD_{50}** (mg/kg)	Toxicity class	Hepatotoxicity (Probability)	Carcinogenicity (Probability)	Immunotoxicity (Probability)	Mutagenicity (Probability)	Cytotoxicity (Probability)
4a1	azo	10	500	4	Inactive (0.54)	Inactive (0.53)	Inactive (0.99)	Inactive (0.51)	Inactive (0.74)
4a2	hydrazo	2.716	300	4	Inactive (0.53)	Inactive (0.53)	Inactive (0.99)	Inactive (0.57)	Inactive (0.71)
4b1	azo	10	500	4	Inactive (0.52)	Inactive (0.55)	Inactive (0.99)	Inactive (0.5)	Inactive (0.75)
4b2	hydrazo	3.162	200	4	Inactive (0.51)	Inactive (0.54)	Inactive (0.99)	Inactive (0.56)	Inactive (0.72)
4c1	azo	10	500	4	Inactive (0.52)	Inactive (0.55)	Inactive (0.99)	Active (0.51)	Inactive (0.76)
4c2	hydrazo	3.162	150	3	Inactive (0.51)	Inactive (0.54)	Inactive (0.99)	Inactive (0.54)	Inactive (0.74)
4d1	azo	2.6	500	4	Active (0.5)	Inactive (0.55)	Inactive (0.99)	Inactive (0.52)	Inactive (0.78)
4d2	hydrazo	9.288	200	3	Active (0.51)	Inactive (0.55)	Inactive (0.99)	Inactive (0.59)	Inactive (0.76)
4e1	azo	10	500	4	Inactive (0.51)	Inactive (0.56)	Inactive (0.99)	Inactive (0.51)	Inactive (0.72)
4e2	hydrazo	4.05	150	3	Inactive (0.51)	Inactive (0.55)	Inactive (0.99)	Inactive (0.55)	Inactive (0.7)
4f1	azo	10	500	4	Inactive (0.55)	Inactive (0.54)	Inactive (0.99)	Inactive (0.51)	Inactive (0.72)
4f2	hydrazo	14.2	300	3	Inactive (0.54)	Inactive (0.53)	Inactive (0.99)	Inactive (0.56)	Inactive (0.69)
4g1	azo	10	500	4	Inactive (0.53)	Inactive (0.58)	Inactive (0.99)	Inactive (0.53)	Inactive (0.72)
4g2	hydrazo	3.807	300	3	Inactive (0.53)	Inactive (0.57)	Inactive (0.99)	Inactive (0.57)	Inactive (0.69)
4h1	azo	10	500	4	Inactive (0.52)	Inactive (0.53)	Inactive (0.99)	Active (0.5)	Inactive (0.77)
4h2	hydrazo	16.43	1000	4	Inactive (0.51)	Inactive (0.53)	Inactive (0.99)	Inactive (0.55)	Inactive (0.75)

* BCF > 5000 corresponds to high bioconcentration potential; 1000 < BCF < 5000 – moderate bioconcentration potential; BCF < 1000 – low bioconcentration potential. ** $LD_{50} \leq 5$ (Class I, fatal if swallowed); $5 < LD_{50} \leq 50$ (Class II, fatal if swallowed); $50 < LD_{50} \leq 300$ (Class III, toxic if swallowed); $300 < LD_{50} \leq 2000$ (Class IV, harmful if swallowed); $2000 < LD_{50} \leq 5000$ (Class V, may be harmful if swallowed); $LD_{50} > 5000$ (Class VI, non-toxic).

Reference

1. Pedretti, A.; Mazzolari, A.; Gervasoni, S.; Fumagalli, L.; Vistoli, G. The VEGA suite of programs: A versatile platform for cheminformatics and drug design projects. *Bioinformatics* **2021**, *37*, 1174–1175.
